# Recent Advances in Catalysts for Hydrogen Production: From Fossil-Derived Processes to Sustainable Water Electrolysis and Photocatalysis

**DOI:** 10.1021/acsomega.5c11297

**Published:** 2026-05-06

**Authors:** Guilherme Mateus Bousada, Noemí Silva de Souza, Géssica Dias, Luísa Mazzini, Tatianny de Araujo Andrade, Tiansheng Wang, Gabriel da Silva Souza, Didier Astruc, Renata Lopes Moreira

**Affiliations:** † Department of Chemistry, 28120 Universidade Federal de Viçosa, Viçosa, Minas Gerais 36570-000, Brazil; ‡ Institut des Science Moléculaires, UMR CNRS 5255, 27086 Université de Bordeaux, Talence Cedex 33405, France; § LCC, CNRS UPR 8241 & University of Toulouse, Toulouse Cedex 31077, France

## Abstract

This review provides a thorough summary of recent progress in catalysts used for hydrogen production. It starts with an overview of the importance of hydrogen and its historical background, then looks into catalytic methods for the thermochemical transformation of fossil fuels and organic materials, with a specific emphasis on steam methane reforming (SMR) and ethanol steam reforming (ESR). The review discusses the fundamental processes and approaches to minimize CO_2_ emissions and tackle catalyst deactivation due to sulfur and coking. It also delves into dry reforming and the catalytic breakdown of methane. Furthermore, the review investigates water splitting through electrolysis and photolysis, clarifying key principles, types of catalysts, reaction pathways, and performance assessment criteria. A specific section focuses on biological, biomimetic, and bioinspired catalytic-driven methods for generating hydrogen, along with a brief overview of catalysts involved in hydrogen release from chemical hydrides. Ultimately, the review underscores the increasingly important role of computational techniques, such as density functional theory (DFT) and Artificial Intelligence/Machine Learning (AI/ML), in the rational design of innovative catalytic materials. While acknowledging the extensive field of hydrogen production, this review seeks to offer an insightful perspective on catalytic elements, highlighting current trends, challenges, and future opportunities in the pursuit of sustainable hydrogen generation. The final remarks stress the importance of developing practical and scalable catalysts, taking environmental impacts into account, standardizing the reporting of catalytic performance, and recognizing the significant role often played by catalyst supports.

## Graphical abstract

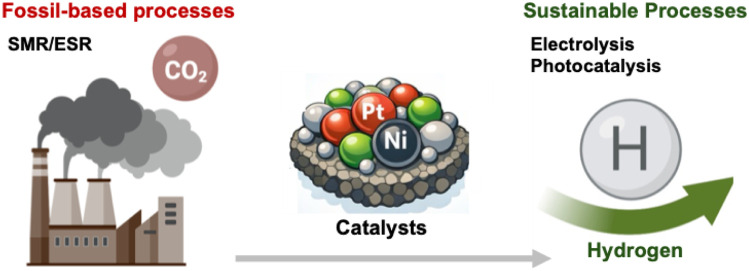

## 1 Introduction

Hydrogen, the oldest element in the universe (∼13 billion years old) and the most abundant (∼92% by mass), is found on Earth primarily in two forms: as molecular hydrogen within the Earth’s crust—France alone is estimated to contain 50 million tons—and more commonly in compounds like water (bonded to oxygen) and fossil fuels (bonded to carbon).^1,2^ Currently, the combustion of fossil fuels (eqs 1 and 2) fulfills 80% of humanity’s energy needs, a process that releases CO_2_, the main greenhouse gas responsible for global warming, along with toxic nanoparticles (eq 3), thereby contributing significantly to environmental pollution (eqs 1–3). In contrast, burning hydrogen gas generates only harmless water (eq 4). Consequently, extensive research has been undertaken over recent decades to produce and utilize hydrogen as an energy vector to replace fossil fuels.^3^

1 $${\mathrm{CH}}_{4}{+\mathrm{2 O}}_{2}\to {\mathrm{CO}}_{2}{+\mathrm{2 H}}_{2}{O+\mathrm{810 kJ mol}}^{\mathrm{-1}}$$

2 $$C_{n}H_{2n+2}+\left(1.5n+0.5\right)O_{2}\to {\mathrm{n CO}}_{2}+\left(n+1\right)H_{2}O+\Delta G\left({\mathrm{610 kJ mol}}^{\mathrm{-1}}{\mathrm{per‐CH}}_{2}\mathrm{‐unit}\right)$$

3 $$C_{\left(s\right)}{+O}_{2}\to {\mathrm{CO}}_{2}{+\mathrm{300 kJ mol}}^{\mathrm{-1}}$$

4 $$H_{2}{+1/2O}_{2}\to H_{2}{O+\mathrm{286 kJ mol}}^{\mathrm{-1}}$$

Historically utilized in “town gas” mixtures in the 19th century, hydrogen has evolved from a niche aerospace fuel to a central pillar of modern decarbonization strategies.^4,5^ More recently, in the 21st century, hydrogen is employed in fuel cells, which function as the reverse of water electrolysis, generating electricity when combined with oxygen (O_2_) from the air. This clean technology is currently in prototype vehicles, including cars, buses, boats, and even airplanes.^6^

The adoption of clean hydrogen fuel cells presents a geopolitical challenge, as clean hydrogen production relies on advanced and costly technologies involving nuclear energy or renewable sources such as solar, wind, hydroelectric, geothermal, and bioenergy. Unfortunately, progress toward sustainable energy solutions has been slow, resulting in continued dependence on fossil fuels in the forthcoming decades. Nevertheless, hydrogen stands out as a promising sustainable energy source that is likely to become more and more vital in the context of global warming and pollution.^5^

The current use of hydrogen gas is dominated by oil refining, corresponding to 47%, closely followed by ammonia production, corresponding to 46%. The remaining 7% can be divided into 4% for methanol production, and only 3% for various other applications such as mobility, electronics, metals, and food production.^7,8^

A common aspect of all hydrogen production methods is the essential role played by catalysts, which govern reaction pathways and influence process efficiency. The catalyst must exhibit high stability under harsh reaction conditions and maintain resistance to undesired side reactions, particularly those leading to carbon deposition.^9^ The present article summarizes both fossil and nonfossil sources of catalytic hydrogen synthesis. It provides a panoramic view of the latest research on the catalysts used for hydrogen production between 2020 and 2025. This review is based on a comprehensive survey of peer-reviewed studies from 2020 to 2025, focused on indexed journals in Energy, Catalysis, Sustainable Materials, and related fields. Literature was selected through keyword searches related to “hydrogen production,” “catalysts,” “steam reforming,” “water electrolysis,” “photocatalysis,” and “biohydrogen,” emphasizing recent experimental advances and computational studies. Catalysts were classified by production route and compared. Although important, this paper will not delve into hydrogen purification, storage, distribution, and other engineering-related topics concerning hydrogen handling. There is a rich literature in this area that can be consulted for further information^10–21^

This review focuses on the research on catalysts applied for hydrogen production, including steam reforming, water splitting through electrolysis and photolysis, and inorganic hydride hydrolysis, as well as cutting-edge topics such as bioinspired catalysis and computational catalyst design using artificial intelligence/machine learning (AI/ML) and Density Functional Theory (DFT). The level of mechanistic detail presented varies across catalytic systems due to differences in the availability of experimental and literature data, as well as the varying technological maturity of each process. Methane Steam Reforming (MSR) is the most mature and widely implemented industrially, while Partial Oxidation of Methane and Autothermal Reforming are also well-established but slightly less widespread. Water electrolysis has reached pilot and limited industrial scales, though at higher cost than MSR. Photocatalytic and photoelectrochemical water splitting remain in the research stage with low TRL, and bioinspired or enzymatic catalysis is highly experimental. Inorganic hydride hydrolysis (e.g., ammonia borane or formate hydrolysis) is primarily laboratory-scale but relevant for green hydrogen production. Some aspects of these processes will be briefly presented due to their relevance. Scalability aspects are not addressed in depth, reflecting the differences in technological maturity among the processes. The primary objective of this review is to critically synthesize recent catalytic advances across hydrogen production technologies, emphasizing structure-performance relationships, mechanistic insights, sustainability and economic considerations, and computational innovations in catalyst design, while providing an overview of the main concepts behind the different techniques.

This review does not aim to be an exhaustive encyclopedia of every catalyst utilized for hydrogen production; rather, it offers a panoramic and insightful perspective on the latest research. Many existing reviews tend to concentrate on a single hydrogen production method, such as steam methane reforming or water splitting, often delving deeply into specialized details. Unlike prior reviews that focus narrowly on single hydrogen production methods, this work synthesizes advances from multiple hydrogen generation pathways, integrating computational breakthroughs with emerging bioinspired and hybrid catalytic systems. This approach effectively addresses a significant gap in the literature by delivering a cohesive and accessible overview of current trends, challenges, and future opportunities in hydrogen generation. By emphasizing the interconnections among these diverse methods and highlighting the critical role of computational techniques, we aspire to offer a valuable resource for both newcomers to the field and experienced researchers seeking a comprehensive understanding of global efforts in this domain.

## 2 Meeting Global Energy Needs: The Shift toward Sustainability

The rise in global energy demand over the past few decades has been a serious issue for industries and governments alike, largely due to population growth and the economic development push (Figure 1)^22^ According to the International Energy Agency (IEA), energy demand is expected to increase by approximately 25% by 2040 compared to current levels. This growth is attributed to various factors, including industrialization, urbanization, transportation, and agriculture.^23,24^

**Figure 1:**
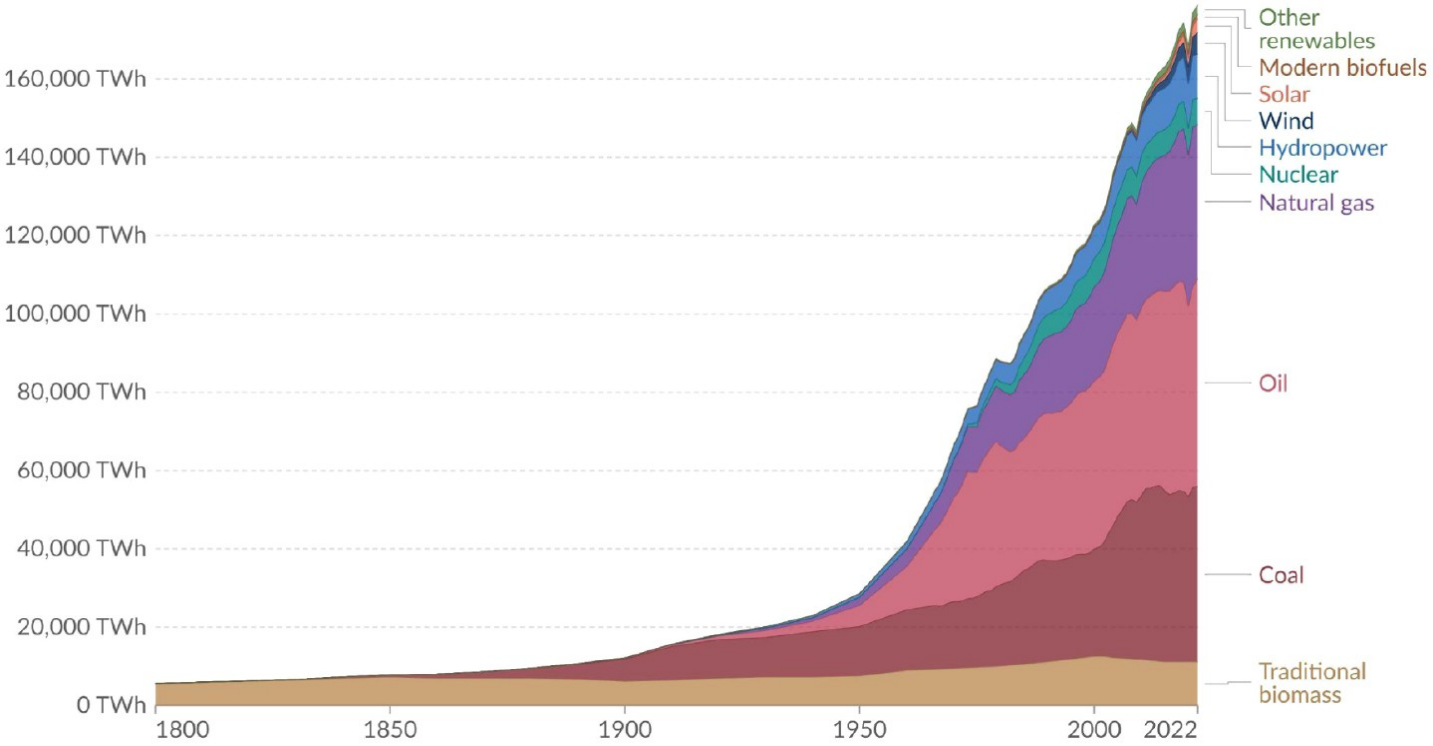
Global primary energy consumption by source. Energy Institute—Statistical Review of World Energy (2023); Smil (2017). OurWorldInData.org/energy. Licensed under a Creative Commons Attribution 4.0 International License, CC BY.

The transportation sector is a key contributor to the rising energy demand. With the increase in motor vehicle ownership worldwide, the need for fossil fuels such as gasoline and diesel has risen significantly. Additionally, the rapidly expanding aviation sector further elevates the demand for these fuels. Furthermore, the industrial sector plays a vital role in global energy consumption. The expansion of industries and the growing production of consumer goods necessitate substantial energy to operate machinery and industrial processes. As a result, developing countries are making significant investments in industrial infrastructure, thereby increasing overall energy demand^23^

However, as society increasingly recognizes the environmental impact of traditional energy sources, their limited availability, and fluctuating oil prices, the demand for more sustainable alternatives is growing. This movement aligns with a global shift toward low-carbon energy solutions, leading to increased investments in solar, wind power, hydroelectric, and biomass-based energy sources.^22,25^

Many countries are setting ambitious renewable energy goals and implementing policies and incentives to promote the transition to a cleaner energy matrix.^25,26^ As of 2021, this has resulted in the implementation of approximately 2,000 climate-change laws and related policies across the globe, all of which are deeply related to the reduction of carbon-based energy matrix.^27^ Notably, the European Union, United Kingdom, Japan, and South Korea have set 2050 as a milestone for achieving their energy transition goals, while China has identified 2060 as its target year.^25,26^

Despite the worldwide movement toward cleaner energy sources, significant challenges remain. These challenges include high installation costs, insufficient distribution infrastructure, ongoing dependence on fossil fuels, and resistance from certain sectors of society. Additionally, many communities around the globe still lack access to reliable energy sources, and their energy demand plays a crucial role in their development.^22^

Among the arsenal of solutions for decarbonized energy, hydrogen gas is considered an essential player due to its clean combustion.^28^ Its demand is expected to grow by 700% by 2050, driven by global efforts to transition to a carbon-free energy landscape. Such a transition based on hydrogen would have several advantages, including the transferability of skills, jobs, infrastructure, assets, and business models from the already existing oil and gas industry sectors.^7^ Its adoption has even been considered indispensable for decarbonizing hard-to-abate industrial processes at scale, such as those involving steel and cement production.^29^

### 2.1 Hydrogen as an Energy Carrier

Molecular hydrogen (H_2_) was first discovered by the British scientist Robert Boyle in 1671 while conducting experiments with iron and sulfuric acid, referring to the gas evolved as an “inflammable solution of iron” (eq 5). Nearly a century later, in 1766, Henry Cavendish confirmed Boyle’s discovery and identified hydrogen as a distinct “element” but mistakenly believed that hydrogen gas was released from the metals rather than from the acid in his experiments. It was not until 1788, however, that Antoine Lavoisier recognized hydrogen’s nature and gave it its current name, derived from the Greek words “hydro” meaning water, and “genes” meaning forming, according to the product of its combustion.^7,8,30^

5 $${\mathrm{Fe}}_{\left(s\right)}{+H}_{2}{\mathrm{SO}}_{4(\mathrm{aq})}\to {{\mathrm{Fe}}^{2+}}_{\left(\mathrm{aq}\right)}{{{+\mathrm{SO}}_{4}}^{2-}}_{\left(\mathrm{aq}\right)}{{+H}_{2}}_{\left(g\right)}$$

Apart from naturally occurring hydrogen, hydrogen gas is not considered an energy source but rather an energy carrier or vector because, similarly to electricity, it is generated using other energy sources.^31,32^ Nevertheless, storing other forms of renewable energies in hydrogen molecules ensures a more stable energy supply, unlike the intermittent nature of solar and wind energy.^33^

Hydrogen gas is nontoxic and lighter than air. It has the highest mass-specific energy density among chemical fuels, with a lower heating value (LHV) and a higher heating value (HHV) of 120 MJ kg^–1^ and 142 MJ kg^–1^, respectively. For comparison, gasoline has much lower LHV and HHV values, in the range of 36.4–49.6 MJ kg^–1 30,34^. While hydrogen gas dissipates quickly when released, making it safer than other fuels in case of a spill, the primary safety concern is the potential for explosions if a leak goes undetected and the gas concentrates in a confined space. Another issue with its implementation as a fuel is its low volumetric energy density (10.7–12.7 MJ m^–3^) which poses challenges for storage and transportation.^34^

### 2.2 From Gray to Green: Classifying Hydrogen Production Methods

While hydrogen fuel is considered an environmentally friendly option, it can be a complicated resource to obtain. Furthermore, many current technologies are still connected to fossil energy sources or are in their early stages of development, and natural sources of hydrogen remain largely unexplored.^29,32,35^

There are three main, but not unique, methods of obtaining hydrogen gas: (1) those based on the thermochemical conversion of fossil fuels and other organic molecules, (2) those based on the water splitting (via electrolysis or photolysis), and (3) those based on biological processes (using fermentation or biophotolysis).^30,34,35^ All these processes can be enhanced by using synthetic catalysts, which will be discussed in this paper.

Hydrogen is typically classified using a color system that reflects the various energy sources employed in its production. While this color classification can serve as a useful reference, it is essential to approach it with care due to inconsistencies found in the literature.^35,36^ Furthermore, the criteria used to assess the carbon intensity associated with different production pathways may not always be clearly defined or thoroughly explained.^31,34,36^ Figure 2 provides a general overview of the color classifications for hydrogen.

**Figure 2:**
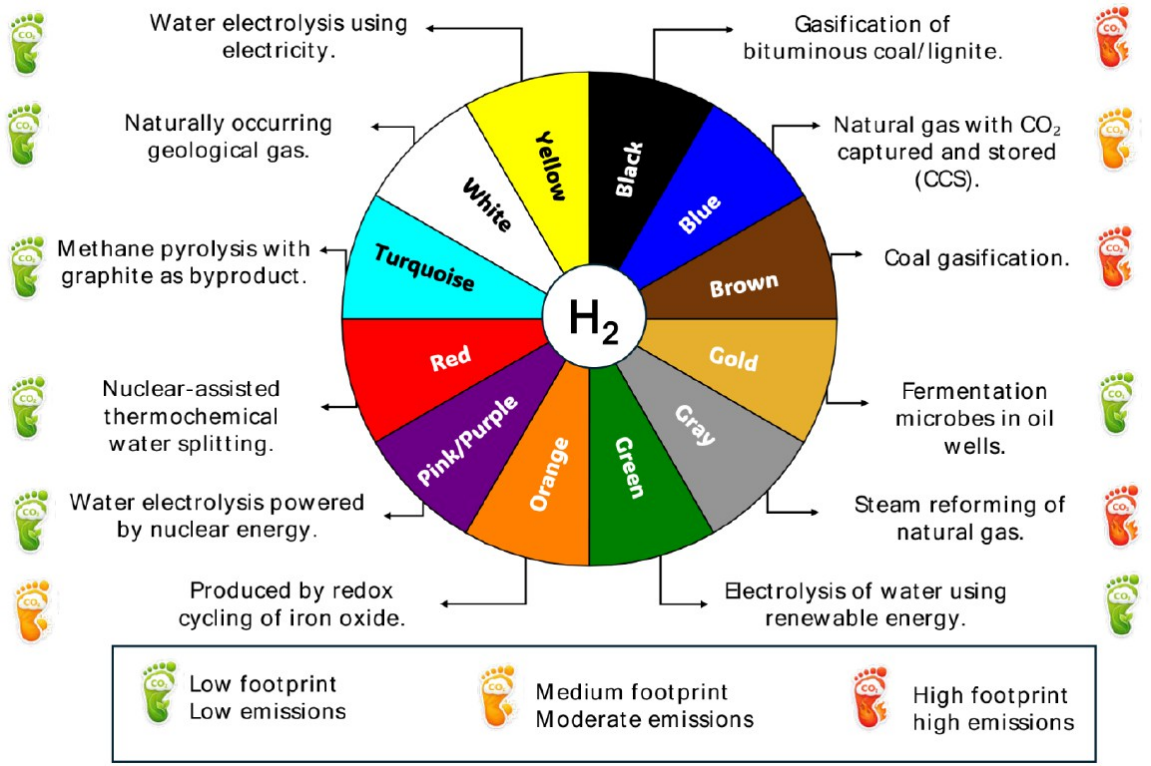
Hydrogen color classification according to means of production and CO_2_ footprint. Illustration by the authors based on the information provided by refs. 32,35,36 .

## 3 Catalysts for Hydrogen Production

### 3.1 Thermochemical Conversion of Fossil Fuels and Other Organic Molecules

#### 3.1.1 Steam Reforming (SR)

Steam reforming involves using organic molecules such as methane and ethanol to produce hydrogen.^37^ A large portion of global hydrogen production (71%) is obtained from natural gas steam reforming, with 27% coming from coal gasification.^8^ While effective, this method encounters challenges related to energy efficiency and carbon emissions, prompting increased research into renewable sources and catalysts that are more resistant to high temperatures.^34^

##### 3.1.1.1 Steam Methane Reforming (SMR)

The steam reforming of methane is the technology most widely used by industries to produce hydrogen.^38,39^ It was first reported in 1924 and introduced in the industry by 1930 [43]. This process involves the endothermic reaction between methane and water vapor at high temperatures (800–1100 °C) and pressures (14–40 bar), resulting in the formation of CO and H_2_ (eq 6). Subsequently, CO and water vapor may react to produce CO_2_ and H_2_ (eq 7).^40^

● Steam reforming (stricto sensu):

6 $${\mathrm{CH}}_{4}{+H}_{2}O⇌{\mathrm{CO}+3H}_{2}{,\mathrm{\Delta H}}_{298K}^{⊖}=+206{.\mathrm{3 kJmol}}^{\mathrm{-1}}$$

● Water shift reaction:

7 $${\mathrm{CO}+H}_{2}O⇌{\mathrm{CO}}_{2}{+H}_{2}{,\mathrm{\Delta H}}_{298K}^{⊖}{=\mathrm{-41 kJ mol}}^{\mathrm{-1}}$$

The unit operations for the SMR consist of three main parts—a feed unit, a fixed-bed reactor where the catalyst is inserted, and an analysis section (Figure 3). After exiting the reactor, the gases are cooled to eliminate any condensable molecules before passing through a drying unit to remove residual water. From there, the gases are directed to a detector to identify the presence of hydrogen. Once detected, additional separation and purification steps can be taken to eliminate impurities and obtain a high-quality end product.^41^

**Figure 3:**
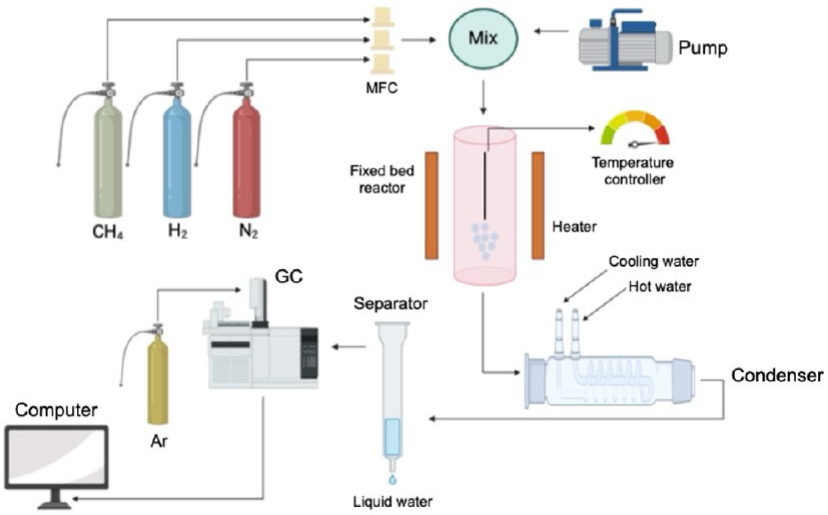
Diagram of operational units for methane steam reforming. Adapted from ref. 42. Copyright 2022, Elsevier. Illustration created using the BioRender.com platform.

Nickel-based catalysts are the most widely employed in industry for the Steam Methane Reforming (SMR) reaction. However, they are particularly susceptible to deactivation due to coke formation (carbon deposition resulting from secondary reactions), sintering, and poisoning by impurities like sulfur species. While research has shown that noble metal-based catalysts (e.g., Pd, Rh, Ir, Ru, Pt) exhibit high activity and durability, their elevated costs hinder their adoption in industrial applications.^43,44^

Table 1 displays some catalysts that have been recently investigated in methane reforming, all Ni-based. The results in the table are expressed in methane conversion (X_CH4_) and H_2_ yield (Y_H2_) according to eqs 8 and 9.

8 $$X_{\mathrm{CH4}}=\frac{F_{CH4in}-F_{CH4out}}{F_{CH4in}}\times 100$$

9 $$Y_{H2}=\frac{F_{H2}}{3\times F_{CH4}}\times 100$$

**Table 1:** Catalysts Studied in the Steam Methane Reforming (SMR)^a^

Catalyst	Mass/ (mg)	System conditions	Y_H2_/ (%)	Operational time/(h)	X_CH4_/ (%)	Temperature/ (°C)	Ref.
Ni_0.80_Co_0.20_-Zr	8000	S/C = 3, GHSV = 215 h^–1^	90	30	95	650	^38^
Ni-5.0 M 2C/FAU	55	S/C = 1.0	70	27	70	850	^39^
10Ni-10Co-1Ce/Al	1000	Atmospheric pressure; GHSV= 37,000 mL g _cat_^–1^.h^–1^.	98.8	12	100	700	^42^
NiAl_2_O_4_ spinel	100	F_CH4_/F_Ar_= 10 mL min^–1^; F _H2O(v)_ = 36.6 mL/min; GHSV= 12,349 h ^–1^ ; *P* = 0.1 MPa;	-	160	84.8	600	^45^
20Ni-1.5Ce/Al_2_O _3_	1000	Atmospheric pressure. GHSV= 37.000 mL gr ^–1^ h ^–1^.	92.2	-	85	700	^46^
Ni/CeO_2_ -BM**^b^	50	Atmospheric pressure. GHSV = 5400–41,580 h ^–1^; S/C = 2.	-	5	100	700	^47^
NiFe/Al_2_ O_3_:m(Al_2_ O_3_	100	GHSV = 42,000 mL g^–1^ h ^–1^	53	8.3	-	600	^48^
20Ni-3,0Y/HAl	1000	Atmospheric pressure; GHSV = 37,000 mL g _cat_^–1^.h^–1^.	97.74	12	95.7	700	^49^
Ni_2_ -Co_1_ /H–Al_2_O_3_	1000	Atmospheric pressure; S/C= 3; GHSV= 37,000 mL g^–1^ h ^–1^	92.7	12	100	700	^50^
0.05MgO–Ni/γ-Al_2_O _3_	-	Atmospheric pressure	55	10	75	600	^51^
Ni@Al_2_O_3_ yolk shell	100	S/C = 3; GHSV= 932.492 mL h^–1^ g^–1^	93	24	89	750	^52^
Ni–V/GDC	-	S/C = 2.6; GHSV= 10.000 h^–1^	-	100	96.9	800	^53^
3Sm–Ni/SBA-15	200	H_2_O/CH_4_ = 3; GHSV = 9 Lg_cat_^–1^ h^–1^; *P* = 1 atm	66	30	70	700	^54^

a 1S- Number of immersion cycles; 15%Ni-hydrocalumite.

b Ballmilling; S/C= Steam/Vapor ratio; GHSV, Gas Hourly Space Velocity; FAU-Zeolite.

, where F_H2_ and F_CH4_ represent the molar flux of H_2_ and CH_4_, respectively.

**Mechanistic Insights into Catalytic SMR**

As Figure 4 shows, the SMR entails a complex series of reactions that can be categorized into five pathways: CH_4_ dissociation, H_2_O dissociation, C oxidation, CH oxidation, and H_2_ formation. The chemical events for the pathways outlined in Figure 4 are detailed in Table 2. The predominant reactions for each system, nonetheless, will vary depending on the catalyst chosen and the reaction conditions.^55^

**Figure 4:**
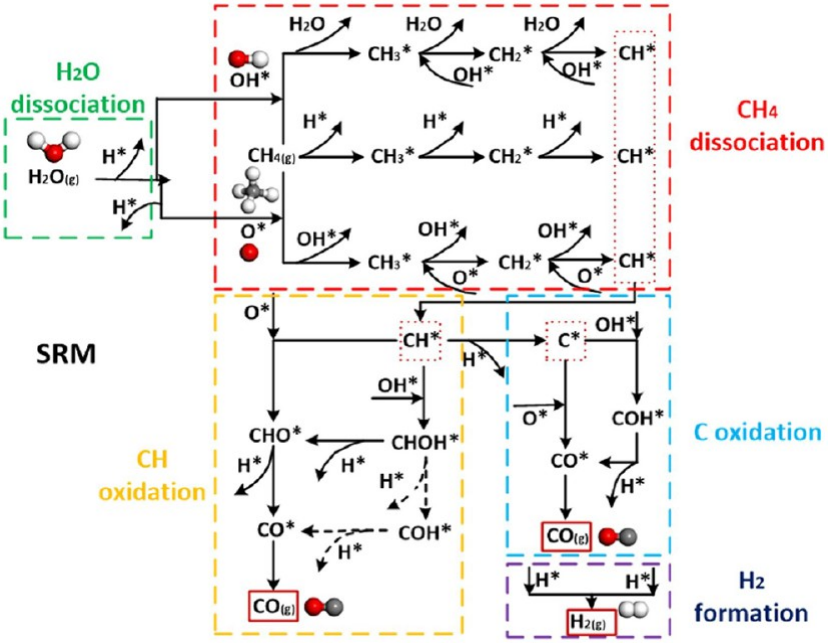
Reaction network for methane steam reforming: H_2_O dissociation (green rectangle), CH_4_ dissociation (red rectangle), CH oxidation (orange rectangle), C oxidation (light blue rectangle), and H_2_ formation (dark blue rectangle). Reprinted with permission from ref. 56. Copyright 2020, Elsevier.

**Table 2:** Reaction Pathways and Chemical Events in the SMR According to ref. 55

Reaction pathway	Chemical events
**H**_**2**_ **O dissociation**	The water dissociates into ●H and ●OH.
**CH**_**4**_ **dissociation**	Methane dissociation can occur due to ●OH-assisted activation, direct dissociation, and ●O-assisted dissociation.
**C oxidation**	C can either deposit over the catalyst surface or oxidize to CO and CO_2_, depending on the concentration of oxygenated species in the reaction medium.
**CH oxidation**	The ●CH radical produced during the dissociation of CH_4_ can undergo various oxidation pathways. It can be oxidized to ●CHO by O_2_ or to ●CHOH by ●OH, and both are precursors for CO and ●H generation. The ●CH radical may also undergo reduction to produce C and ●H.
**H**_**2**_ **formation**	The ●H species formed during other pathways combine to produce H_2_.

The rate-limiting step in SMR is believed to be the dissociative adsorption of CH_4_, which involves the homolytic breaking of the σ_C–H_ bond of CH_4_ on a metal surface. Activation of CH_4_ generally comprises a group of active metal centers rather than just a single active metal atom, with the σ_C–H_ bond cleavage occurring preferentially at coordination-unsaturated sites.^43^

Recent kinetic studies conducted between 2024 and 2025 have corroborated that the rate-limiting step involves the dissociative adsorption of methane (CH_4_ → CH_3_* + H*) on the metal surface.^57,58^ Notably, cobalt (Co) doping has been shown to shift the rate-determining step to the dissociative dehydrogenation of CH*, illustrating the potential for modulating the reaction kinetics.^59^

On supports such as CeO_2_, water dissociation at the metal–support interface occurs with minimal barriers, producing essential hydroxyl species (OH*) that oxidize surface carbon (CH_x_*) to carbon monoxide (CO), thus preventing coke formation. This “hydroxyl-assisted” pathway shows a significantly lower energy barrier compared to those relying solely on lattice oxygen.^60^ Seong et al. (2025) highlighted the vital role of oxygen vacancy sites in facilitating methane conversion and steam gasification reactions. They sought to tackle the challenges associated with high operating temperatures by investigating the low-temperature Chemical Looping Steam Methane Reforming (CL-SMR) process using c-CeO2 as a carbon carrier. However, to ensure the technology remains competitive, further optimization will be necessary.^61^

The electronic state of the metal surface or d-band plays a crucial role in the catalyst’s activity. In certain alloy systems, the d-band center can serve as a parameter for predicting the trend of activation energy for CH_4_ dissociation. An increase in the d-band center is expected to correspond to higher activity, whereas a decrease in the d-band center is likely to result in lower activity.^43^

**Addressing SMR challenges**

The SMR presents several challenges, such as high energy consumption, CO_2_ emissions, low reaction efficiency, process instability, and coking, ultimately leading to catalyst deactivation.^62,63^ High temperatures also promote catalyst sintering, reducing the catalyst’s lifespan.^40,64^ The following two sections will provide literature examples that address the challenges related to carbon emissions and catalyst instability, focusing on the use of appropriate catalysts to mitigate these issues.

**Toward Low-Carbon SMR: Strategies for CO_2_ Capture**

One approach to deal with the CO_2_ emissions from SMR is the sorption-enhanced steam methane reforming (SESMR) technology. This technology combines *in situ* CO_2_ capture with the traditional SMR process (eqs 10 and 11), shifting the equilibrium toward the production of more hydrogen while CO_2_ is being captured.^45,65^ Additionally, even after accounting for the energy required to regenerate the sorbent, this technology is still 20% less energy-intensive than SMR followed by downstream purification.^66^

● CO_2_ capture

10 $${\mathrm{CO}}_{2}+\mathrm{CaO}⇌{\mathrm{CaCO}}_{3}\Delta H_{298K}^{{}^{\circ}}{=-178\mathrm{kJ}\mathrm{mol}}^{-1}$$

● Overall equation for the sorption-enhanced steam methane reforming

$${\mathrm{CH}}_{4}{+H}_{2}O+\mathrm{CaO}⇌{\mathrm{CaCO}}_{3}{+4H}_{2}\Delta H_{298K}^{{}^{\circ}}{=-13\mathrm{kJ}\mathrm{mol}}^{-1}$$ 11

In their 2022 study, Ayesha et al.^66^ developed a CaO@Mg–Ni–Al (hydrocalcite) composite for the SESMR reaction. A key feature of their research was using CaO extracted from eggshells as the CO_2_ sorbent, effectively combining carbon capture with waste recycling (Figure 5). Different CaO loadings, ranging from 0% to 15%, were incorporated into the Mg–Ni–Al-based catalyst using a fixed bed reactor. The optimal performance was observed with 10% CaO loading, which extended the prebreakthrough period of the reaction—the period before the hydrogen production starts to decrease due to the sorbent saturation by CO_2_. Utilizing CaO raised the hydrogen purity from 55% to 80% while reducing CO_2_ production from 14% to just 3%.

**Figure 5:**
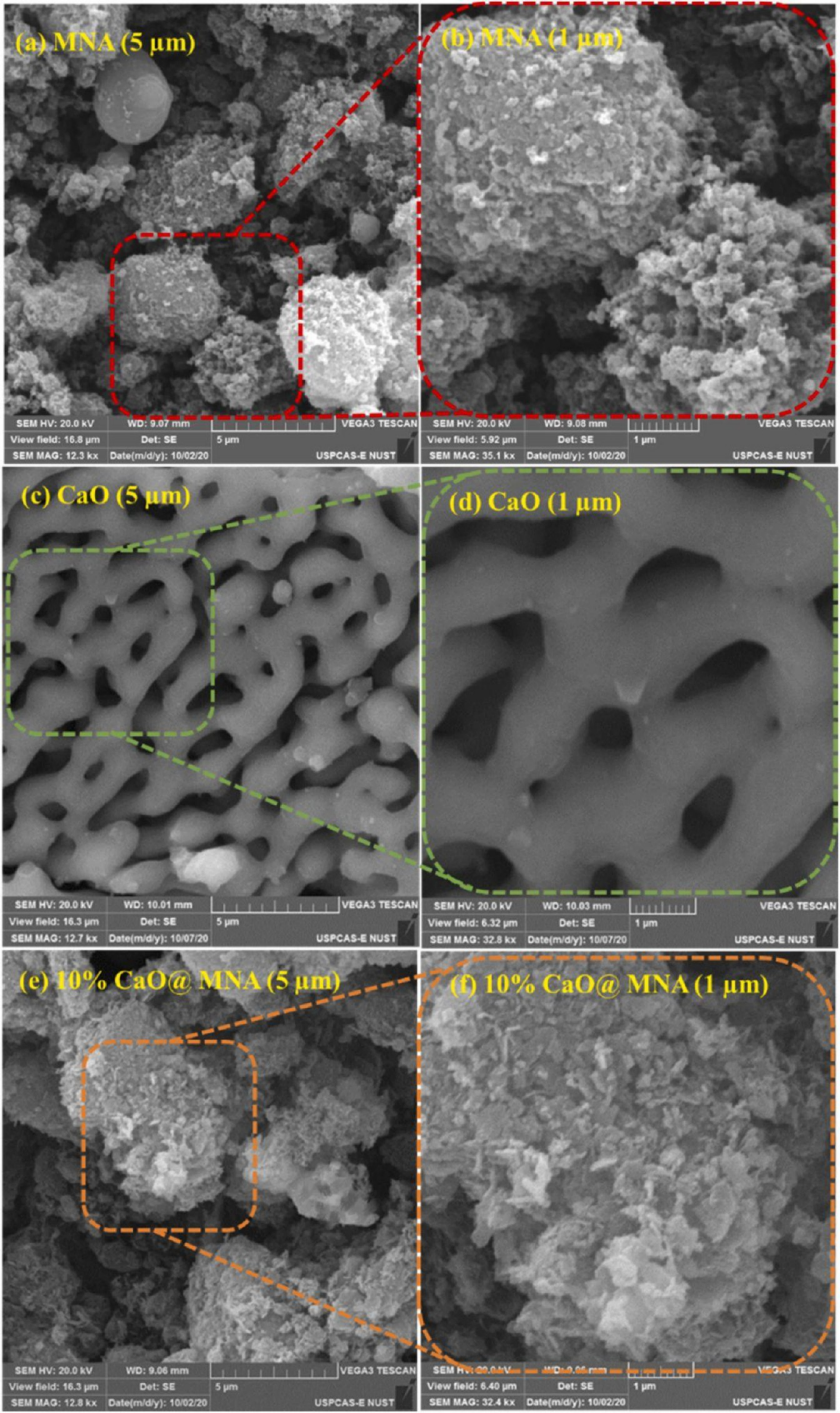
Scanning electron micrographs of synthesized catalyst at 5 and 1 μm (a–b) Mg–Ni–Al catalyst (c–d) CaO, (e–f) CaO@Mg–Ni–Al. Reprinted with permission from ref. 66. Copyright 2022, from Elsevier.

Likewise, in the study conducted by Wang et al.,^45^ a system using alumina-supported catalysts was introduced for the SESMR reaction. The catalysts comprised three Ni-based bimetallic alloys (Ni_3_M/Al_2_O_3_, with M = Cu, Fe, and Ge). Incorporating limestone (calcium oxide) as a CO_2_ sorbent, following a prior mixing with the catalysts and calcination, led to significant improvements in methane conversion, CO selectivity, hydrogen yield, and hydrogen purity. When compared to the SMR reaction at the same reaction temperature (T = 700 °C), the addition of limestone resulted in an approximately 20–30% increase in methane conversion for all catalysts, with Ni/Al_2_O_3_ and Ni_3_Cu/Al_2_O_3_ achieving a methane conversion rate of 95%, approaching the thermodynamic equilibrium. Furthermore, hydrogen yield (ca. 85%) and purity (ca. 80%) increased by 30% and 20%, respectively. The addition of Cu was found to enhance the surface area and metal dispersion, thereby improving the overall morphology of the catalyst.

**Understanding Catalyst Deactivation in SM**

**Protecting SMR Catalysts from Sulfur Contamination**

The deactivation of Ni-based reformer catalysts by hydrogen sulfide, even at ppb levels, is well-documented in the literature. Besides carbon fouling (discussed later in the next section), sulfur poisoning presents the highest risk to catalyst deactivation in SMR. This is attributed to the chemisorption of sulfur on the catalyst surface (eq 12), which can also alter the electronic distribution of the nearby atoms.^67–71^ According to simulations conducted by Sadooghi and Rauch,^68^ consistent with the results found for an operating plant, the presence of sulfur in the feedstock, even at ppm levels, could significantly impact methane conversion, hydrogen yield, and even the temperature distribution in the reactor with a nickel-based catalyst.

● Chemisorption of H_2_S on the metal surface (M)

12 $$H_{2}S+M⇌{\mathrm{M-S}+H}_{2}{\;\Delta G^{{}^{\circ}}}_{1000K}\mathrm{∼-100}{{\mathrm{kJ mol}}_{S2}}^{\mathrm{-1}},\mathrm{if M}=\mathrm{Ni}$$

Capa et al.^72^ have synthesized a Pd/Ni–Co hydrotalcite-like material (HT) catalyst using dolomite (CaMg­(CO_3_)_2_) as a CO_2_ sorbent to perform an SESMR reaction. The carbon feedstock consisted of biogas, which contained 60% CH_4_ (v/v)—and explains why their work is considered here—the rest corresponding to CO_2_. According to the authors, biogas is known for containing H_2_S as an impurity, which makes a sulfur-resistant catalyst particularly appealing for such a feedstock. Also, according to them, the addition of Co in Ni-based catalysts alters the sulfur chemisorption kinetics, delaying the deactivation, while the addition of Pd, a noble metal, makes the deactivation even more unlikely at lower concentrations of H_2_S. They evaluated different concentrations of H_2_S (150, 350, 500, and 1000 ppm) and found that the catalyst did not deactivate for H_2_S concentrations of 150 and 350 ppm during five cycles of the SESMR process. Nonetheless, they noticed a slight decrease in the catalyst activity under higher sulfur concentrations after the third SESMR cycle. As shown in Figure 6, which presents the sulfur contents in the gas phase and the solid phase (catalyst + dolomite), the CO_2_ sorbent also adsorbs H_2_S. The authors also claim that besides the role performed by the sorbent, the addition of Co to the catalyst contributed to the resistance of the catalyst when compared with other reported results in the literature.

**Figure 6:**
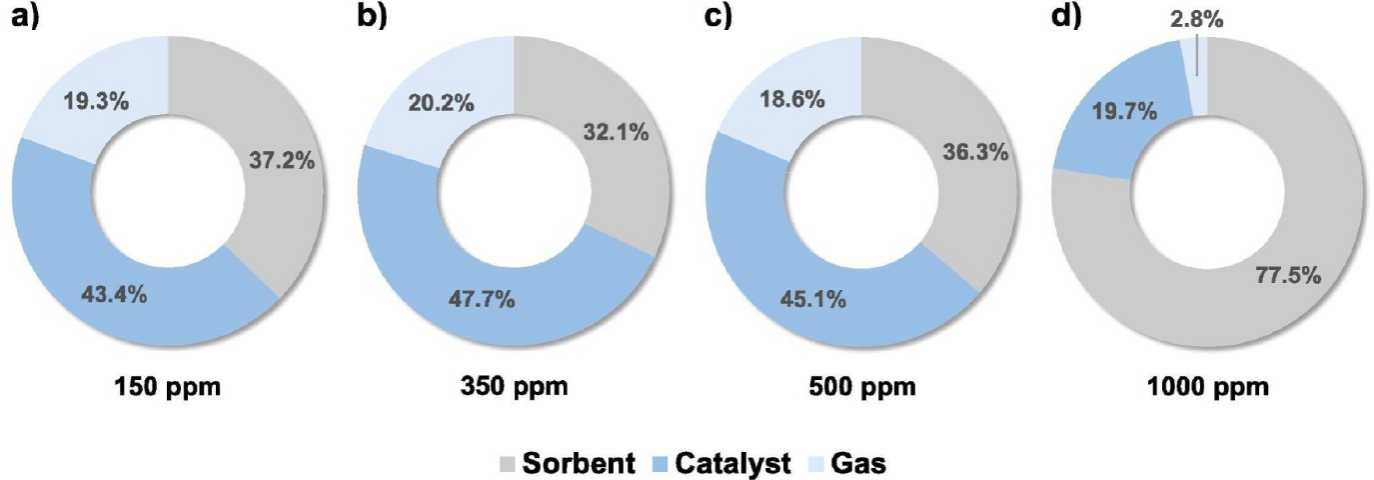
Sulfur distribution (wt %) between sorbent, catalyst, and evolved gas after five cycles of the SESR process for biogas H_2_S concentrations of 150 (a), 350 (b), 500 (c), and 1000 ppm (d). Operating conditions: Biogas = 60/40 CH_4_/CO_2_ vol %; *T* = 600 °C; S/CH_4_ = 6 mol/mol; GHSV = 1803 mL_CH4_ g_cat_^–1^ h^–1^; sorbent/catalyst ratio = 20 g/g; Pd/Ni–Co HT catalyst and dolomite sorbent. Reprinted with permission from ref. 72. Copyright 2023, Elsevier.

**The Challenge of Coking in SMR**

During the transformation of organic compounds using solid catalysts, the production and retention of secondary products often occur due to byproducts, as in eqs 13–15. These products, which remain on the catalyst and consist mainly of solid carbon, are commonly known as coke.^73–76^ By deactivating the catalyst, the presence of coke blocks the catalyst’s active sites, reducing its ability to promote the desired reactions.^77^ This leads to an increase in operating costs, due to the need for frequent regeneration or replacement of the catalyst.^78^

13 $${\mathrm{CH}}_{4}\to {C+H}_{2}$$

14 $${\mathrm{CO}+H}_{2}⇌{C+H}_{2}O$$

15 $$\mathrm{2CO}\to {C+\mathrm{CO}}_{2}$$

The stability of nickel-based catalysts in reforming reactions is fundamentally determined by the interplay between carbon deposition (coking) and oxidative removal. A comparative analysis of recent studies indicates that the support material serves not only as a structural framework, but also as an active chemical participant that influences reaction pathways and overall stability. The key factor distinguishing stable systems from those that deactivate appears to be the capability of the metal–support interface to enable low-barrier oxidative pathways. For example, Salcedo et al. demonstrated that in Methane Steam Reforming (SMR) with Ni/CeO_2_, stability is driven by a specific associative mechanism wherein the support facilitates water dissociation. This interaction promotes the barrierless formation of hydroxyl (OH) groups at the interface, allowing the catalyst to engage carbon with OH, generating a COH intermediate. This pathway requires an activation energy of only 0.89 eV, substantially lower than the barrier for direct carbon oxidation using lattice Oxygen, thereby effectively circumventing the thermodynamic sink that leads to carbon accumulation.^60^

In contrast, when the support is unable to provide sufficient oxidative species to keep pace with the rate of carbon formation, physical degradation occurs, as demonstrated by Liu et al. in their study on Ethanol Steam Reforming (ESR).^79^ While Salcedo et al.^60^ emphasized the chemical prevention of coke formation through the use of redox-active supports such as CeO_2_, Liu et al. quantified the structural impacts of coking on nonreducible, basic supports (nickel-hydrocalumite). Despite the high surface area and basicity of hydrocalumite, which initially enhances activity, the system ultimately experienced significant physical blockage over time. Specifically, Liu et al. recorded a dramatic 99.8% reduction in porosity (from 0.971 to 0.00159) within just 24 h, along with a structural evolution of the carbon deposits characterized by a decline in the degree of graphitization (with the IG/ID ratio falling from 4.1 to 0.7).^79^

The integration of these findings indicates a unifying design principle for scalable, coke-resistant catalysts: the stability of these catalysts is less reliant on maximizing surface area, as exemplified by the hydrocalumite case, and more dependent on optimizing the metal–support interface to reduce energy barriers for oxidant activation, as demonstrated in the ceria case. Future research should therefore explore whether the “oxygen reservoir” capability of reducible oxides can be effectively combined with the high surface area of basic supports, thereby uniting the advantages of both material classes.^60,79^

##### 3.1.1.2 Ethanol Steam Reforming (ESR)

To mitigate the drawbacks of using fossil fuels for hydrogen production, ethanol steam reforming (ESR) has emerged as an eco-friendly alternative. ESR operates at 300 to 800 °C. Although these temperatures are still elevated, they are milder than those required for steam methane reforming (SMR). When coupled with in situ CO_2_ separation, ESR can achieve an impressive thermal efficiency of 86%.^80,81^ The unit operations of ESR resemble those of methane steam reforming, as illustrated in Figure 3.^43^

ESR involves a wide variety of reactions that can occur sequentially or simultaneously, as described in eqs 16–26:^45^

● General equation

16 $${\mathrm{CH}}_{3}{\mathrm{CH}}_{2}{\mathrm{OH}+H}_{2}O\to {4H}_{2}+\mathrm{2CO}$$

● Ethanol dehydrogenation

17 $${\mathrm{CH}}_{3}{\mathrm{CH}}_{2}\mathrm{OH}\to {\mathrm{CH}}_{3}{\mathrm{CHO}+H}_{2}$$

● Ethanol dehydration

18 $${\mathrm{CH}}_{3}{\mathrm{CH}}_{2}\mathrm{OH}\to C_{2}H_{4}{+H}_{2}O$$

● Ethanol decomposition

19 $${\mathrm{CH}}_{3}{\mathrm{CH}}_{2}\mathrm{OH}\to {\mathrm{CH}}_{4}{+\mathrm{CO}+H}_{2}$$

● Acetaldehyde steam reforming

20 $${\mathrm{CH}}_{3}{\mathrm{CHO}+H}_{2}O\to {3H}_{2}+\mathrm{2CO}$$

● Acetaldehyde decomposition

21 $${\mathrm{CH}}_{3}\mathrm{CHO}\to {\mathrm{CH}}_{4}+\mathrm{CO}$$

● Ethylene steam reforming

22 $$C_{2}H_{4}{+2H}_{2}O\to {\mathrm{2CO}+4H}_{2}$$

● Ethylene decomposition

23 $$C_{2}H_{4}\to {2C+2H}_{2}$$

● Steam methane reforming (SMR)

24 $${\mathrm{CH}}_{4}{+H}_{2}O⇌{\mathrm{CO}+3H}_{2}$$

● Gas shift reaction (WGS)

25 $${\mathrm{CO}+H}_{2}O⇌{\mathrm{CO}}_{2}{+H}_{2}$$

● Boudouard reaction

26 $$\mathrm{2CO}⇌{\mathrm{CO}}_{2}+C$$

The reaction pathway in ESR is predominantly influenced by the acid–base characteristics of the support material. The process diverges into two competing routes:

1. Dehydration (the “coking” route): This pathway, catalyzed by acidic sites such as γ-Al_2_O_3_, involves the dehydration of ethanol to produce ethylene (C_2_H_4_) or C_2_ intermediates. These intermediates serve as precursors for polymerization, which results in the formation of amorphous coke on the acidic support sites, rather than graphitic coke.^82^

2. Dehydrogenation (the “clean” route): This route, facilitated by basic sites like La_2_O_3_ and MgO, or neutral supports modified to reduce acidity, converts ethanol into acetaldehyde (CH_3_CHO).^83,84^ Ideally, the process progresses with C–C bond cleavage, leading to the generation of methane (CH_4_) and carbon monoxide (CO). However, the decomposition of methane can result in the formation of filamentous (graphitic) coke on the metal surface.^82^

Recent research has highlighted specific modes of deactivation associated with acetaldehyde. Rather than decomposing, acetaldehyde can undergo aldol condensation on the catalyst surface, producing long-chain carbon species. This pathway contributes to the accumulation of carbonaceous species that are distinct from the filamentous carbon resulting from methane cracking.^82,85^ While modifying support properties is vital for enhancing stability, a 2024 study on Ni/vermiculite catalysts illustrated that acidic treatment of the support significantly improved metal reducibility and reduced carbon deposition, thus outperforming alkali treatment in stability assessments.^86^

Simultaneous or sequential reactions can hinder selectivity, particularly in the presence of C–O bonds, leading to reduced efficiency and potential coke genesis during hydrogen formation.^87^ Therefore, catalysts that enhance both reaction efficiency and selectivity are crucial.

**Catalysts used in ESR**

Transition metal-based catalysts have been extensively investigated for H_2_ production via ESR. Optimizing their physicochemical properties, governed by chemical composition and synthesis conditions, is essential to maximize the H_2_ generation rate. Among the most commonly employed active phases are non-noble metals such as Ni, Co, and Cu, as well as noble metals including Pt, Pd, Rh, and Ru. Although noble metals generally exhibit superior catalytic activity, their high cost has driven research toward more economically viable alternatives.^88,89^

In this context, Ni and Co stand out for their catalytic performance. Ni is widely used due to its high availability, low cost, and strong ability to promote C–C and C–H bond cleavage, as well as to facilitate the recombination of hydrogen atoms into H_2_. Co may exhibit bond-cleavage activity comparable to that of Ni; however, it is generally more susceptible to rapid coke deposition, which compromises its stability and catalytic performance over time.^88,89^

Given these limitations, particularly for base transition metals, the appropriate choice of support material becomes a determining factor in optimizing catalytic performance.^90^ Although the support does not act directly as the active phase, it plays a fundamental role in modulating the activity, selectivity, and stability of the catalyst, thereby influencing reforming reactions.^88^

Reducible oxides, such as CeO_2_, tend to exhibit weaker metal–support interactions, whereas irreducible oxides, such as Al_2_O_3_, promote stronger interactions, resulting in enhanced catalytic activity and improved resistance to sintering.^88,91^ In this context, Table 3 summarizes representative catalysts that have been recently investigated for ethanol reforming. The catalytic performance is reported in terms of ethanol conversion (X_EtOH_) and H_2_ yield (Y_H2_). These parameters have been calculated differently in various articles; here, in eqs 27 and 28 will be considered.

27 $$X_{\mathrm{EtOH}}=\frac{\left(nEtOHin-nEtOHout\right)}{nEtOHout}\times 100$$

28 $$Y_{H2}=\frac{\left(nH2out\right)}{6.nC2H5OHin}\times 100$$

**Table 3:** Catalysts Utilized in the Ethanol Reform Reaction

Catalyst	System conditions	Y_H2_/(%)	Operational time/(h)	X_EtOH_/(%)	Temperature/(°C)	Ref.
Ni-CaO-CaZrO_3_	1 atm; WHSV= 0.34 h ^–1^; S/C = 3. 3000 mg of catalyst.	90	3	-	600	^45^
8Co/MgAl_2_O_4_	W_cat_/F _EtOH_ = 114.7 g cat min g _EtOH_^–1^; H_2_O/EtOH = 3; P EtOH = 2.75 kPa. 120 mg of catalyst.	70	-	100	450	^62^
(15Ni–HCa)*	Atmospheric pressure; C_2_H_5_OH:H_2_O= 1:6. 200–400 mg of catalyst	86	-	99	650	^79^
15Ni7.5Mg/HCa	n _água_:n _etanol_ = 6:1; GHSV = 10,000 h ^–1^; total flow rate = 120 mL/min. 300–500 mg of catalyst.	90	-	100	650	^80^
Ir–Fe/Al_2_O	C_2_H_5_OH:H_2_O= 1:3; GHSV = 88,000 mL g ^–1^ h ^–1^. 100 mg of catalyst.	-	-	100	400	^81^
NiO-CuO-CaO-Ca_12_ Al_14_ O_33_	Atmospheric pressure; GSHV= 1,500 L/g _cat_ ·h; S/C = 1.5	87	-	100	500	^92^
9Ni1Co/CeMgAl	Atmospheric pressure; S/C = 3; LHSV = 10 mL/(g cat ·h). 300 mg of catalyst.	70	100	93.9	600	^93^
10 wt %Ni-5 wt %Co/Al_2_O_3_	Atmospheric pressure; S/C = 12; LHSV = 0.03–0.05 L/g _cat_.h. 500 mg of catalyst.	95.14	50	100	600	^94^
Co | Z (750)	Ethanol/H_2_O = 1:12; WHSV= 52 h ^–1^	95	160	100	500	^95^
NiMo/SBA-15	Atmospheric pressure; S/C = 2 WHSV = 156 h ^–1^. 50 mg of catalyst.	54	65	89	600	^96^
Ni-CeLa_0.20_	Atmospheric pressure; GHSV= 55.920 mL/g _*cat*_ ·h; H_2_O/etanol= 4/1. 250 mg of catalyst.	-	50	100	600	^97^
Pt–Cu@mSiO _2_	Atm. pressure (S/C = 4, WHSV = 8.5 h ^–1^). 500 mg of catalyst.	70.88	150	100	400	^98^
Ce_90_Ga_10_Ox	(H_2_O:C_2_H_5_OH = 6:1)	40	-	97	500	^99^
10Ni/MTC-72h	Atm. pressure. 300 mg of catalyst.	95.6	100	100	550	^100^

, where F_H2_ and F_EtOH_ represent the molar flux of H_2_ and C_2_H_5_O_2_, respectively.

#### 3.1.2 Dry Reforming

##### 3.1.2.1 Catalytic Decomposition of Methane (CDM): Toward Cleaner Hydrogen

The catalytic decomposition of methane (CDM) is emerging as a promising alternative to methane steam reforming. This process not only generates hydrogen gas but also yields solid carbon, which has potential commercial applications.^101,102^ Traditionally, methane decomposition occurs at temperatures exceeding 1300 °C, leading to product formation as outlined in eq 29. However, the requirement for such high decomposition temperatures has spurred research into the use of catalysts to enhance the industrial viability of the method.^101–103^

29 $${\mathrm{CH}}_{4(g)}\to C_{\left(c\right)}{+2H}_{2(g)},\Delta H_{298K}=74{.\mathrm{52 kJ mol}}^{\mathrm{-1}}$$

The reaction process takes place in a fixed-bed system consisting of three distinct parts: a feed unit, a methane decomposition reactor, and an analysis section (Figure 7).

**Figure 7:**
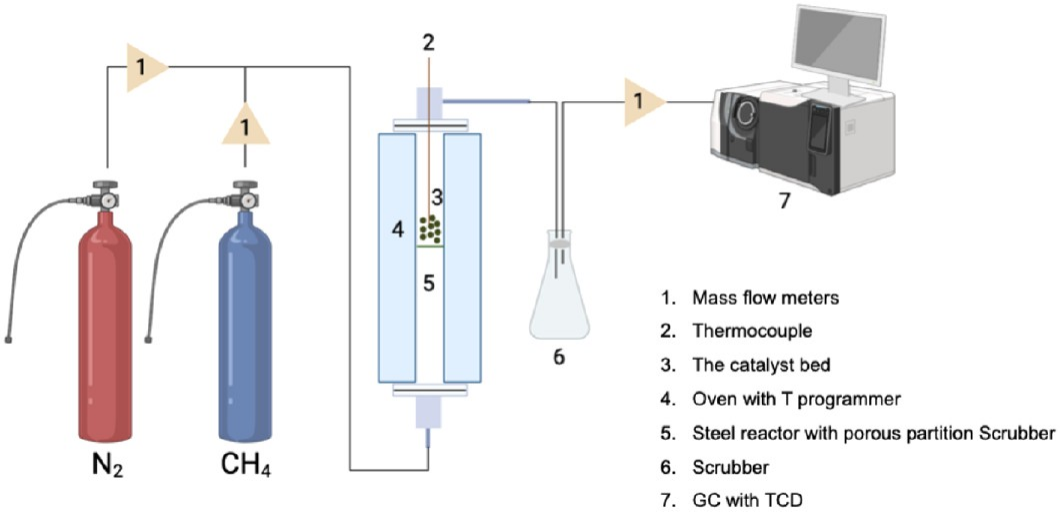
Representation of the methane decomposition process. Reprinted (adapted) with permission from ref. 104. Copyright 2023, Elsevier. Illustration created using the BioRender.com platform.

Transition metals serve as interesting catalysts in CDM due to their partially filled 3d orbitals, which can accept electrons from hydrocarbon molecules, facilitating the breakdown of these organic compounds.^105^ Table 4 presents various studies utilizing different catalysts to produce hydrogen through the catalytic decomposition of methane. The table displays the results in terms of methane conversion (X_CH4_) and H_2_ yield (Y_H2_) based on eqs 30 and 31.

30 $$X_{\mathrm{CH4}}=\frac{CH4in-CH4out}{CH4in}\times 100$$

31 $$Y_{H2}=\frac{H2out}{2\times CH4in}\times 100$$

**Table 4:** Catalyst Utilized in the Catalytic Decomposition of Methane^a^

Catalyst	System conditions	Y_H2_/(%)	Operational time/(h)	X_CH4_/(%)	Temp./(°C)	Ref.
Fe/Al_2_O_3_	CH_4_: N_2=_ 1:4. Atm. pressure	82.3	3	-	650–700	^104^
Fe/CTN	CH_4_: N_2=_1:4. Atm. pressure. Ten g of catalyst.	75–85	40	-	700	^106^
Fe–Al	CH_4_ (75%) e N _2_ (25%) Atm. pressure. 0.5 g of catalyst. 176.10 × 10 ^–5^ mol_H2_ g^–1^ min^–1^		5	5	800	^107^
1.5P/CEP950	(WHSV) foi de 1500 mL/(h. g_cat_), Atm. pressure. 0.2 g of catalyst.	-	8.3	6	800	^108^
ATNF	space velocity of 1.35 L/(h·g_cat_). Two g of catalyst.	-	60	2	850	^109^
Ni–Mn–Ru/Al_2_O_3_	GSHV = 36,000mL/g_cat_ h. 0.05 g of catalyst.	-	1	3.76	750	^110^
Co/CeO _2_ -BFA	CH_4_ flow rate = 20mL min^–1^. 0.5 g of catalyst.	44.9	34	71	850	^111^

a CTN, carbon nanotubes; ATNF, acid-thermal-treated nickel foam; BFA, biomass fly ash.

Nickel, iron, and cobalt are especially effective and are considered the most suitable metals for generating hydrogen through methane decomposition.^107,109–111^ Nickel catalysts exhibit the highest activity, but lose stability at temperatures above 650 °C. In contrast, iron and cobalt catalysts, although less active, can withstand higher temperatures and produce carbon materials with higher commercial value.^105^

Yang et al.^104^ investigated various iron contents supported on Al_2_O_3_ to decompose methane into hydrogen, identifying 12% iron as optimal. This dose yielded an impressive 82.25% conversion over 3 h at 700 °C. The authors noted that higher iron contents adversely affect metal dispersion, decreasing active surface area. In contrast, Gao et al. employed Ni/Al_2_O_3_ as a catalyst, achieving a methane conversion rate of 79.2% at 650 °C within 1.1 h, which suggested that iron outperformed nickel in this application. Furthermore, Gao et al. evaluated Co/Al_2_O_3_ and found a conversion rate of 64.7%, which was lower than that of Ni/Al_2_O_3_. The authors attributed this reduced performance to the agglomeration of metal particles in the cobalt catalyst.^112^

Abdel-Fattah et al.^107^ analyzed the performance of iron and cobalt (Fe–Al and Co–Al), without supports, in the catalytic decomposition of methane. With the Fe–Al catalyst, they observed a methane conversion of 95% and a hydrogen formation rate of 176.10 × 10^–5^ mol H_2_ g^–^1^^ min^–^1^^, with no signs of deactivation over 300 min. In contrast, the Co–Al catalyst showed a conversion of 66.4% and a hydrogen formation rate of 134.50 × 10^–5^ mol H_2_ g^–^1^^ min^–^1^^, but suffered deactivation after only 30 min of reaction. According to the authors, the larger surface area and nanoflake morphology of the Fe–Al catalyst may have favored its stability and catalytic efficiency. Co–Al, on the other hand, with a structure covered with fine agglomerates, probably had its deactivation accelerated by the increase in agglomeration during the reaction.

### 3.2 Catalytic Water Splitting: Electrochemical and Photochemical Routes

#### 3.2.1 Electrocatalytic Water Splitting: Harnessing Electricity for Hydrogen Production

Water electrolysis is regarded as one of the most promising methods for achieving carbon neutrality, as it can generate high-purity hydrogen without producing pollutants or harmful byproducts.^113,114^ However, the process is significantly hindered by its high energy consumption.^115–117^ To address this challenge, efforts have been made to harness renewable energy sources such as solar, wind, geothermal, tidal, and nuclear power.^117,118^

##### 3.2.1.1 Understanding the Electrochemical Basis of Water Splitting

Aqueous electrolysis involves nonspontaneous oxidation–reduction reactions in which water molecules are decomposed into hydrogen and oxygen gases at their respective electrodes when an electric current is applied to the system.^119,120^ The overall reaction for water splitting is expressed in eq 32.^121^

Global reaction:

32 $$H_{2}O_{\left(l\right)}\to H_{2\left(g\right)}+\frac{1}{2}O_{2\left(g\right)}\left(E_{\mathrm{rev}}=1,229V\right)$$

$${\mathrm{\Delta G}}^{0}=+237,{\mathrm{1 kJ mol}}^{\mathrm{-1}},{\mathrm{\Delta H}}^{0}=+285,{\mathrm{8 kJ mol}}^{\mathrm{-1}}$$

$${Q=+48,\mathrm{7 kJ mol}}^{\mathrm{-1}}\left(298.\mathrm{15 K},\;\mathrm{1 atm}\right)$$

The water-splitting process consists of an oxygen gas evolution reaction (OER) at the anode, and a hydrogen gas evolution reaction (HER) at the cathode.^120^ The oxidation–reduction half-reactions are influenced by factors such as temperature, current density, pressure, the type of electrolyte used in solution and the pH of the medium (eqs 33–38; Figure 8). This technique is classified as low-temperature electrolysis when it occurs at intervals below 100 °C and high-temperature electrolysis above this value.^113^

**Figure 8:**
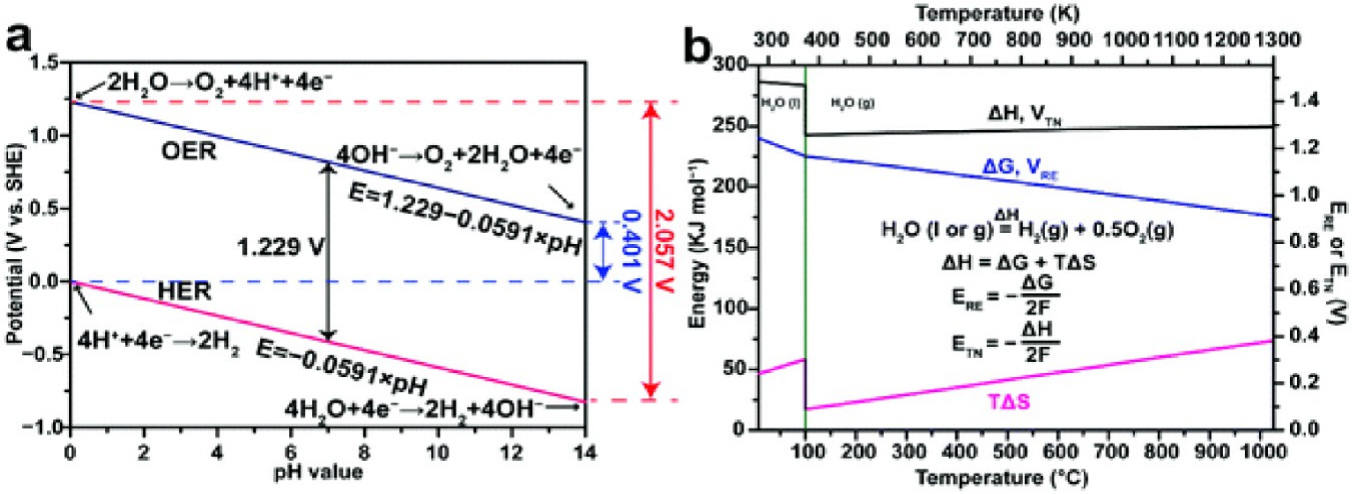
(a) Variation of the thermodynamic potentials of the hydrogen evolution reaction and the oxygen evolution reaction in aqueous electrolytes with different pH values. (b) Variation of the potentials and energy of the system as a function of temperature in aqueous electrolysis. (−) Variation of the reversible thermodynamic tension and Gibbs free energy; (−) variation of the thermoneutral tension and enthalpy of the system. (−) Heat of the system as a function of entropy. Reprinted with permission from ref. 121. Copyright Creative Commons License 2022, Royal Society of Chemistry.

● Acid electrolytes (pH < 6):

33 $$\mathrm{Cathode:}\;4H_{\left(aq\right)}^{+}+4e^{-}\to 2H_{2\left(g\right)}\left(E^{0}=1,229V,\;\mathrm{SHE}\right)$$

34 $$\mathrm{Anode:}\;2H_{2}O_{\left(l\right)}\to 4e^{-}+4H_{\left(aq\right)}^{+}+O_{2\left(g\right)}{(E}^{0}=0,00V,\;\mathrm{SHE})$$

● Basic electrolytes (pH > 7)

35 $$\mathrm{Cathode:}\;4H_{2}O_{\left(l\right)}+4e^{-}\to 2H_{2\left(g\right)}+4OH_{\left(aq\right)}^{-}\left(E^{0}=\mathrm{-0},828V,\;\mathrm{SHE}\right)$$

36 $$\mathrm{Anode:}\;4OH_{\left(aq\right)}^{-}\to O_{2\left(g\right)}+2H_{2}O_{\left(l\right)}+4e^{-}\left(E^{0}=0,\mathrm{414 V},\;\mathrm{SHE}\right)$$

● Neutral electrolytes (pH = 7)

37 $$\mathrm{Cathode:}\;4H_{2}O_{\left(l\right)}+4e^{-}\to 2H_{2\left(g\right)}+4OH_{\left(aq\right)}^{-}\left(E^{0}=\mathrm{-0},414V,\;\mathrm{SHE}\right)$$

38 $$\mathrm{Anode}\;:2H_{2}O_{\left(l\right)}\to 4e^{-}+4H_{\left(aq\right)}^{+}+O_{2\left(g\right)}\left(E^{0}=\mathrm{-0},815V,\;\mathrm{SHE}\right)$$

##### 3.2.1.2 Electrolyzer Technologies for Hydrogen Production

The electrolyzer is the fundamental part of aqueous electrolysis. It consists of two electrodes immersed in an electrolyte solution, separated by an interface.^113,122^ Commercial electrolyzers consist of flow fields, precisely designed electrodes, nanostructured electrocatalysts, membranes, strongly acidic or alkaline electrolytes, multiple electrolysis cells arranged in stacks organized in series or parallel, water purification systems, gas conditioning units, heating systems, management units, controls for incoming fluid flows and outgoing gas flows, as well as energy management electronics.^123^ In particular, the membrane and catalyst in the electrodes significantly affect hydrogen production.^124^

Among the most widely used electrolyzers are the alkaline water electrolyzer (AWE) and the proton exchange membrane (PEM), which are technologies already implemented in the industry, as well as the solid oxide electrolyzer (SOE) and the anion exchange membrane (AEM), which have not yet reached a commercial stage. Tables 5 and 6 show the scientific advances in aqueous electrolysis over the centuries and the main characteristics of each electrolyzer, respectively.^125^

**Table 5:** Comparison of Technical Parameters Between the Main Electrolyzers: Reactions and Materials^a^

	Alkaline	PEM	AEM	Solid oxide
Anodic reaction	2OH^–^ → H_2_O +1/2O_2_ + 2e^–^	H_*z*_O → 2H^+^ +1/2O_2_ + 2e^–^	2OH^–^ → H_2_ O +1/2O_2_ + 2e^–^	O^2–^ → 1/2O_2_ + 2e^–^
Cathodic reaction	2H_2_O + 2e^–^ → H_2_ + 2OH^–^	2H^+^ + 2e^–^ → H_2_	2H_2_O + 2e^–^ → H_2_ + 2OH^–^	H_2_O + 2e^–^ → H_2_ + O^2–^
Separation	Diaphragm (Asbestos/Zifon/Ni)	Proton exchange membrane (Nafion)	Anionic exchange membrane (Sustinion, Fumatech)	Solid electrolyte YSZ
Cathode/catalyst	NiMo alloys	PGM	Transition metal-based materials – Ni	Ni/YSZ
Anode/catalyst	NiCo alloys	IrO_*x*_, RuO_*x*_	Transition metal-based materials – Ni	Perovskitas (LSCF, LSM) (La, Sr, Co, Fe) (La, Sr, Mn
Bipolar plates	Stainless steel/Ni-coated stainless steel	Ti, Pt/Au-coated Ti	Stainless steel/Ni-coated stainless steel	Co-coated stainless steel
Electrolyte	30–40% by weight KOH	Solid polymer electrolyte (PFSA)	DVB polymer support with KOH/NaOH	Yttria Stabilized Zirconia (YSZ)

a Adapted from refs. 113,126,127 .

**Table 6:** Comparison of Technical Parameters Between the Main Electrolyzers: Performance and Costs^a^

	Alkaline	PEM	AEM	Solid oxide
**Current density**	0.2–0.8 A cm^–2^	1–2 A cm^–2^	0.2–2 A cm^–2^	0.3–1 A cm^–2^
**Operation temperature**	60–90 °C	50–90 °C	40–80 °C	700–850 °C
**System efficiency**	68 – 77%	57–59%	50–83%	89% (lab)
**Gas purity**	99.5–99.9998%	99.9–99.9999%	99,9–99.9999%	99.9%
**Life cycle**	60,000 h	50,000–80,000 h	>30,000 h	20,000 h
**Estimated cost**	Low	High	Not available	Not available
**Technology status**	Mature	Commercial to small scale	R&D	R&D
**Electrode area**	10,000–30,000 cm^2^	1500 cm^2^	<300 cm^2^	200 cm^2^
**Voltage range**	1,4–3 V	1.4–2.5 V	1.4–2.0 V	1.0–1.5 V
**Pressure**	<30 bar	<70 bar	<35 bar	1 bar

a Adapted from refs. 113,126,127

PEM electrolysis is recognized for its rapid response times, making it well-suited for integration with renewable energy sources.^128^ However, it depends on Iridium oxide (IrO_2_) as the anode catalyst, which is favored for its stability in the acidic conditions characteristic of PEM electrolysis.^129^ An analysis conducted in 2025 suggests that scaling PEM capacity to achieve net-zero targets will necessitate nearly 30% of the entire annual global iridium supply. This supply is particularly concerning as iridium is only a minor byproduct of platinum and nickel mining operations predominantly in South Africa and Russia, with shortages anticipated as soon as 2030.^130,131^ This worrying supply constraint is fueling significant research into low-Ir loading catalysts and Anion Exchange Membrane (AEM) electrolyzers that leverage earth-abundant transition metals.^132,133^

Another effective type of electrolyzer, but still in the early stages of development, is the one based on molten carbonate. This technology involves reducing carbon dioxide or molten carbonates, which are then transformed into carbon monoxide or solid carbon. The electrochemical cell is formed by an electrolyte based on molten carbonate suspended in a porous, inert ceramic matrix, with the cathode formed by porous nickel alloyed with Cr and/or Al, and the anode formed by porous nickel oxide (NiO). A combination of lithium, potassium, and/or sodium carbonate (Li_2_CO_3_, K_2_CO_3_, and Na_2_CO_3_) is used as the electrolyte. This type of aqueous electrolysis occurs at high temperatures, in the 620 to 680 °C range. The reactions that govern this process are presented below (eqs 39 and 40).^113^

39 $${\mathrm{Cathode:}\;H}_{2}O_{\left(g\right)}+{\mathrm{4CO}}_{2(g)}+2e^{-}\to H_{2\left(g\right)}+CO_{3\left(l\right)}^{2-}$$

40 $$\mathrm{Anode}:CO_{3\left(g\right)}^{2-}\to CO_{2\left(g\right)}+\frac{1}{2}O_{2}+2e^{-}$$

##### 3.2.1.3 Exploring the Catalyst Landscape for Water Electrolysis

One of the major limitations of aqueous electrolysis is the slow kinetics of the HER and OER reactions, caused by their high overpotentials. The overpotential is a potential higher than the thermodynamic potential, capable of overcoming the kinetic barrier. The lower the overpotential, the higher the catalyst’s activity. This high value is linked to the multistep kinetics of the half-reactions, especially the oxygen gas evolution reaction, which has a higher overpotential than the HER. Catalysts reduce the overpotentials of the half-reactions toward the efficient production of oxygen and hydrogen gases.^134–136^

The use of acid electrolytes in PEM technology requires expensive noble metals or metal oxides as catalysts to prevent electrode corrosion, leading to higher cell costs. On the other hand, while alkaline electrolyzers offer the potential to use non-noble metals and metal oxides as catalysts, the catalytic activity in an alkaline medium is significantly lower. Either way, most of the state-of-the-art electrocatalysts for HER are made of platinum or other noble metals for both acid and basic electrolysis. As a result, the search for cost-effective, highly active, and long-lasting electrocatalysts suitable for different environmental conditions remains a significant challenge.^120^

The method of synthesis chosen for the catalyst directly affects the characteristics of the material and its performance. Among the most widely used methods are high-temperature pyrolysis (a widely adopted approach for atomically dispersed catalysts obtained from precursors such as metal–organic frameworks, polymers, graphene, and carbon nanotubes), electrochemical deposition (applied to modify the material’s surface or deposit new materials), photochemical reduction, and wet chemistry.^137^

Han et al.,^138^ for instance, utilized electrodeposition to produce a highly efficient MoO_2_@CoMo micronanoporous heterostructure catalyst at ambient temperature and pressure. This catalyst demonstrated high hydrogen evolution performance (overpotential of 76 mV for hydrogen evolution at −50 mA cm^–2^), attributed to the synergistic effect of the elements at the interface and the increased electrochemical surface area of the micronanoporous structure. The synthetic process is illustrated in Figure 9. Initially, a microporous CoMo structure was electrodeposited on a carbon paper substrate (CP), resulting in a customizable microporous coral-like CoMo structure. Subsequently, simultaneous Co dissolution and Mo oxidation were achieved through an electrochemical attack, leading to the formation of the nanoporous MoO_2_@CoMo surface. The passivated surface gave a higher stability for the catalyst in the long-term operation due to the maintained Mo^4+^ ratio as demonstrated by chronopotentiometry tests at −50 mA/cm^2^ for 10 h.

**Figure 9:**
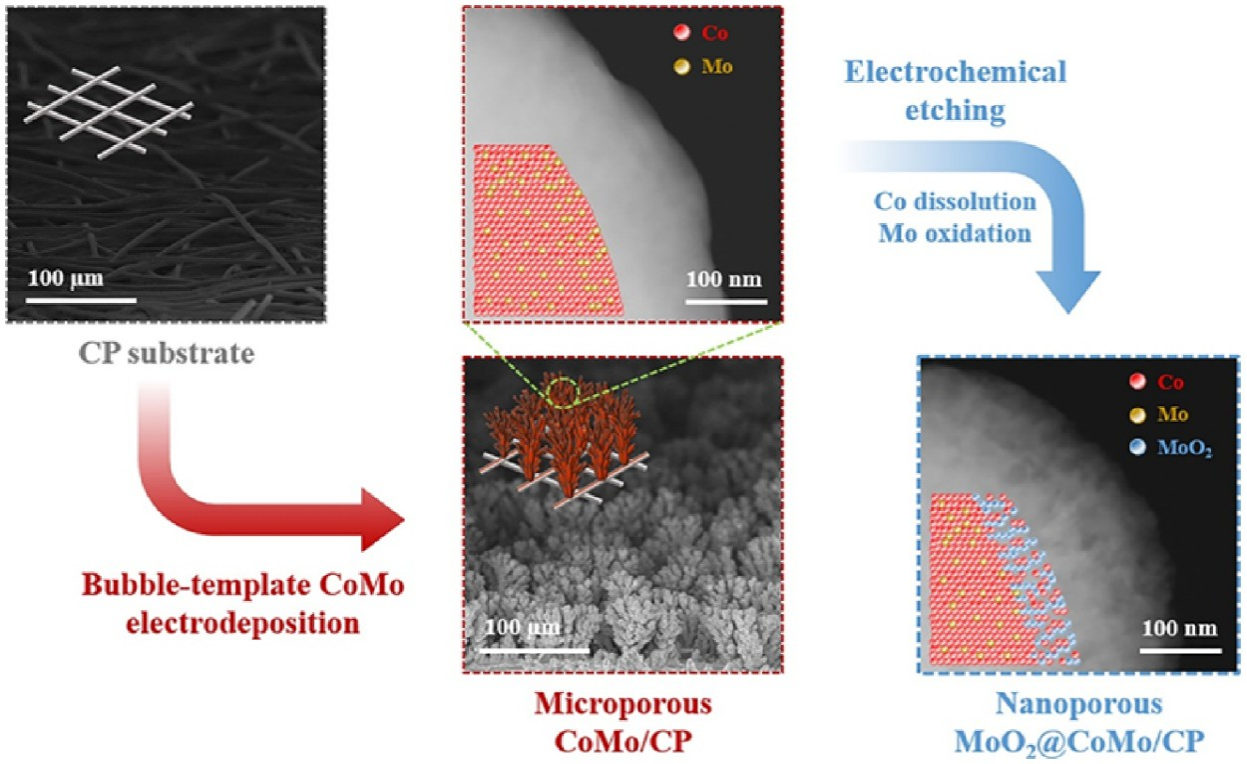
Schematic illustrating the synthesis of the MoO_2_@CoMo catalyst. Reprinted with permission from ref. 138. Copyright 2020, Elsevier.

Li et al.,^139^ on the other hand, developed an electrocatalyst through pyrolysis, evenly dispersing CoPt nanoparticles on carbon nanosheets, resulting in a catalyst with an exceptionally low overpotential of 19.1 mV at a current density of 10 mA cm^–2^ for HER. Even after pyrolysis at 800 °C for 2 h, the nanosheet structure remained well-preserved, with a lateral size of approximately 400 nm and a thickness of around 70 nm. Similarly, Kweon et al.^140^ incorporated a pyrolysis step in the synthesis of a Ru nanoparticle electrocatalyst anchored to multiwalled carbon nanotubes (MWCNT), leading to a catalyst with improved faradaic efficiency. Their synthesis scheme is illustrated in Figure 10.

**Figure 10:**
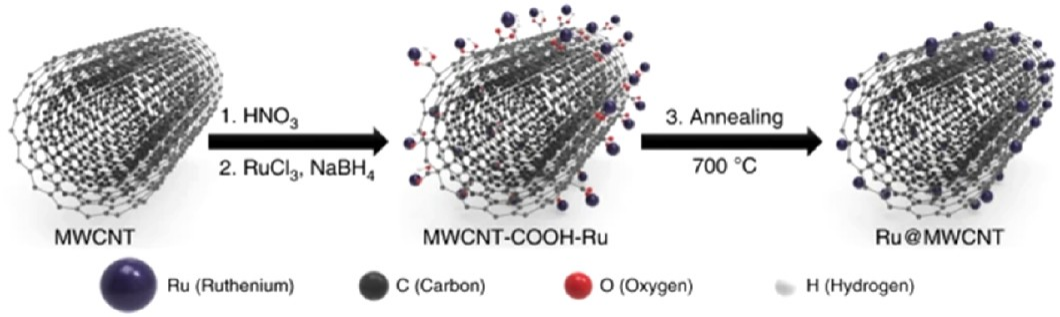
Schematic of the stages in the Ru@MWCNT catalyst formation process. Reprinted with permission from ref. 140. Copyright Creative Commons CC BY license 2020, Springer Nature.

Recent literature has emphasized the search for new materials with a catalytic performance similar to noble metals. The review by Falch and Babu, for example, demonstrates the versatility of electrocatalysts based on chromium (in metal alloys, oxides, phosphide hydroxides, and sulfides) for producing hydrogen from alkaline electrolysis.^141^ Metal–organic frameworks (MOFs) have also been extensively researched. Their appeal lies in their extensive surface area, numerous catalytic sites, and tunability, as various factors such as the metal center, type of ligand, presence of heteroatoms, and the formation of heterojunctions can influence their performance.^142^ In a study conducted by Cao et al.,^143^ a straightforward bimetallic iron–nickel MOF electrocatalyst coated with a nitrogen-doped graphene precursor was developed and implemented in water electrolysis, exhibiting an overpotential as low as 157 mV at 10 mA cm^–2^ for HER. It demonstrated a low cell voltage of 1.67 V and excellent stability over 24 h at 20 mA cm^–2^. The exceptional catalytic activity of the material can be attributed to its high specific surface area and pore size, characteristics derived from MOFs.

Tables 7, 8 and 9 outline various catalysts studied over the past five years. The catalysts are categorized based on their composition and characteristics, including details about reaction conditions and key findings highlighting their catalytic performance. The tables showcase a diverse range of materials, particularly non-noble metal-based catalysts achieving lower overpotentials than catalysts composed of noble metals such as platinum.

**Table 7:** Electrocalysts in Alkaline Electrolysis

Catalyst	Reaction conditions	Remarks	Ref.
2D Ni-based cyanide coordination polymer (2D Ni-CP)	KOH 1 mol L^–1^, 25 °C	Overpotential of 266 mV at 0.5 mA cm^–2^, Tafel slope of 186 mV dec^–1^, and charge transfer coefficient (α) of 0.32.	^144^
MoN/Co_2_N 2D hybrid nanosheets grown on 1D Cu nanowires and integrated into 3D Cu foam electrodes (Co/Mo–N–C/Cu)	KOH 1 mol L^–1^, 25 °C	Overpotential of 198 mV at 100 mA cm^–2^ and Tafel slope (131.8 mV dec-1). Faradaic efficiency close to 100%.	^145^
Mo-doped NiCo LDH nanosheets on FeCo_2_S_4_ nanosticks grown on 3 D nickel foam (FCS@M-NC LDH/NF)	KOH 6 mol L^–1^, 60 °C	Overpotential of 314 mV at 1000 mA cm^–2^. Tafel slope of 85.9 mV dec^–1^. Stability of 140 h at a current rate of 500 mV dec^–1^.	^146^
Nanoparticles and single Ru atoms supported on TiO_2_/C hybrid doped with N derived from MOF, forming two materials: Ru-NPs/SAs@N-TC and Ru-SAs@N-TC	KOH 1 mol L^–1^	Overpotential of 97 mV at 10 mA cm^–2^ and a Tafel slope of 58 mV dec^–1^. Stability of 1000 cycles for different pH media. TOF: 1.37 s^–1^ (η=50 mV).and 4.4 (η=100 mV).	^147^
Nanoporous Ni-based catalyst with Mo and B coaddition (NiMoB)	KOH 1 mol L^–1^	Overpotential 31 mV, Tafel slope (73 mV dec^–1^) and stability of 100 h (chronoamperometry and cyclic voltammetry).	^148^
Re NPs clusters doped with Pt and Ni interconnected by amorphous carbon (NPCs Pt–Ni@Re/C)	KOH 1 mol L^–1^, 25 °C	Activation energy of 16.2 kJ mol^–1^ and of TOF: 1.96 s^–1^.	^149^
Co–N_4_ isolated by adding sulfur atom	KOH 1 mol L^–1^	Overpotential value of 67.7 mV at 10 mA cm^–2^. Tafel slope: 56.3 mV dec^–1^. TOF: 0.21 s^–1^ (η = 50 mV) and 1.2 s^–1^ (η = 100 mV)	^150^

**Table 8:** Electrocalysts: Acid lectrolysis

Catalyst	Reaction conditions	Remarks	Ref.
Monometallic nanoparticles (M = Pt, Ir, Ru, Co, Ni) supported on graphene doped with N and P (M/rGNP)	H_2_SO_4_ 0.5 mol L^–1^, 25 °C	Best catalyst: Pt/rGNP. Low overpontetial 10.6 mV at 100 mA cm^–2^. Tafel slope 14.53 mV dec^–1^. Stability 6000 cycles and duarability of 72 h.	^151^
Integrated electrode formed by in situ grown platinum nanowires (PtNW/) in ultrathin liquid diffusion/titanium gas layers (LGDLs)	H_2_SO_4_ 0.5 mol L^–1^, 25 °C	Tafel slope of 35 mV dec^–1^ and faradaic efficiency of de 90.08%for the PEM electrolyzer.	^152^
Pt SAC and Pt NPs fabricated on a nitrogen-doped graphite foil substrate (Pt/NGF and Pt NPs/NGR)	H_2_SO_4_ 0.5 mol L^–1^, 25 °C	Pt/NGF: η = 2.2 mV at 10 mA cm^–2^, Tafel slope of 29.6 mV dec^–1^, Charge transfer resistance: 1.68 Ωcm^2^.	^153^
		Pt NPs/NGR: (η = 29 mV) at 10 mA cm^–2^, Tafel slope of 29.9 mV dec^1^.	
MoO_2_@CoMo heterostructured micronanoporous metal oxide applied to a single-cell PEM electrolyzer	H_2_SO_4_ 0.5 mol L^–1^, 90 °C	Overpotential of 76 mV at −50 mA cm^–2^, Tafel slope of 38.1 to 50.5 mV dec^–1^, Stability >10 h, Faradaic efficiency close to 100%.	^138^
Pt-based catalysts with low Pt concentration (PtSA/p-GO) and high Pt concentration (PtM/p-GO)	H_2_SO_4_ 0.5 mol L^–1^, 25 °C	PtM/p-GO: overpotential of 130 mV at 1000 mA cm^–2^, Tafel slope of 23 mV dec^–1^, Stability of 24 h at 1400 mA cm^–2^, TOF: 133.4 s^–1^	^154^
Amorphous nanoporous electrocatalyst PdCuNi-S	H_2_SO_4_ 0.5 mol L^–1^, 25 °C	Overpotential of 48 mV at 10 mA cm-2, Tafel slope: 35 mV dec^–1^, Stability: 24 h, TOF: 0.032 s^–1^	^155^
Co(OH)_2_ nanosheets decorated with MoO_3_ particles with a hierarchical nanostructure wrapped around Ag(MoO)_3_ -Co(OH) nanowires @Ag NWs	H_2_SO_4_ 0.5 mol L^–1^, 25 °C	Overpotential of 220 mV at −100 mA cm^–2^, Tafel slope: 48 mV dec^–1^, Stability of 29 h at 100 mA cm^–2^	^156^

**Table 9:** Electrocalysts: Alkaline, Neutral, and Acid lectrolysis

Catalyst	Reaction conditions	Remarks	Ref.
Atomic Ru anchored in chromium chips (CN/Cr_2_O_3_/Ru-1)	Alkaline: KOH 1 mol L^–1^; Acid: H_2_SO_4_ 0.5 mol L^–1^; Neutral: PBS 1 mol L^–1^. Twenty-five °C.	Alkaline medium was the best. Overpotential of 28 mV at 10 mA cm^–2^, Tafel slope of 58.6 mV dec^–1^, TOF of 1.89 s^–1^, Stability of 40 h.	^157^
Mo-doped CoP nanoparticles (Mo-CoP) supported and surrounded by a porous structure of carbon doped with single atomic Co (Co-NC) - (Mo-CoP/Co-NC)	Alkaline: KOH 1 mol L^–1^; Acid: H_2_SO_4_ 0.5 mol L^–1^; Neutral: PBS 1 mol L^–1^. Twenty-five °C	Tafel slope of 95 mV dec^–1^ in basic medium; 64 mV dec^–1^ in acidic medium; 101 mV dec^–1^ in neutral pH at 10 mA cm^–2^; Overpotential of 45 mV in basic medium; 41 mV in acidic medium; 98 mV in neutral pH. Stability >24 h for all pH values, TOF: 0.741 s^–1^.	^158^
Nanoclusters of molybdenum compounds (rGO/Mo_2_C, RGO/MoP and RGO/MoS_2_) supported on reduced graphene oxide doped with N	Alkaline: KOH 1 mol L^–1^; Acid: H_2_SO_4_ 0.5 mol L^–1^.25 °C.	rGO/MoP: overpotential of 67 mV at 10 mA cm −2, alkaline tafel slope: 52 mV dec-1 (alkaline) and 72 mV dec1 (acid); stability of 10000 cycles; durability of 24 h for the wide pH range, TOF: 8.05 × 10–2 s^–1^.	^159^
Controlled partial phosphorization to create CoP species in Co-MOF, producing a CoP/Co-MOF hybrid nanostructure	Alkaline: KOH 1 mol L^–1^; Acid: H_2_SO_4_ 0.5 mol L^–1^; Neutral: PBS 1 mol L^–1^.	Co-MOF: overpotential of 49 mV at 10 mA cm^–2^ at pH 7. CoP/Co-MOF: overpotential of 27 mV (acid), 34 mV (alkaline), 39 mV (neutral); Tafel slope (alkaline: 56 mV dec-1 (alkaline); 43 mV dec1 (acid); 63 mV dec1 (neutral); Stability of 20,000 cycles in all pH ranges.	^160^
Self-supported Cu catalysts with tensile stress	Alkaline: KOH 1 mol L^–1^; Acid: H_2_SO_4_ 0.5 mol L^–1^; Neutral: PBS 1 mol L^–1^.	Overpotentials of 182 mV (acid), 121 mV (alkaline), 261 mV (neutral), Tafel slope of 136.54 mV dec^–1^ (alkaline), 99.16 mV dec1 (acid), 143.58 mV dec^–1^ (neutral); Stability of 30 h; Faradic efficiency close to 100%.	^161^
Bioinspired mimetic catalyst of Fe_7_S_8_ nanoparticles incorporated into a polydopamine matrix electrocatalyst (Fe_7_S_8_/C)	Alkaline: KOH 1 mol L^–1^; Acid: H_2_SO_4_ 0.5 mol L^–1^; Neutral: PBS 1 mol L^–1^.	Overpotential of 107.4 mV (alkaline), 45.9 mV (acid), 143.58 mV (neutral); Average Tafel slope of 69.2 mV dec-1; Stability of 12 h, TOF of 2 × 10^–5^ – 32 × 10^–5^ s^–1^.	^162^

##### 3.2.1.4 Mechanistic Insights into Water Electrolysis Catalysis

The diagrams in Figure 11 represent the three possible steps for electrolysis in HERs, both in acidic and basic media.

**Figure 11:**
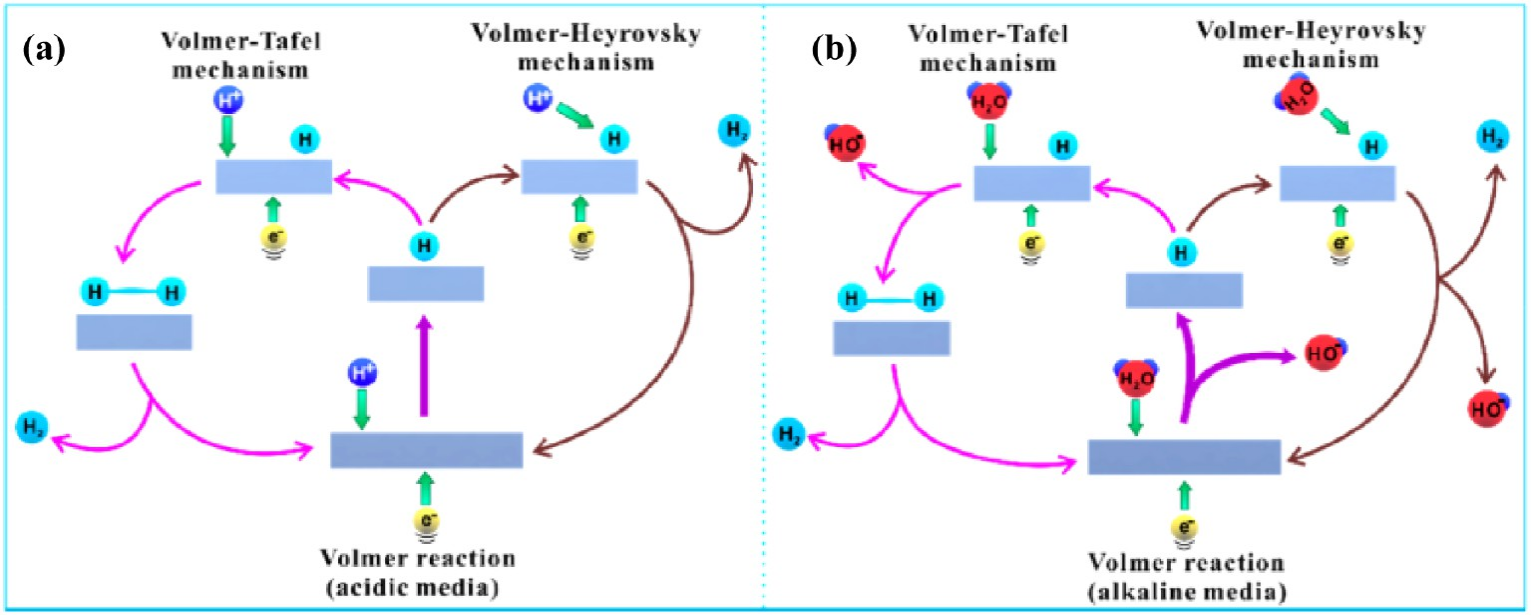
Mechanism of water electrolysis on the electrode surface in (a) acidic medium; (b) basic medium. Reprinted with permission from ref. 136. Copyright 2020, American Chemical Society.

In the first step, a proton reacts with an electron to generate a hydrogen atom adsorbed on the material’s surface (M), a process known as Volmer reaction. This proton can come from the hydronium ion (H_3_O^+^) (acidic medium, Figure 12a) or a water molecule (alkaline medium, Figure 12b). Subsequently, the formation of hydrogen gas can occur by two other reaction pathways, either in isolation or in combination. In the Heyrovsky reaction, a proton in solution combines with the hydrogen adsorbed on the electrode and reacts with a second electron to form H_2_. In the Tafel reaction, on the other hand, two hydrogens adsorbed on the electrode surface combine to form hydrogen gas. The steps are shown in eqs 41–45.^163^

**Figure 12:**
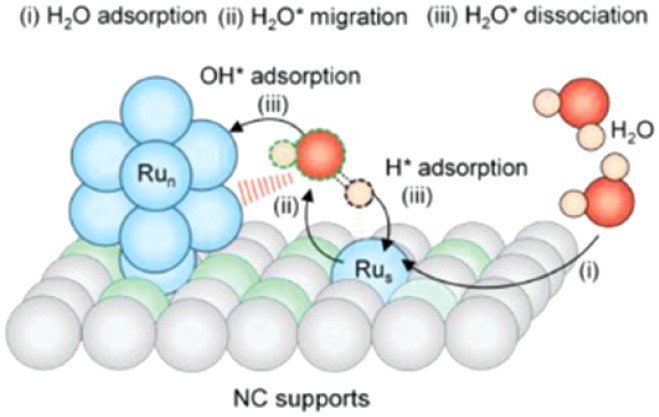
Schematic illustrating the mechanism of water dissociation by the catalyst formed from different ruthenium structures. Reprinted with permission from ref. 164. Copyright 2023, Wiley.

● Step 1: Electrochemical hydrogen adsorption reaction (Volmer reaction)

41 $$H_{3}O_{\left(aq\right)}^{+}+M+e^{-}⇌\mathrm{M-}H^{*}+H_{2}O_{\left(l\right)}\left(\mathrm{acidic medium}\right)$$

42 $$H_{2}O_{\left(l\right)}+\mathrm{M-}+e^{-}⇌\mathrm{M-}H^{*}+OH^{-}\left(\mathrm{alkaline medium}\right)$$

● Step 2: Electrochemical desorption (Heyrovsky reaction)

43 $$H_{\left(aq\right)}^{+}+e^{-}+\mathrm{M-}H^{*}⇌H_{2\left(g\right)}+M\left(\mathrm{acidic medium}\right)$$

44 $$H_{2}O_{\left(l\right)}+e^{-}+\mathrm{M-}H^{*}⇌H_{2\left(g\right)}+M+OH^{-}\left(\mathrm{alkaline medium}\right)$$

Or

● Step 3: Chemical desorption (Tafel reaction)

45 $$2M-H^{*}⇌H_{2\left(g\right)}+M\left(\mathrm{acidic or alkaline medium}\right)$$

Yang et al.^164^ conducted a theoretical analysis of the potential pathways for water dissociation in HERs within alkaline media, utilizing Ru-based catalysts. These catalysts are noteworthy due to their electronic structure, which is akin to that of platinum, as well as their improved cost-effectiveness. The research examined the water dissociation pathways (specifically the Volmer step) across Ru catalytic sites, including Ru clusters, single atoms, and their combinations, while evaluating the binding energy. The findings indicated that the mixed delocalization-localization electronic structure in Ru nanoclusters promotes the migration of H_2_O* and the orientation of bonds during the dissociation process. The authors proposed a mechanism (Figure 12) whereby the ruthenium materials adjust the adsorption energy of H_2_O, effectively integrating the distinct preferences of Ru nanoclusters and Ru single atoms for OH* and H* species, thus facilitating efficient water dissociation in alkaline media.

The simultaneous reaction in the OER electrode involves the transfer of four protons and electrons to the metal center (the active site, M). Initially, the hydroxyl ions are adsorbed on the active sites of the material to form M–OH (eq 46). After this step, the M–OH intermediate forms M–O by deprotonation and electron release (eq 47). The M–O product reacts with hydroxyl ions to form an M–OOH intermediate (eq 48), followed by the production of oxygen gas at the end of the reaction accompanied by deprotonation of the intermediate and regeneration of the M active site (eq 49). An alternative route can occur by the combination of two M–O intermediates, which are converted into O_2_. In reactions that take place in acidic media, the same intermediates mentioned above are formed.^165^ The diagram in Figure 13 schematizes the mechanism of the reaction.

**Figure 13:**
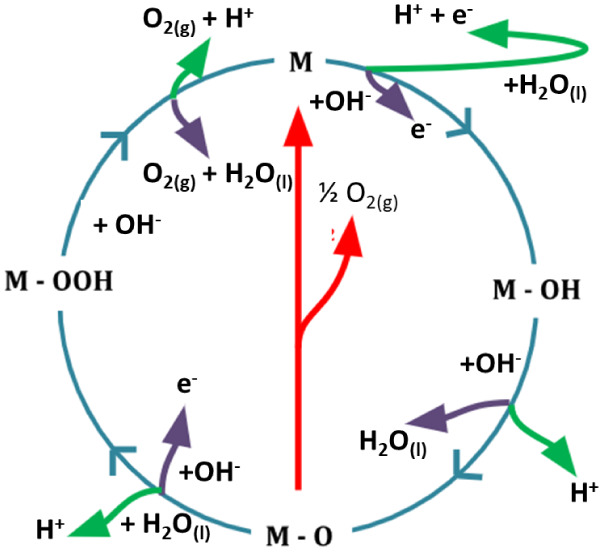
OER mechanism for acid electrolytes (green) and alkaline electrolytes (purple). Reprinted (adapted) with permission from ref. 166. Copyright Creative Commons CC BY license 2022, Springer Nature.


46 $$OH^{-}+M⇌\mathrm{M-}OH+e^{-}$$

47 $$\mathrm{M-}OH+OH^{-}\to \mathrm{M-}O+H_{2}O+e^{-}$$

48 $$\mathrm{M-}O+OH^{-}\to \mathrm{M-}OOH+e^{-}/2\mathrm{M-}O\to O_{2}+2e^{-}$$

49 $$\mathrm{M-}OOH+OH^{-}\to O_{2}+H_{2}O+e^{-}+M$$

A significant advancement in the literature from 2024 to 2025 is the deepening of the recognition of catalyst self-reconstruction. While non-noble metal compounds have demonstrated effectiveness, the mechanisms underlying their activity, driven by structural reconstruction, remain inadequately understood, largely due to the lack of effective *in situ* and operando techniques.^167^ Many materials recognized as high-performance catalysts for the OER, such as MOFs and phosphides, are actually functioning as “pre-catalysts.” These materials can transform *in situ* into the thermodynamically stable active phase, typically metal oxyhydroxides (NiFeO_*x*_H_*y*_), under operational conditions.^167,168^ For example, Binyamin et al. (2024) showed that MOF precatalysts could be electrochemically converted into active catalysts (NiFeOOH), demonstrating that the MOF acts as a “sacrificial template” for the generation of “MOF-converted OER Ni_1–x_Fe_*x*_OOH electrocatalysts”.^168^ Similarly, Wu et al. (2025) discovered that phosphides, serving as precatalysts, can be transformed *in situ* into highly active OER electrocatalysts, eventually evolving into (oxy)­hydroxide structures. This further supports the notion that phosphide crystal structures can convert into metal (oxy)­phosphides or (oxy)­hydroxide structures under OER conditions.^167^ Understanding the dynamic evolution of these surfaces is now considered more critical than the initial static crystal structure paradigm.^169^

##### 3.2.1.5 Assessing the Performance of Electrocatalysts for Hydrogen Production

The performance of an electrocatalyst is evaluated based on its stability, activity, and efficiency.

Stability refers to the ability of catalysts to maintain consistent activity throughout the reaction and over time. It can be assessed by monitoring overpotential changes and exchanging current over time. The exchange current density reflects the charge transfer on the electrode surface at equilibrium; a higher exchange current density leads to a lower overpotential.^120^ To evaluate stability, researchers conduct chronoamperometry or chronopotentiometry experiments on the electrodes. In chronoamperometry, the current is varied over time while keeping the potential constant, while in chronopotentiometry, the potential is varied while keeping the current constant.

Higher stability is indicated by more prolonged constant current and potential, as depicted in Figure 14. For comparison, many studies have used a current density higher than 10 mA cm^–2^ and a minimum time of 10 h as a reference for considering a catalyst stable. Another method for assessing stability is voltammetry, which analyzes changes in the overpotential factor before and after a given series or cycle. Optimum material stability is indicated by minimal changes in overpotential values after thousands of cycles.^120,136^

**Figure 14:**
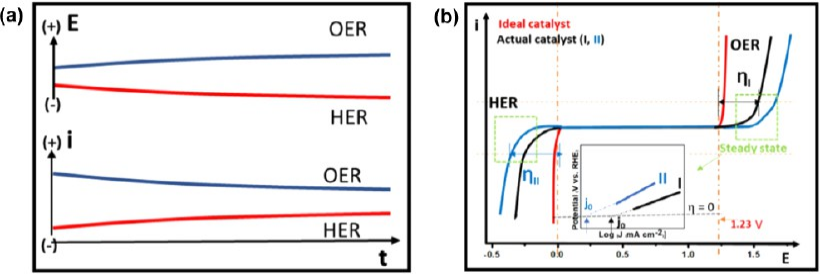
(a) Stability measured in terms of current and potential curves over time; (b) activity in terms of overpotential, Tafel slope, and current density. Reprinted with permission from ref. 120. Copyright Creative Commons CC BY license 2021, Springer Nature.

Mondal et al.^170^ developed a platinum material containing small amounts of germanium (to reduce costs). The material’s stability was assessed using chronoamperometry, demonstrating a stability of 500 h at a current density of 10 mA cm^–2^ in an acidic medium. Ha et al.^171^ developed a Ni_3_S_2_ nanorod matrix catalyst codoped with Li and V, grown on a Ni foam substrate using a one-step hydrothermal method. The catalyst’s stability at high current densities was evaluated through multichronopotentiometry tests at current densities of 500, 1000, and 2000 mA cm^–2^ for 30 h. It was observed that the potentials at 500 and 1000 mA cm^–2^ did not show significant increases even after 20 h of operation.^172,173^

Catalytic activity is often assessed by analyzing the overpotential, Tafel slope, and exchange current density.^120^ The Tafel slope and exchange current can be determined from the overpotential versus kinetic current graph (Figure 14a) and eq 50.^136^ Setting the overpotential to zero in eq 48 yields the Tafel slope (b) and the current density (J_0_). A lower slope corresponds to a higher current density relative to the overpotential. In essence, a lower Tafel value indicates a faster reaction with a lower overpotential. Additionally, the Tafel slope is closely linked to the HER and can indicate the rate-determining step in the reaction. Therefore, for high catalytic activity, the ideal catalyst should exhibit a low Tafel slope and a high exchange current density.

50 $$\eta =a+b\;\log\;\log{\;J}_{0}$$

where *η* is the overpotential and J_0_ is the current density.

The final factor under consideration is catalytic efficiency, which quantifies the production of oxygen and hydrogen gas at the electrodes during electrolysis. Efficiency is estimated using faradaic efficiency and turnover frequency. Faradaic efficiency is a common parameter used to measure this factor, and it quantitatively assesses the number of migrating electrons needed for the reaction to occur at the electrode surface. Faradaic efficiency (eq 51) is determined by calculating the ratio of the experimental amount of hydrogen and oxygen gas produced to the theoretically calculated amount.

51 $$\mathrm{Faradaic efficiency}=\frac{H_{2}\;or{\;O}_{2}\left(\mathrm{experimental}\right)}{H_{2}\;or{\;O}_{2}\left(\mathrm{theoretical}\right)}$$

The turnover frequency (TOF), on the other hand, is calculated from eq 52. The TOF is a measure of activity used to quantify the number of reactions catalyzed by a catalyst’s active site per unit time.

52 $$\mathrm{TOF}=\frac{JN_{A}}{nFr}$$

where N_A_ is the Avogadro number, and *r* is the concentration rate of the catalyst’s active sites.

The volcano graph is another commonly used method for evaluating and understanding a catalyst. It illustrates the relationship between current density and variation in the Gibbs free energy of adsorption (ΔG), which indicates the best materials for catalyzing a reaction. For a catalyst to be considered adequate, the interaction between the reaction intermediates and the catalyst surface should ideally have a ΔG equal to zero for high current density values. If the interaction is weak (ΔG > 0), only a few intermediates have bound to the catalyst surface, slowing down the reaction. Conversely, if the interaction is very strong (ΔG < 0), the products do not dissociate and hinder the reaction, blocking the active sites. The volcano graph can also be presented in terms of current density versus reaction activity. For instance, Figure 15 demonstrates the versatility of this approach by showing the volcano graph for the HER reaction in acidic media, where platinum outperforms other metals due to its high current density value.^136^

**Figure 15:**
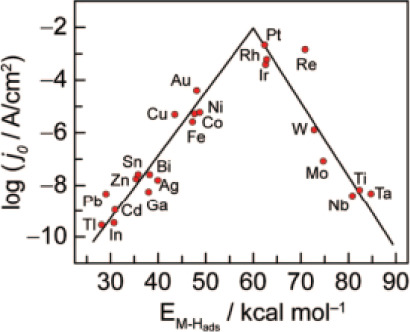
Volcano curve for HER on metal electrodes in acidic media. The logarithm of the current density J_0_ is represented. Reprinted with permission from ref. 172. Copyright 1972, Elsevier, and reprinted with permission from ref. 173. Copyright 2010, American Chemical Society.

#### 3.2.2 Photocatalytic Water Splitting: Harnessing Light for Hydrogen Production

##### 3.2.2.1 Understanding the Photochemical Basis of Water Splitting

The photocatalytic splitting of water has attracted significant attention since Fujishima and Honda’s groundbreaking report in 1972, which revealed that TiO_2_ can catalyze the splitting of water under light irradiation to produce hydrogen.^174,175^ This process, known as photocatalytic water splitting or water photolysis, holds great promise for environmentally friendly H_2_ production. It theoretically allows water to be the sole reactant, facilitating reduction and oxidation reactions that lead to the formation of H_2_ and O_2_ gases, respectively.^176^

The photosplitting of water (eq 53) is a highly endothermic reaction (ΔG°= +237.24 kJ mol^–1^), requiring a minimum electric potential of +1.23 V to break the water molecule into H_2_ and O_2_ (eq 51).^177–179^

● General equation:

53 $$H_{2}O\to H_{2}{+1/2O}_{2}$$

This method offers the biggest advantage of its gentle reaction conditions and the direct use of light as an abundant and renewable energy source. The process involves two steps: the separation/transfer of photogenerated charge and then surface chemical reactions.^180^ When exposed to light with an energy higher than the band gap, the electrons (e^–^) in the valence band are promoted to the conduction band, leaving holes behind (h^+^). The higher energy of the incident light is required due to the activation barrier in the charge transfer between the catalyst and the water molecules. The semi-reactions of the water splitting are shown in eqs 54 and 55, with Figure 16 indicating the schematics of the reaction.^176,177^

**Figure 16:**
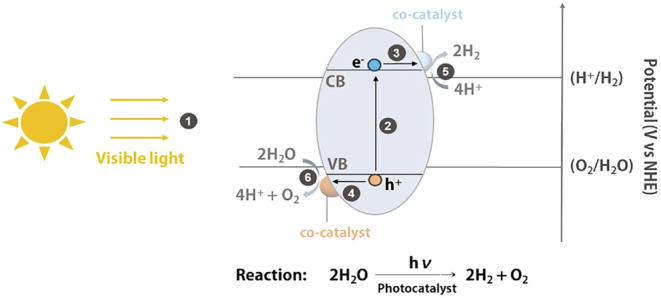
Schematics of overall water splitting on a semiconductor particle. CB, conduction band. VB, valence band. Reprinted with permission from ref. 181. Copyright 2022, Elsevier.

● Conduction band:

54 $${\mathrm{2 H}}^{+}{+2e}^{-}\to H_{2},E=0V\left(\mathrm{vs.}\mathrm{NHE at pH 0}\right)$$

● Valence band:

55 $${\mathrm{2 H}}_{2}{O+\mathrm{4 h}}^{+}\to O_{2}{+\mathrm{4 H}}^{+}\;E=+1.23V\left(\mathrm{vs.}\mathrm{NHE at pH 0}\right)$$

When selecting a suitable photocatalyst for H_2_ evolution, the bandgap width and the charge recombination rate at the catalyst’s active sites should be considered. The band potentials for water oxidation and reduction must fall within the band gap of the photocatalyst, making semiconductors with well-defined band structures (1.23 eV < E_g_ < 3.26 eV) well-suited for this purpose.^181,182^

Conventionally, photocatalysts based on TiO_2_ (E_g_∼3.2 eV) (Figure 1) and ZnO (E_g_∼3.2 eV) are the most widely used choices for water splitting under visible light due to their cost-effectiveness and low environmental impact.^181^ Additionally, other inorganic semiconductors such as oxides, sulfides (e.g., CdS and MoS_2_), nitrides, Bi_2_WO_6_, and BiOCl have also been extensively studied (Figure 17).^178,183^

**Figure 17:**
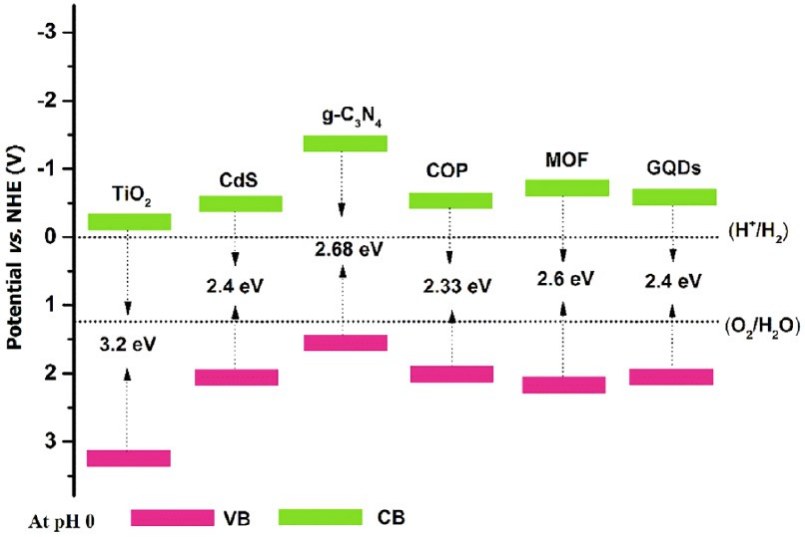
Illustration of the band edges of different types of semiconductors. VB = valence band; CB = conduction band. Reprinted with permission from ref. 181. Copyright 2022, Elsevier.

Ng et al.^176^ utilized CdS@ZIF for photocatalytic water splitting, achieving a hydrogen generation rate of 519 μmol g^–1^ h^–1^, with an apparent quantum efficiency (AQE) increase of 8 to 13 times compared to emerging photocatalytic designs that employ organic hole scavengers. In another study, Zhao et al.^184^ developed MoS_2_/Cd-ZnIn_2_Se_4_/CdS composites through an in situ structure-tailoring technique. This composite exhibited an optimal hydrogen evolution rate of 11.49 mmol g^–1^ h^–1^, which is approximately 4.79 times higher than that of pristine ZIS (ZnIn_2_Se_4_), which had a rate of 2.40 mmol g^–1^ h^–1^.

Semiconductors acting as photocatalysts for hydrogen production can produce photogenerated electrons when under ultraviolet (UV), visible, and/or near-infrared (NIR) light.^185^ When electrons become excited, they can migrate to the conduction band (CB), creating electron (e^–^) and hole (h^+^) pairs. The energy gap between the valence band (VB) and the conduction band is known as the “forbidden band”. A semiconductor absorbs light when its wavelength matches the width of the forbidden band. Following excitation, the photogenerated electrons and holes primarily travel to the semiconductor’s surface. The width of a semiconductor’s band gap influences its light-absorbing capacity. The positions of the conduction (CB) and valence (VB) bands, on the other hand, determine the photocatalyst’s redox capacity.^186,187^

##### 3.2.2.2 Challenges and Limitations of Photocatalytic Water Splitting

The recombination of carriers and the slow kinetics of water splitting in photocatalysts present significant obstacles to their practical application.^188^ A promising advancement in this area is using ultrasound-assisted piezoelectric photocatalysis to enhance H_2_ evolution through water splitting. Piezoelectric materials such as ZnO and BaTiO_3_ demonstrate the piezoelectric effect when exposed to ultrasonic radiation, facilitating water splitting to produce H_2_.^189^

By combining TiO_2_ with a piezoelectric material (quartz) with a weak piezopotential, for instance, Jin et al.^190^ achieved a hydrogen rate more than 8 times higher than the photocatalyst alone (541.9 μmol g^–1^ h^–1^ compared to 65.6 μmol g^–1^ h^–1^). This significant enhancement occurs due to the distortion caused by the piezoelectric effect on the band structure of the catalyst, minimizing the recombination. Their study also brought light to the effect of the particle size in modulating the piezoelectric effect, with a direct impact on the hydrogen production enhancement or inhibition, bringing up the theoretical concept of “piezopotential switch”.

Although low-cost inorganic photocatalysts with high AQE are available, their wide band gaps limit them to the UV region (4.4% of the solar spectrum). Therefore, research increasingly aims to reduce semiconductor band gaps, a key trend for improving photocatalytic performance.^191^

Metal-free semiconductors are emerging as promising alternatives for photocatalytic water splitting due to the scarcity of inorganic semiconductors that can be activated by visible light. Examples include g-C_3_N_4_ (graphitic carbon nitrides), linear conjugated polymers, conjugated microporous polymers, covalent triazine-based frameworks, and covalent organic frameworks.^183^ In a notable study, Yan et al.^192^ made the pioneering effort to combine covalent organic frameworks with hexagonal boron nitride (h-BN) to create an effective metal-free photocatalyst (Figure 18). This composite demonstrated exceptional photocatalytic hydrogen production performance in metal-free systems. The integrated porous h-BN effectively suppresses electron backflow, thereby optimizing the photocatalytic activity of the composite.

**Figure 18:**
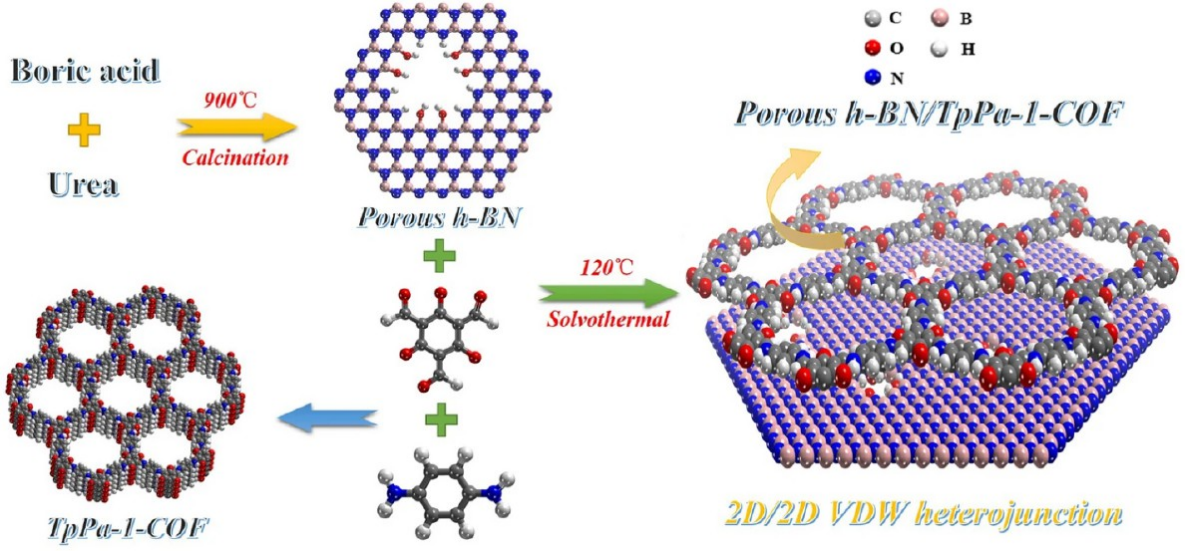
Schematic diagram of the synthesis route for porous h-BN/TpPa-1-COF composites. Reprinted with permission from ref. 192. Copyright Creative Commons CC BY license 2023, Springer Nature.

Fang et al.^193^ developed nonmetallic carbon nitride catalysts through a rigid mold method. These synthesized materials were utilized for solar thermophotocatalytic hydrogen production from water, employing triethanolamine as a sacrificial agent. The top-performing catalyst achieved an impressive hydrogen generation rate of 1932.9 μmol g^–1^ h^–1^.

##### 3.2.2.3 The Role of Sacrificial Agents in Photocatalytic Hydrogen Production

Current photocatalytic methods often rely on organic molecules as sacrificial agents, such as lactic acid and methanol, to facilitate the photocatalytic cycle by consuming photoholes during the oxidation reaction.^176^ The selection of the sacrificial donor and its subsequent oxidation is critical for determining the efficiency of photocatalysis, as these factors can significantly influence hydrogen production measurements in semiconductor-assisted photocatalysis.^194^

When chemicals are introduced to enhance charge transfer in photocatalytic processes as sacrificial electron donors or acceptors (Figure 19), the complexities of product analysis and mass balance increase. Since the utilization of sacrificial electron donors (SEDs) has emerged, significantly higher hydrogen outputs are often reported. Nonetheless, despite the common use of sacrificial donors in photocatalysis and claims of high efficiency, the actual role of these donors in the process is rarely thoroughly assessed.^194,195^

**Figure 19:**
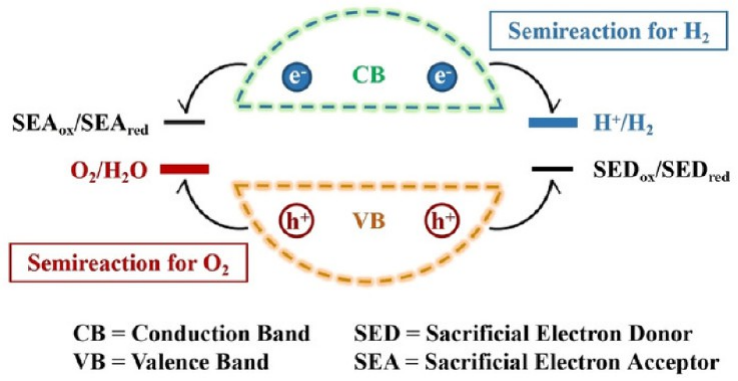
Schematics of sacrificial electron donors or acceptors in a photocatalytic process. Reprinted with permission from ref. 186. Copyright 2020, Wiley.

As demonstrated by Hainer et al.,^195^ predicting catalytic performance in actual water splitting poses significant challenges (Figure 20). Their study compared hydrogen (H_2_) generation both with and without the inclusion of sacrificial electron donors (SEDs), utilizing seven TiO_2_-based catalysts under solar simulation conditions (AM 1.5) and 368 nm LED irradiation. Methanol and formic acid were chosen as SEDs because of their common use by the radicals produced after hole trapping (•CH_2_OH and •CO_2_^–^), acting as excellent electron donors. While the presence of SEDs markedly enhanced H_2_ generation, these improvements varied considerably depending on the specific catalyst used. Thus, advancing SED-assisted processes requires a comprehensive understanding of the free radical chemistry generated from gap trapping.

**Figure 20:**
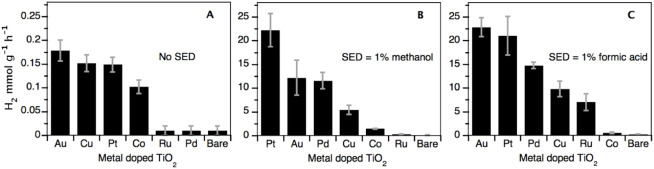
H_2_ generation rates by different catalysts tested using 368 nm irradiation (0.33 W cm^–2^) for (A) true water-splitting conditions, (B) in the presence of 1% methanol, and (C) in the presence of 1% formic acid (pH ≈ 2.2). Photocatalytic Hydrogen Generation Using Metal-Decorated TiO _2_: Sacrificial Donors vs True Water Splitting. Reprinted with permission from ref. 195. Copyright 2018, American Chemical Society.

To avoid the use of sacrificial agents, Ng et al.^176^ in their work, demonstrated the creation of a multifunctional interface between the photocatalyst and the metal–organic framework (MOF), allowing for efficient charge transfer and optimizing irradiation conditions, such as light intensity and duration, can also increase the efficiency of the photocatalysis process.

Tables 10 and 11 present a collection of recent studies on catalysts used in the hydrogen evolution reaction from water photolysis, detailing the system conditions, including whether sacrificial agents were employed.

**Table 10:** Photocatalysts for the Water Photolysis Reaction:With Sacrificial Agent

Catalyst	System’s conditions	HGR/(μmol g^–1^h^–1^)	Ref.
2D Mo_2_C/ZnIn_2_S_4_	250 mL automatic online catalytic system with 100 mL of aqueous solution containing 10 mL of triethanolamine. Xe lamp (300 W) (>400 nm). Twenty mg of catalyst, room temperature. Sacrificial agent: Et_3_N.	22110	^175^
Pt–Au	Photocatalytic activity evaluation system with 100 mL of aqueous solution containing 20% by volume of 0.75 mol L^–1^ ascorbic acid. 300 W xenon lamp. 50 mg of catalyst, room temperature. Sacrificial agent: ascorbic acid.	1.88 × 10^3^	^180^
Tp-DBN and Pt(3%)	Closed gas circulation and evacuation system with 100 mL of aqueous solution. 300 W Xe lamp, λ> 420 nm. Twenty mg of catalyst, 5.85 °C. Sacrificial agent: ascorbic acid.	1.8 × 10^3^	^183^
Carbon nitrides (M-CN and C–CN)	Commercial glass reactor with an internal diameter of 50 mm. 300 W Xe lamp. 40 mg of catalyst, 60 °C. Sacrificial agent: Et_3_N (10%).	808.4 and 1932.9	^193^
Co_3_O_4_@C(7 wt %)/TiO_2_	Pyrex glass reactor connected to a flow system with 200 mL methanol solution (20 vol %), ultraviolet light-emitting diode (UV-LED) lamp (25 W, 365 nm). 50 mg of catalyst, 25 °C. Sacrificial agent: Et_3_N (10%).	11400	^196^
Pt single-sites on hexagonal ZnIn_2_S_4_	Reactor in anaerobic condition with 45 mL of aqueous solution. 300 W Xe lamp. Twenty mg of catalyst, 8 °C. Sacrificial agent: Et_3_N (10%).	17.5 × 10^3^	^197^
*sp* ^2^ *c* -Py-BT COF and o-*imina* -Py-BT COF	Quartz tube with 50 mL of water, xenon lamp (300 W) (>420 nm). Thirty mg of catalyst, room temperature. Sacrificial agent: Et_3_N (10%).	891.5	^198^
Re-CNN/CNN/Ox-CNN (Pt 6%)	Glass Pyrex with temperature connected to a closed gas system with 100 mL of aqueous solution. Xe lamp (300 W) (λ > 360 nm). Ten mg of catalyst, 25 °C. Sacrificial agent: Et_3_N (10%).	127.05 × 10^3^	^199^
CuO/ZnO	300 mL quartz reactor with 100 mL of aqueous solution. 300 W Xe lamp (λ > 420 nm). 50 mg of catalyst, 25 °C. Sacrificial agent: Et_3_N (10%).	48.6 × 10^3^	^200^
OMIM]Br/FeCl_3_	Reactor with 200 mL of aqueous solution. 500 W halogen lamp (λ= 550 nm). One g of catalyst, room temperature. Sacrificial agent: MeOH.	243.2 × 10^3^	^201^

**Table 11:** Photocatalysts for the Water Photolysis Reaction:Without Sacrificial Agent

Catalyst	System’s conditions	HGR/(μmol g^–1^ h^–1^)	Ref.
CdS@ZIF	12 mL quartz tube with 3 mL of water, illuminated with a 100 W white light LED (400–750 nm). 2.6 mg of catalyst, room temperature.	519	^176^
Carbon ring conjugated TCN (C-TCN) and Pt(3%)	Online photocatalytic analysis system with 100 mL of water. 300 W Xe lamp, λ > 400 nm. 50 mg of catalyst, room temperature.	204.6	^202^
IMBA–rGO	Sealed quartz cell with 80 mL of water. 500 W Xe lamp. Five mg of catalyst, room temperature.	16.49	^203^
DAnTMS/CD	40 mL glass bottle with 20 mL of water. Visible light, 420 nm ≤ λ ≤ 700 nm. Ten mg of catalyst, room temperature.	265.0	^204^
RhCrOx/STO:Al/N-G.	51 mL cylindrical quartz reactor with 25 mL of water. 300 W Xe lamp. Twenty-five mg of catalyst, 25 °C.	64 × 10^3^	^205^
N-TiO _2_/MgO (111)	25 mL stainless steel autoclave system equipped with two quartz windows (10 mm in diameter and 18 mm thick) with 10 mL of water. 70 W tungsten lamp. Five mg of catalyst, 270 °C.	11,092	^206^
PbTiO _3_ /Rh/Cr_2_O_3_	Automatic 250 mL analysis system with 100 mL of aqueous solution. Xe lamp (300 W). 100 mg of catalyst, room temperature.	3.29 μmol h^–1^	^207^
B–Zn_3_As_2_	Sealed 182 mL quartz reactor with 120 mL of water. Xe lamp (λ = 750 nm). Thirty mg of catalyst, room temperature.	228.59	^208^
2D rGO/SnS_2_/Ag	Closed vacuum gas circulation system with 100 mL water top window. 300 W high-pressure Xe lamp (λ ≥ 420 nm). 50 mg of catalyst, 5 °C.	137	^209^

##### 3.2.2.4 Metal-Free Photocatalysis: The Potential of Organic Semiconductors

The initial exploration of organic photocatalysts for solar hydrogen production dates back to 1985, when Yanagida et al. first reported the use of linear poly­(p-phenylene)­s for hydrogen evolution under UV light (λ > 366 nm).^210^ Organic photocatalysts present diverse molecular structures that can be readily modified to optimize their optoelectronic properties, including bandwidth and light absorption range, enhancing their suitability for specific applications.^179^ Moreover, organic semiconductors provide numerous structural combinations, allowing for precise synthesis tailored to industrial needs, such as minimizing the release of toxic substances during production and ensuring ease of decomposition without posing an environmental burden.^191^

Widely researched organic photocatalysts encompass several families of materials, including graphitic carbon nitrides (g-C_3_N_4_), linear conjugated polymers (LCPs), conjugated microporous polymers (CMPs), and covalent organic frameworks (COFs). Generally, organic photocatalysts tend to be less toxic and more cost-effective than many inorganic alternatives, making them particularly appealing for large-scale applications.^211^

The mechanism of water splitting utilizing organic photocatalysts consisting of three key steps: (1) Organic materials absorb incident photons of appropriate energy, resulting in the generation of excitons, which are bound electron–hole pairs. (2) These excitons then diffuse through the materials. (3) When they reach the interface between the materials and water molecules, the excitons dissociate into free positive and negative charge carriers, enabling the reduction and oxidation of water to produce hydrogen fuel.^179^

Elewa et al.^212^ demonstrated robust hydrogen evolution induced by visible light using triazine-conjugated microporous polymers (CMPs) doped with sulfur. The triazine-based CMPs exhibited excellent photocatalytic performance, achieving hydrogen evolution rates of 108.1 and 116.5 μmol h^–1^ under visible light for Py-TPT-CMP (pyridine—Titanium­(IV) isopropoxide—CMP) and TPT-TPT-CMP (Titanium­(IV) isopropoxide—Titanium­(IV) isopropoxide—CMP), respectively, with quantum efficiencies of 41.9% and 32.38%. They observed that while the TPT-based CMP doped with sulfur experienced a reduction in hydrogen evolution rate efficiency compared to its nondoped counterpart, it showed enhanced stability in photocatalytic hydrogen evolution.

### 3.3 Biological, Biomimetic, and Bioinspired Processes

Many microorganisms can naturally produce hydrogen while they metabolize the organic matter. There are various biological technologies available for hydrogen production, including direct and indirect biophotolysis and photofermentation, as well as processes that can occur in the absence of light, such as dark fermentation and bioelectrolysis.^213^ Hydrogenases, the class of enzymes responsible for hydrogen production, can also be used directly or as a source of inspiration for synthesizing catalysts for hydrogen production.^214,215^ This section will focus on remarking examples where catalysts or “catalytic systems” were synthesized to enhance natural biological processes or were developed based on biological models, rather than “purely” biological systems. For those interested in the latter, numerous recent reviews are available.^216–222^

#### 3.3.1 Processes in the Dark

##### 3.3.1.1 Natural Hydrogen Production in the Dark

According to Łukajtis et al.,^213^ dark fermentation, where some microorganisms produce hydrogen in the absence of light, is the most promising method for producing hydrogen from biomass. For comparison, the net energy ratio of this technology (output energy over input energy) is about 1.9, which is significantly higher than the ratio of 0.64 achieved through steam methane reforming.

Figure 21 provides a representation of the biohydrogen production through dark fermentation by anaerobic communities using two phylogenetically different hydrogenases: [FeFe]-hydrogenases and [NiFe]-hydrogenases.^213^ These hydrogenases facilitate the removal of electrons from organic substrates, which are then used to reduce protons.^223^ The glycolysis-based fermentation pathway underlying this process is well established. Initially, glucose is converted into pyruvate through glycolysis, with the concomitant generation of NADH as a reducing equivalent. Under anaerobic conditions, pyruvate is converted to acetyl-CoA by pyruvate ferredoxin oxidoreductase (PFOR), producing CO_2_ and reduced ferredoxin, which serves as an electron donor to hydrogenases for hydrogen production. Alternatively, pyruvate may be metabolized by pyruvate formate lyase (PFL), yielding acetyl-CoA and formate; the latter is subsequently converted into H_2_ and CO_2_ by hydrogenases. Acetyl-CoA further acts as a central metabolic intermediate for the formation of various fermentation end products, including ethanol, organic acids, and solvents, alongside NADH oxidation and ATP generation.^213^

**Figure 21:**
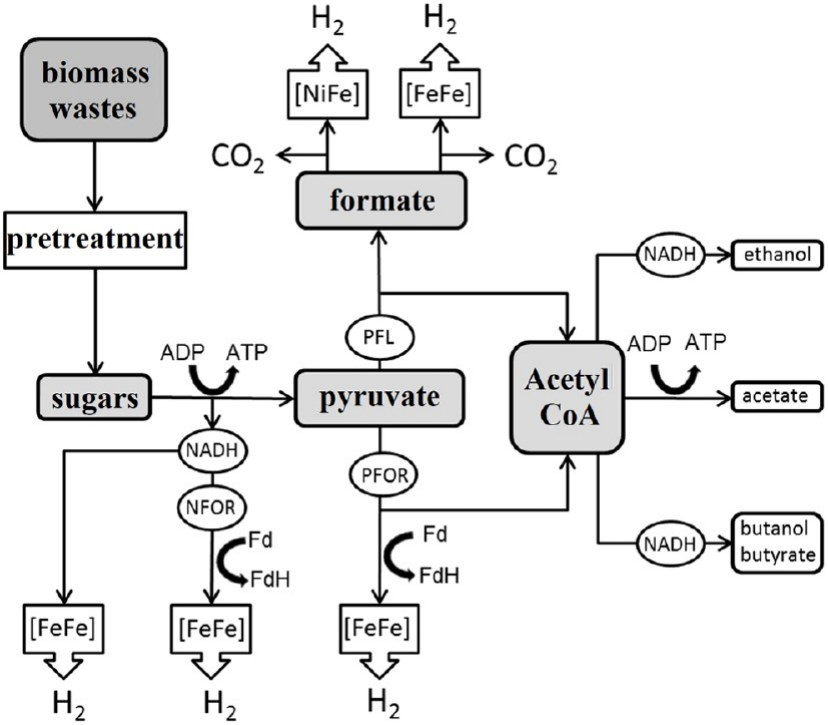
Production of biohydrogen in dark fermentation by an anaerobic community. ADP- Adenosine Diphosphate; ATP-Adenosine Triphosphate; NADH- Nicotinamide Adenine Dinucleotide; NFOR- NADH-ferredoxin oxidoreductase; Fd- Ferredoxin (Fd); FdH- Reduced Ferredoxin; PFOR- Pyruvate-Ferredoxin Oxidoreductase; PFL- Pyruvate Formate Lyase; Acetyl CoA- Acetyl coenzyme A. Reprinted with permission from ref. 213. Copyright 2018, Elsevier.

##### 3.3.1.2 Enhancing Hydrogen Production in the Dark

To further enhance hydrogen production in these microorganism communities, researchers have explored coupling them with conductive materials to facilitate the so-called Direct Interspecies Electron Transfer (DIET).^223^ Additionally, nanoparticles added to microbial systems can act as enzyme cofactors or activators, participate in redox reactions, and regulate gene expression.^224^

In 2024, Jamaludin and colleagues^223^ conducted a study where they combined a composite of coconut shell-derived biochar and nickel and magnetite nanomaterials (GAC-NiFe_3_O_4_) with a microorganism community primarily consisting of Thermoanaerobacterium. They varied several system parameters, including pH, temperature, and agitation, to enhance the biohydrogen production of the microorganism community. The GAC-NiFe_3_O_4_ exhibited a conductivity of 10.64 ± 0.36 μS cm^– 1^, 1.68 times higher than the control (GAC without NiFe_3_O_4_ nanoparticles). The enhanced conductivity enabled microbial attachment through DIET, leading to a hydrogen production rate of 3.96 ± 0.62 mmol L^–1^ h^–1^ and overall hydrogen production at least eight times higher than the control.

A study conducted by Ye et al.^224^ utilized a newly isolated anaerobic consortium and nickel ferrite nanoparticles (NiFe_2_O_4_ NPs) to enhance biohydrogen production from a mixture that comprised swine wastewater and rice straw hydrolyzate (2:3). With the addition of 200 mg L^–1^ of NiFe_2_O_4_ NPs, the cumulative hydrogen production per liter of wastewater increased by 21.8% compared to the control, resulting in 1560.4 ± 10.3 mL of hydrogen. The microbial community analysis revealed the predominance of Clostridium sensu stricto and Enterococcus species. Additionally, genetic analysis showed that the presence of the nanoparticles favored the expression of the hydrogenase gene by almost 200 times after 24 h of fermentation.

#### 3.3.2 Light-Driven Biological Hydrogen Evolution: The Power of Photosynthesis

##### 3.3.2.1 Nature’s Solar-Powered Hydrogen Factories

As previously mentioned, in natural settings, hydrogen photoproduction is usually associated with biophotolysis and photofermentation. Biophotolysis involves aerobic microorganisms, including cyanobacteria (Anabaena sp., Nostoc sp.) and microalgae (Chlorella sp., Chlamydomonas sp., Nannochloropsis sp., Scenedesmus sp.), which can convert light energy and water into H_2_ with or without the need for nutrients or substrates.^225^

Through the process of direct biophotolysis, light is absorbed by the photosystem II (PSII), causing the electrons produced to reduce ferredoxin (Fd). This reduction drives the production of H_2_ by the hydrogenase enzyme, which reduces protons. In indirect photolysis, light energy is used in the fixation of CO_2_ into carbohydrates. This helps microalgae/cyanobacteria lower oxygen levels in situ, increasing the hydrogenases’ activity.^225^

Photofermentation, on the other hand, involves anaerobic photosynthetic bacteria, such as *Rhodobacter* spp., *Rhodovulum* spp., *Rhodopseudomonas* spp., and *Rhodospirillum* spp. These microorganisms can absorb a wide range of light (400–900 nm) and consume various organic substrates. Unlike other microorganisms, they lack photosystem II (PSII). Instead, the catabolism of the organic substrates furnishes electrons to the plastoquinone pool, which are then transferred to the photosystem I (PSI), where light is absorbed. The electrons are then transferred to ferredoxin and then to hydrogenases, which will catalyze the generation of molecular hydrogen.^225^

##### 3.3.2.2 Enhanced Photosynthetic Hydrogen Production

Recent advancements in biohydrogen production have progressed beyond basic fermentation processes to embrace sophisticated bionano hybrid systems. These systems integrate living organisms with synthetic materials to address inherent kinetic bottlenecks. A key challenge in this arena is effectively managing the electron transfer interface between biological photosystems and synthetic catalysts, all while ensuring cell viability and mitigating oxygen sensitivity.

A notable strategy in this field involves reengineering the electron transport pathways of living cells to facilitate synthetic catalysis. For example, Edwards et al.^226^ and Gwon et al.^227^ have both demonstrated the successful harnessing of intracellular electrons for hydrogen evolution, albeit through opposing directions of electron flow. Gwon et al. innovatively engineered *Chlamydomonas reinhardtii* by integrating carbon nanofibers (CNFs) directly into chloroplasts, thereby establishing “electron highways” that extract photosynthetic electrons outward to surface-bound platinum (Pt) catalysts. This intracellular extraction effectively circumvents the insulating barrier of the cell wall, allowing the system to operate solely on sunlight and carbon dioxide while achieving a photon-to-electron conversion efficiency of approximately 0.7%. In contrast, Edwards et al. employed the electrogenic bacterium *Shewanella oneidensis* MR-1 to deliver respiratory electrons inward to cadmium selenide (CdSe) quantum dots (QDs) (Figure 22). In this setup, the bacteria protect the semiconductor photocatalyst from oxidative degradation by replenishing electrons, facilitating continuous hydrogen production for over a week. While Gwon et al. directly harnessed photosynthetic potential, Edwards et al. leveraged the bacteria’s ability to metabolize lactate, a common component of wastewater, thus linking waste remediation with energy production.^226,227^

**Figure 22:**
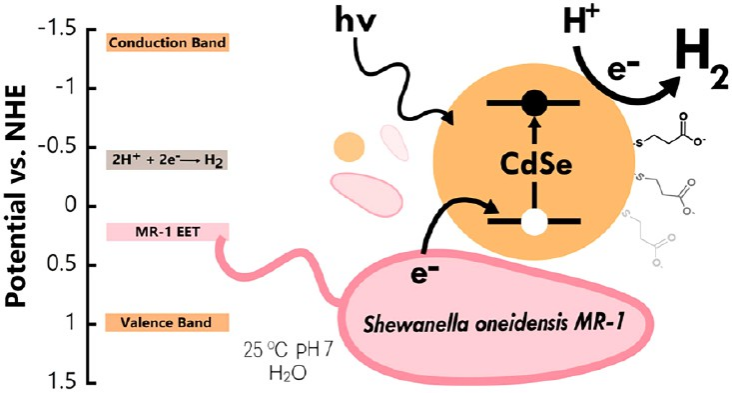
Schematic drawing of the biosystem showing the electron transfer from MR-1 to the holes in the valence band of CdSe nanocrystals, replenishing electrons for the catalytic evolution of H_2_ by the reduction of the protons in the medium. Reprinted with permission from ref. 226. Copyright Creative Commons Attribution-NonCommercial-NoDerivatives License 4.0 (CC BY-NC-ND) 2023, PNAS.

The stability and longevity of biohybrid systems represent a significant advancement over traditional enzymatic or isolated catalyst methods. Peptide-based mimics, such as the new artificial hydrogenase designed by Parambath et al.,^228^ offer important mechanistic insights into the role of protonated cysteine residues and the pH dependence (reaching its peak at pH 5.6) within the catalytic cycle. However, these mimics frequently face challenges with limited stability, lasting only a few hours, and their reliance on costly sacrificial donors like ascorbic acid. In contrast, whole-cell biobionic approaches have exhibited remarkable durability. For instance, Gwon et al.‘s “electron highway” system sustained production for over 50 days (Figure 23), while the bionic algal system developed by Xu et al. achieved an impressive hydrogen evolution duration of 200 days^227,229^ .

**Figure 23:**
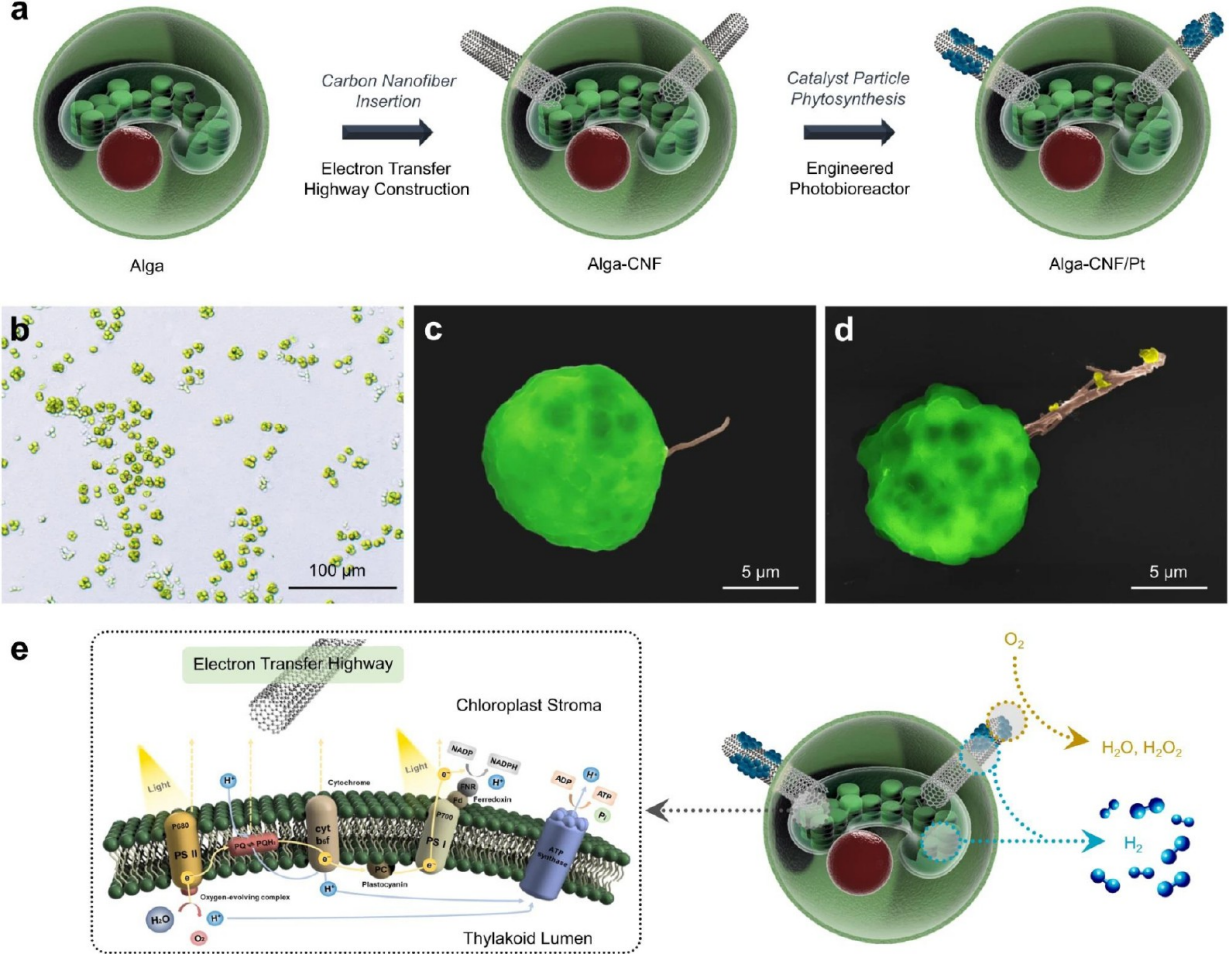
(a) Schematic depiction of the algae engineering process; (b) Optical micrograph of *Chlamydomonas reinhardtii* cells; (c), (d) Scanning electron micrographs of alga-CNF and alga-CNF/Pt composite, respectively; (e) Schematic representation of the electron extraction by a CNF highway from the inside of the thylakoidal membrane. Reprinted with permission from ref. 227. Copyright Creative Commons CC BY license 2023, Springer Nature.

Xu et al.^229^ accomplished this impressive longevity through a “cellular bionics” approach, encapsulating *Chlorella pyrenoidosa* within a conductive polypyrrole (PPy) layer and a calcium carbonate exoskeleton. In contrast to the CNF insertion method, which physically penetrates the chloroplast, Xu et al. engineered a localized hypoxic microniche using the Fe­(III)-doped polymer shell. This design consumes oxygen and activates hydrogenases even in aerobic conditions (Figure 24). The encapsulation effectively addressed two significant challenges: it protected the hydrogenase from oxygen inhibition and provided a conductive interface for accepting extracellular electrons, thereby enhancing production rates to 4.4 μmol mg_chl_^–1^ h^–1^. Additionally, the CaCO_3_ shell functioned as a pH buffer, preventing the acidification of the medium that typically restricts long-term batch cultures.

**Figure 24:**
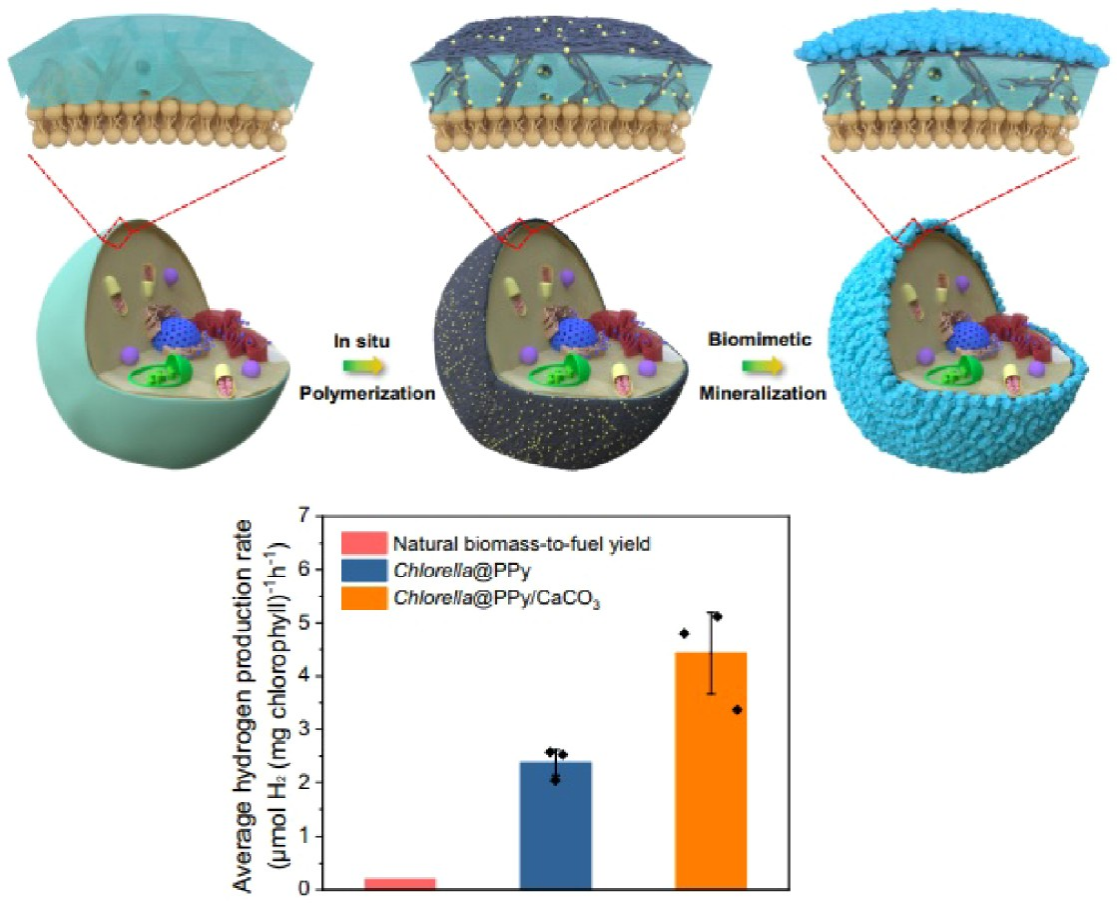
On the top: Schematic showing the stepwise construction of concentrically arranged polypyrrole polymer and CaCO_3_ thin shells on the cell wall of *Chlorella pyrenoidosa* (yellow spheres: Fe­(III) ions; black inner shell: polymer; blue outer shell: CaCO_3_). At the bottom: Average hydrogen production rates (determined between 2–7 days) for nonengineered cells, polymer-coated cells, and polymer/CaCO_3_-coated algal cells. Reprinted with permission from ref. 229 Copyright Creative Commons CC BY license 2023, Springer Nature.

Despite these advancements, trade-offs concerning scalability, toxicity, and cost remain unresolved. The system developed by Gwon et al. stands out for its scalability and autoregulation of oxygen, enabling the production of gas pure enough to directly power a commercial fuel cell.^227^ However, its reliance on platinum, a rare noble metal, may impede economic viability at industrial scales. Edwards et al. employed cadmium-based quantum dots, raising potential environmental toxicity concerns; however, they demonstrated that the bacteria remained viable at catalytic concentrations.^226^ Xu et al.^229^ showed that dead cells with functional shells could continue to produce hydrogen for up to 8 days, suggesting that the structural stability provided by abiotic coatings can somewhat decouple production from cell viability.^229^ Collectively, these studies underscore a shift toward integrating self-repairing biological machinery with robust inorganic interfaces, though future efforts must address the elimination of noble metals and the long-term environmental impact of releasing engineered nanobiohybrids.

## 4 Catalysis for On-Demand Hydrogen Generation from Chemical Hydrides

Hydrogen storage, whether in physical or chemical forms, is crucial for hydrogen-based energy systems. Among the various chemically stored hydrogen options, inorganic hydrides and organic compounds stand out as safe and efficient alternatives. In this context, catalysts play a vital role, as they lower the energy required for hydrogen release, speed up reaction rates, and enhance overall efficiency.^230,231^

The literature contains numerous comprehensive reviews highlighting the use of catalysts for hydrogen release from different storage systems.^232–234^ While the primary focus of this review is to present the catalysts employed in hydrogen production, a brief overview of advancements in the hydrogen release field is also provided, particularly emphasizing the following inorganic hydrides: ammonia borane (NH_3_BH_3_), hydrous hydrazine (N_2_H_4_·H_2_O), and hydrazine borane (N_2_H_4_BH_3_).

### 4.1 Catalytic Hydrolysis of Inorganic Hydrides for Hydrogen Generation

Lightweight inorganic hydrides that incorporate metals from groups 1 and 2, such as lithium (Li), sodium (Na), magnesium (Mg), and calcium (Ca), alongside elements from groups 13 and 15, including boron (B), aluminum (Al), and nitrogen (N), have drawn attention due to their stability, nontoxicity, and capacity for hydrogen storage with on-demand release. These compounds effectively balance volumetric and high gravimetric energy densities, which is essential for developing practical energy storage systems. They can contain both protic hydrogens (N–H) and hydridic hydrogens (B–H), exhibiting opposing polarities. Under both pyrolysis and hydrolysis conditions, their utilization can yield ultrapure hydrogen (H_2_), free from carbonaceous contaminants.^235^

#### 4.1.1 Releasing Hydrogen from Ammonia-Borane via Catalysis (NH_3_BH_3_: AB)

Ammonia-borane (AB), a white solid, represents the simplest compound of boron and nitrogen. It has a melting point ranging from 112 to 114 °C and a density of 0.780 g cm^–3^. Notably, AB contains 19.6 wt % hydrogen, is nontoxic, and exhibits high water solubility (33.6 g per 100 mL). Additionally, it remains stable under mild conditions and is environmentally friendly. These exceptional properties make AB a promising candidate for hydrogen storage. Hydrogen can be generated from AB through either thermolysis or solvolysis processes (hydrolysis or methanolysis). The thermolysis process generally requires higher temperatures and can produce products that are difficult to manage. However, with the right catalysts, hydrogen production via the hydrolysis of AB can be achieved under ambient conditions, yielding 3 mol of hydrogen gas for every 1 mol of AB (eq 56).^236–238^

56 $${\mathrm{NH}}_{3}{\mathrm{BH}}_{3}{+2H}_{2}O\to {3H}_{2}{+\mathrm{NH}}_{4}{\mathrm{BO}}_{2}$$

Investigating catalysts for the complete hydrolysis of ammonia borane (AB) to produce hydrogen at room temperature is of significant importance. In recent years, a range of transition metal nanoparticles (NPs), including Rh,^239,240^ Ru,^241,242^ Ni,^243–248^ Co,^249–253^ Cu,^254–256^ and Pt,^237,243,257–265^ have been explored as potential catalysts for this process. Table 12 presents a summary of recent findings in the literature. For noble metals, various support materials have been employed in AB hydrolysis. These supports include carbon-based options such as carbon dots,^266^ graphene,^267^ and carbon nanotubes,^268^ and metal–organic frameworks (MOFs),^256,269,270^ as well as noncarbon-based alternatives like SiO_2_,^271^ TiO_2_,^272^ CeO_2_.^273^

**Table 12:** Selected Examples of the Recent Literature on the Performances of the Catalysts for Hydrolysis of NH_3_BH_3_

				Stability activity		
Catalysts	T/(°C)	TOF/[min^–1^]	Ea/(kJmol^–1^)	Cycles	Retainedactivity/(%)	Ref
Rh/o-Ti_3_C_2_T_*x*_	25	2021	18.7	5	53	^239^
Rh/UiO-66-NH2	25	876.7	22.3	5	63	^240^
CF-BT-Ru	25	322	32.41	5	76	^241^
Ni_1_Ru_1_/TCN	25	1046.2	24.3	8	100	^242^
Ni_0.4_Cu_0.6_Co_2_O_4_/Ti nanoleaf-like array	25	60.3 (NaOH)	23.5	7	100	^245^
Ni_0.23_Co_0.19_P_0.58_@NHPC900	25	282.4	51.61	5	75	^246^
Ni_1.2_Fe_0.8_@CN-G	25	23.25	38.24	5	100	^247^
meso-Ni_10.0_Co_74.5_B_15.5_AASs	25	6.50	38.62	4	100	^248^
CoNi/α-MoC	25	321.1 (NaOH)	-	10	100	^249^
Co@Co_2_Mo_3_O_8_	25	17.28	51.8	5	100	^275^
Co-CoP-NC/NF-2	30	10	30.6	-	-	^250^
Co_3_B-CoP/h-BN	25	56 (NaOH)	51.8	5	100	^251^
multishelled Co–P	25	23.5	38.7	7	100	^252^
rGO/CoNi-N	25	126.0	32.8	10	100	^253^
CuPd@ZIF-67@ZIF-8	30	30.2	38.8	5	63	^254^
Cu_0.5_@Co_0.5_-MOF/5	25	129.8	26.5	10	74	^255^
CuFeCo@MIL-101	25	23.2	37.1	7	60	^256^
Pt/MoO_3_–x	25	2268.6	13.97	5	90	^257^
NiTi-LDH@B_12_H_12_@Pt 2.4%	25	454.06	41.82	5	59.81	^259^
Pt_1_/Co_3_O_4_-c	30	6035	35.7	10	97	^260^
Pt/TiO_2_	25	307	39.4	5	100	^261^
Pt-PVP/SiO_2_	25	371	46.2	5	100	^262^
Pt^0^/Co_3_O_4_	25	4366	71	10	100	^263^
Pt^0^/CoFe_2_O_4_	25	3628	65	10	100	^276^
PtNiO_X_TV_0_	25	618.0	59.3	10	80	^264^
Pt/Co_3_O_4_	25	721	31.3	10	87	^265^
Ru@carbon quantum dot (CQD)	30	361	33.32	6	100	^266^
CoNiP/GO-30	25	269.2	44.12	5	84.6	^267^
Co@Co_2_P/N–CNP	30	18.4	32.1	3	100	^268^
Pd/Fe_3_O_4_@SiO_2_-*g*-C_3_N_4_	30	33.7	31.4	8	100	^270^
Ru@CoNC-67-M	25	1031	21.93	5	97	^271^
Ru_1_Co_9_/TiO_2_	25	1408	33.25	10	100	^272^

Guan et al.^274^ developed a multisite catalyst comprising Ru and Pt, employing the impregnation-reduction method. In this design, Ru and Pt atoms served as the active metal sites, while Ti atoms in Ti_3_C_2_ acted as the support. This catalyst exhibited remarkable catalytic activity, achieving a high turnover frequency (TOF) of 1293 min^–1^, exceeding that of most noble metals. The Ru site effectively activated the B–H bond, whereas the Pt sites facilitated the activation of the O–H bond. Furthermore, the Ti site improved the adsorption of the *H intermediate due to the synergistic effects of the multisite arrangement (Figure 25).

**Figure 25:**
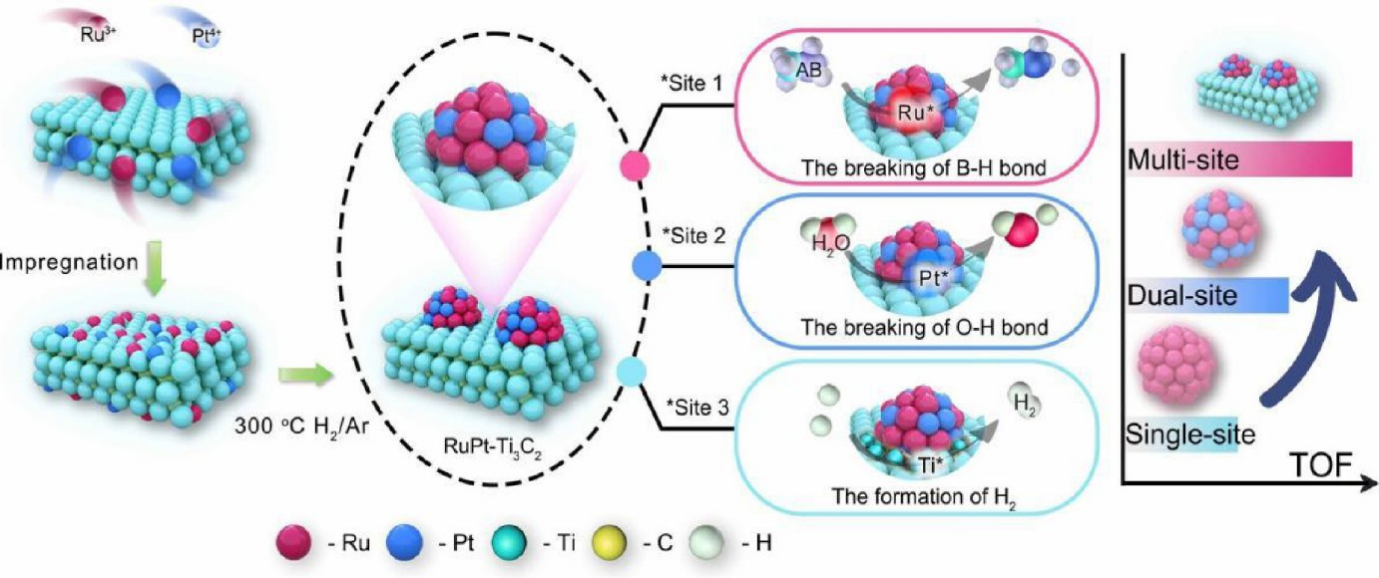
Schematic illustration for the preparation of RuPt Ti, and multisite tandem activation mechanism for AB hydrolysis. Reprinted with permission from ref. 274. Copyright 2024, Wiley.

Through a meticulous three-step synthesis process, Chen et al.^267^ developed an efficient and elegant catalyst: CoNiP nanobox on graphene oxide (CoNiP/GO). The optimized CoNiP/GO catalyst achieved a turnover frequency (TOF) of 134.6 min^–1^ for hydrogen production from aqueous AB at 25 °C in the presence of NaOH. Remarkably, the CoNiP/GO catalyst retained 85% of its initial activity after five cycles. The outstanding catalytic performance of CoNiP/GO is likely a result of its distinctive morphology, tailored electronic structure, and supportive base (Figure 26).

**Figure 26:**
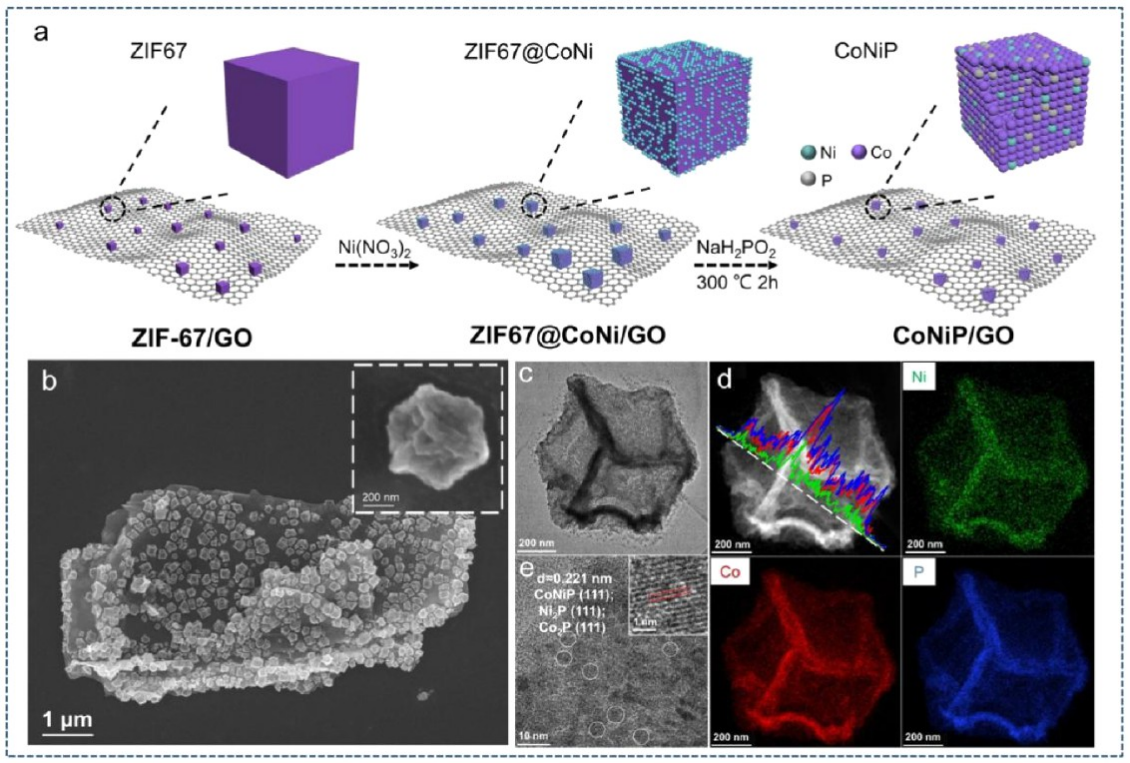
(a) Schematic illustration of the synthetic process for CoNiP/GO. (b) SEM image of CoNiP/GO (Ni^2+^ etching 30 min). (c) TEM image of CoNiP/GO (Ni^2+^ etching 30 min). (d) Dark-field TEM image and the corresponding elemental mappings of CoNiP/GO (Ni^2+^ etching 30 min): Ni (green), Co (red), and P (blue). (e) High-resolution TEM image of CoNiP/GO (Ni^2+^ etching 30 min). Reprinted with permission from ref. 267. Copyright 2022, Elsevier.

##### 4.1.1.1 Understanding the Mechanism of Hydrogen Release from AB

The mechanism for the bimolecular activation of ammonia borane (AB) and water was first described by Wang et al.,^243^ by it can differ based on the characteristics of the catalyst and the reaction conditions. Shen et al.^264^ presented a catalytic mechanism for the hydrolysis of AB utilizing PtNiO_*x*_TVO. In their catalyst, oxygen vacancies can function as active reaction sites (Figure 27A). Water is adsorbed via the O–H bond to the oxygen vacancy (VO1) adjacent to the platinum (Pt) atom. This O–H bond experiences cleavage, leading to the formation of Pt–H* and VO1-OH*. Concurrently, ammonia borane (NH_3_BH_3_) adsorbs onto the platinum atoms through its boron atom and decomposes into Pt–H* and Pt-NH_3_BH_2_*. The VO1-OH* then reacts with Pt-NH_3_BH_2_* to generate NH_3_BH_2_OH. Finally, the hydrogen atoms bonded to platinum (Pt–H*) aggregate on the Pt surface to produce the first hydrogen gas (H_2_).^264^

**Figure 27:**
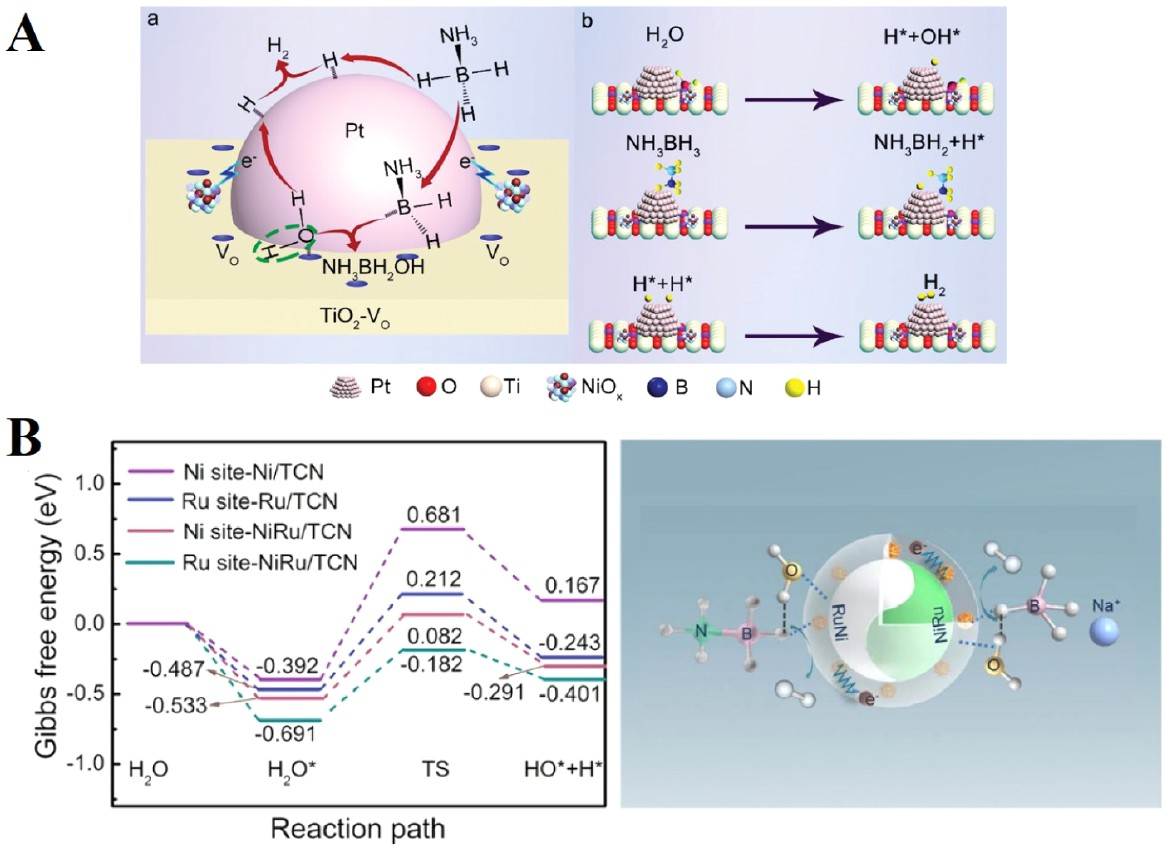
A) Dual activation mode of Pt and oxygen vacancy. Reprinted with permission from ref. 264. Copyright 2022, Wiley. B) The *E*_a_ of water molecules is at different sites of the Ni1Ru1/TCN catalyst. Reprinted with permission from ref. 242. Copyright 2021, Elsevier.

Liu et al.^242^ proposed that during the hydrolysis process, a hydrogen bond ([H_3_NBH_2_H]···H–OH) forms between NH_3_BH_3_ and H_2_O, attributed to the hydridic nature of the B–H bond (Figure 27B). When NH_3_BH_3_ and H_2_O molecules come into proximity with the synthesized catalyst surface, NiRu/TCN, the electron-rich ruthenium (Ru) atom is likely to activate the B–H bond within the NH_3_BH_3_ molecule, while the electron-deficient nickel (Ni) atom tends to activate the O–H bond in the water molecule. Consequently, the two activated hydrogen atoms combine to produce hydrogen gas (H_2_) molecules.^242^

#### 4.1.2 Releasing Hydrogen from Hydrous Hydrazine (N_2_H_4_·H_2_O)

N_2_H_4_·H_2_O presents a promising option for hydrogen storage due to its high storage capacity (8.0 wt % H), reasonable cost, and acceptable stability under ambient conditions. Although hydrazine has an even higher hydrogen capacity (12.5 wt %), it poses significant safety risks due to its high toxicity and explosive reactions when in contact with metal catalysts. Notably, the water molecule in N_2_H_4_·H_2_O does not participate in the decomposition process, resulting in products that mirror those of hydrazine itself: hydrogen and nitrogen (eq 57). However, a competitive side reaction pathway (eq 58) can occur, which significantly diminishes the hydrogen selectivity of the decomposition reaction. This side reaction produces ammonia, an environmentally harmful compound toxic to catalysts. Consequently, there is a pressing need to develop highly selective and efficient nanocatalysts to ensure the complete conversion of N_2_H_4_·H_2_O into hydrogen while minimizing the risk of incomplete decomposition.^277–279^ Table 13 presents a summary of recent findings in the literature.

57 $$N_{2}H_{4}\to {2H}_{2}{+N}_{2}$$

58 $${3N}_{2}H_{4}\to {\mathrm{4NH}}_{3}{+N}_{2}$$

**Table 13:** Selected Examples of the Recent Literature on the Performances of the Catalysts for Dehydrogenation of N_2_H_4_·H_2_O

					Reusability		
Catalysts	Temp./(°C)	NaOH/(mol L^–1^)	TOF/(h^–1^)	E_a_/(kJ mol^–1^)	Run (No)	Activity/(%)	Ref.
NiFe-La(OH)_3_	70	1.5	100.6	57.8	5	100	^278^
NiPt/NC	50	2	1602	48.3	10	65	^284^
NiRuPt/SiO_2_	60	5.0	324.1	50.2	5	72	^285^
Ni_0.6_.Pt_0.4_/La_2_O_2_CO_3_	25	0.3	490	56.7	5	100	^286^
NiMo/TiO_2_	70	9	484	54.3	10	100	^287^
Ni/Fe/Pd	40	0.5	25.3	32.1	5	100	^289^
Co/ZnO@NiFe_2_O_4_	25	7	4445.37	44.84	15	100	^290^
Pt_3_Ni_2_ nanowires	50	2	726	60.5	5	35	^291^
Ni_8_Pt_1_/C	50	0.5	2640.5	39.8	5	100	^292^
Pt_0.7_Ni_0.3_/N-MWCNTs	50	0.004	1595	33.06	5	100	^293^
Ni_0.9_Fe_0.1_-Cr_2_O_3_	70	2.25	82.2	86.3	10	76.5	^294^
Ni–La(OH)_3_/D-MIL-125	70	3.0	870	43.1	20	100	^295^
Rh_47_Ni18P_35_@MOF-74	50	2.0	715.4	49.39	5	83	^296^
Pt_0.6_Ni_0.4_@ZrO_2_/C/rGO	50	0.005	1920	62.3	5	100	^297^
Ni_0.9_Pt_0.1_/MIL-101/rGO	50	1.0	960	50.6	8	100	^298^
Ni–Cr(OH)_3_/C-TiO_2_	70	3.0	910	52.6	20	100	^299^
Ni-CeO_2_@SiO_2_	70	2.0	219.51	59.26	10	70	^300^

To achieve this goal, a variety of metal nanoparticles, including noble metals (Rh, Ir, Pt, and Pd) and non-noble metals (Ni, Fe, Co, and Cu), have been explored to develop efficient catalysts for the selective decomposition of hydrous hydrazine.^280,281^ Among these, bi- and multimetallic catalysts that incorporate Ni alongside noble metals such as Pt, Rh, and Ir have demonstrated effectiveness in this reaction.^282–286^ However, the high cost associated with noble metals limits their widespread application, prompting significant efforts to create efficient noble metal-free catalysts.^287^ Several studies have produced highly selective non-noble metal catalysts like NiFe,^278^ NiCu,^288^ and NiFeCu^289^ although their catalytic activity remains substantially lower than that of their noble metal counterparts.

In addition, it has been observed that using metal oxides—such as Al_2_O_3_,^280^ CeO_2_,^301^ TiO_2_,^279,299,302^ Cr_2_O_3_,^294^ SiO_2_ ^285,300^ and MnO_X_ ^303^ - as supports can significantly enhance both catalytic activity and selectivity. For example, Zhang and colleagues successfully prepared noble-metal-free Cr­(OH)_3_-modified Ni nanoparticles (2.7 nm) supported on defect-rich, carbon-doped mesoporous TiO_2_ nanosheets (C-TiO_2_) through a straightforward and eco-friendly wet-chemistry method (Figure 28).^299^ In this process, the level of oxygen defects in C-TiO_2_ can be precisely regulated by adjusting the amount of carbon doping. Notably, the defect-rich Ni–Cr­(OH)_3_/C-TiO_2_ catalyst achieved 100% selectivity for H_2_, exhibiting extraordinary catalytic activity and remarkable durability for the dehydrogenation of N_2_H_4_·H_2_O, with a turnover frequency (TOF) value of 266 h^–1^ at 323 K.

**Figure 28:**
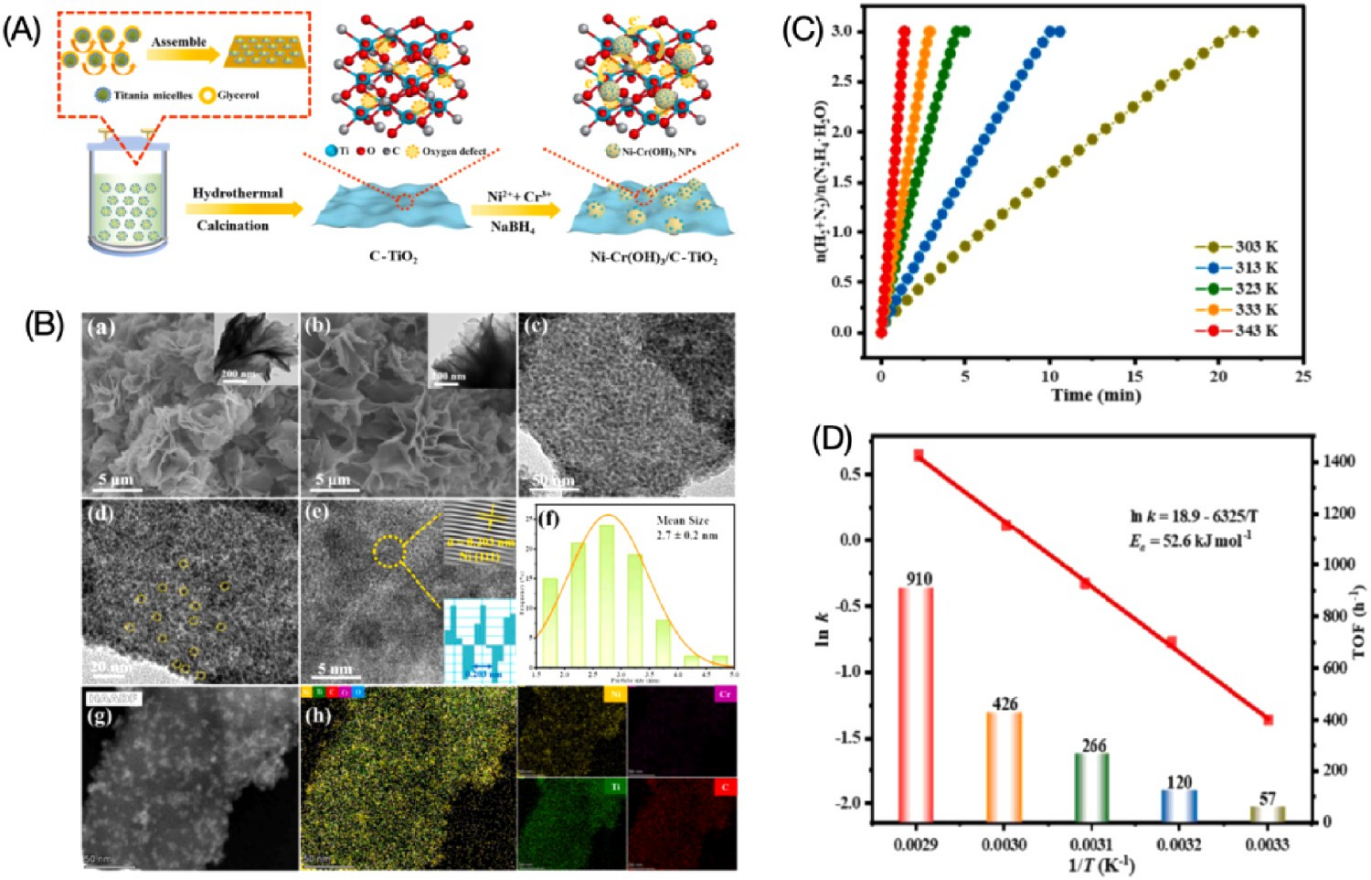
(A) Schematic illustration of the preparation of Ni–Cr­(OH)_3_/C-TiO2; (B) SEM images of (a) C-TiO_2_ and (b) Ni–Cr­(OH)­3/C-TiO_2_ (inset: the corresponding TEM images); (c,d) TEM images, (e) HRTEM image (inset: the corresponding inverse FFT patterns), (f) particle size distribution, and (g) HAADF-STEM image of Ni–Cr­(OH)_3_/C-TiO_2_; (h) The corresponding EDS mapping images of Ni, Cr, Ti, and C; (C) Hydrogen evolution time course plots of N_2_H_4_·H_2_O (0.2 M, 5 mL) at temperatures range of 303–343 K, (D) the related Arrhenius plot (ln k vs 1000/T) and TOF values. Reprinted with permission from ref. 299. Copyright 2020, Elsevier.

In recent years, efforts to enhance catalyst properties have led to the development and use of porous composite supports, such as metal–organic frameworks (MOFs) and graphene oxide (GO), for the immobilization of metal nanoparticles (NPs) in hydrogen production from hydrous hydrazine.^296,297^ For instance, Zou et al. synthesized Ni–Pt NPs using the composite of MIL-101/rGO through a straightforward chemical reduction method (Figure 29).^298^ Analysis of TEM images revealed that the Ni–Pt NPs were uniformly distributed on MIL-101/rGO, likely due to the composite’s distinctive structure and high surface area. The optimized Ni_0.9_Pt_0.1_/MIL-101/rGO nanocomposites demonstrated 100% hydrogen selectivity, achieving a turnover frequency (TOF) of 960.0 h^–1^ for H_2_ production from N_2_H_4_·H_2_O under alkaline conditions at 50 °C.

**Figure 29:**
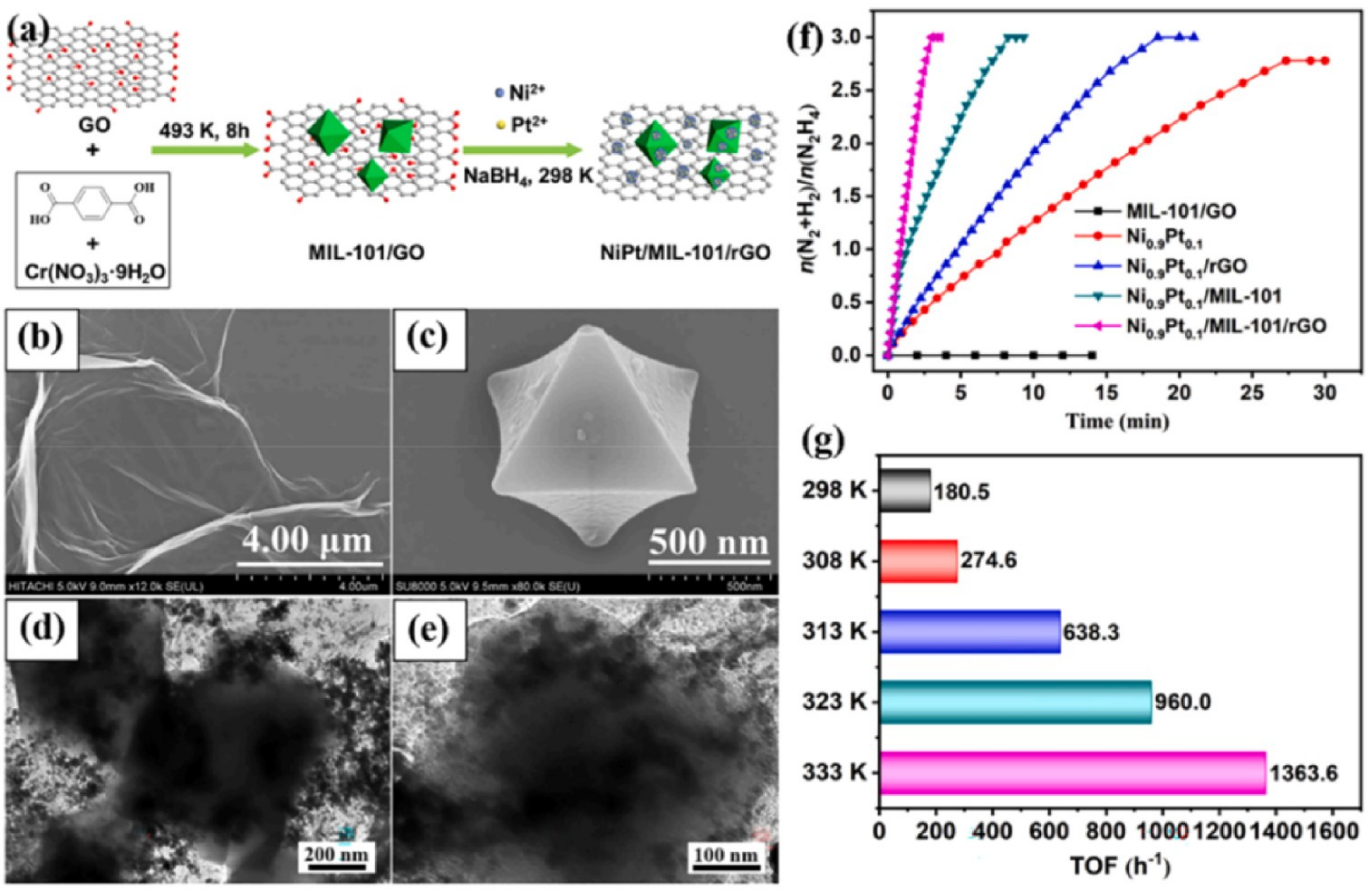
(a) Schematic representation for preparing NiPt/MIL-101/rGO. SEM images of (b) GO and (c) MIL-101. TEM images of (d,e) Ni_0.9_Pt_0.1_/MIL-101/rGO. (f) Gas production curves for HH dehydrogenation with NaOH (1.0 M) over different catalysts at 50 °C (n­(Ni + Pt)/nN_2_H_4_H_2_O = 0.05). (g) TOF values of Ni_0.9_Pt_0.1_/MIL-101/rGO at temperature range from 25 to 60 °C. Reprinted with permission from ref. 298. Copyright 2020, Elsevier.

Defect-engineered metal–organic frameworks (MOFs) have garnered significant interest due to their ability to anchor metal nanoparticles (NPs) and enhance kinetics, improving catalytic efficiency. Long and colleagues developed defective MIL-125-supporting Ni–La­(OH)_3_ NPs using a straightforward, eco-friendly, and cost-effective wet-chemical method (Figure 30).^295^ The content of defects can be easily adjusted by varying the dosage of the reductant. The resulting defective Ni–La­(OH)_3_/MIL-125 catalyst exhibited 100% selectivity for H_2_ and achieved a turnover frequency (TOF) value of 870 h^–1^ for H_2_ production from N_2_H_4_.H_2_O in the presence of NaOH at 70 °C.

**Figure 30:**
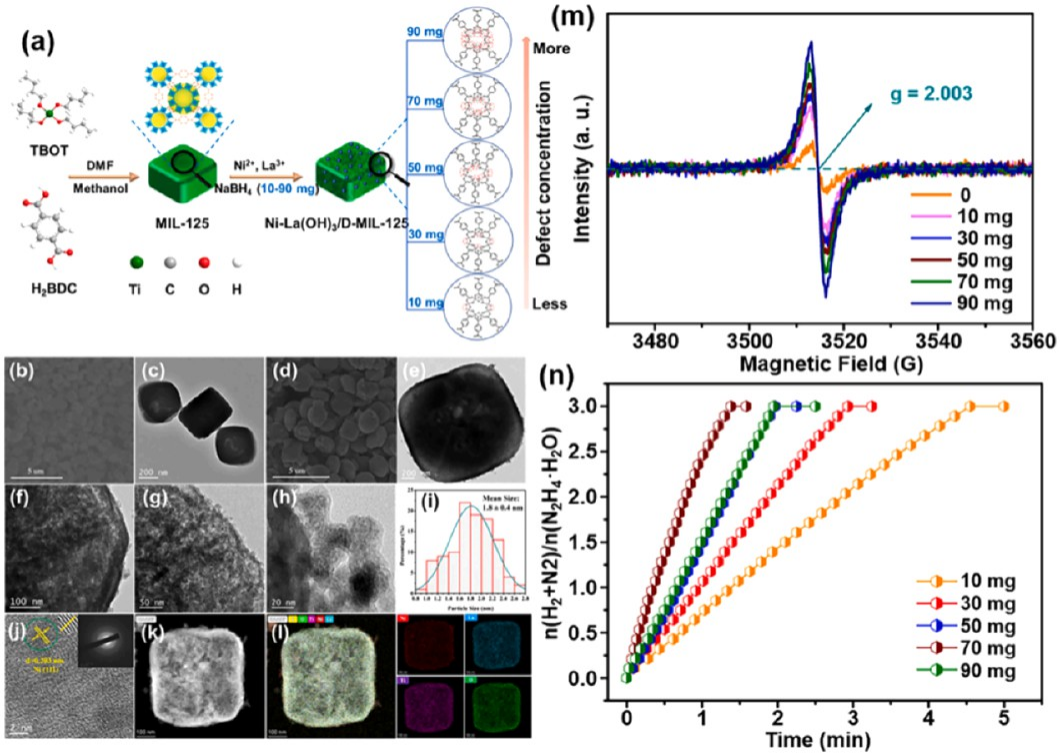
(a) Schematic diagram for preparing Ni–La­(OH)_3_/D-MIL-125 with various NaBH4 amounts. (b) SEM and (c) TEM images of MIL-125. (d) SEM, (e-h) TEM, (i) particle size distribution, (j) HRTEM, (k) HAADF-STEM, and (l) the corresponding EDX mapping images of Ni–La­(OH)_3_/D-MIL-125. (m) Electron paramagnetic resonance (EPR) spectra of Ni–La­(OH)_3_/D-MIL-125 synthesized by various amounts of SB. (n) Gas production curves for N_2_H_4_·H_2_O dehydrogenation (0.4 mol L^–1^, 5 mL) with NaOH (3.0 mol L^–1^) catalyzed by Ni–La­(OH)_3_/D-MIL-125 synthesized with different amounts of NaBH4 at 70 ◦C (nNi/nN_2_H_4_·H_2_O = 0.1). Reprinted with permission from ref. 295. Copyright 2023, Elsevier.

Song and colleagues utilized a wet-chemical method to load ultrasmall Pt–Ni nanoparticles (1.8 nm) onto a ZrO_2_/C/rGO support (Figure 31).^297^ The Pt_0.6_Ni_0.4_@ZrO_2_/C/rGO catalyst exhibited the highest catalytic efficiency, with a turnover frequency (TOF) of 1920 h^–1^ at 50 °C. This significant enhancement in catalytic activity is attributed to the small nanoparticle size, the strong synergistic interactions between Pt and Ni atoms, and the robust interactions between the multisupport ZrO_2_/C/rGO.

**Figure 31:**
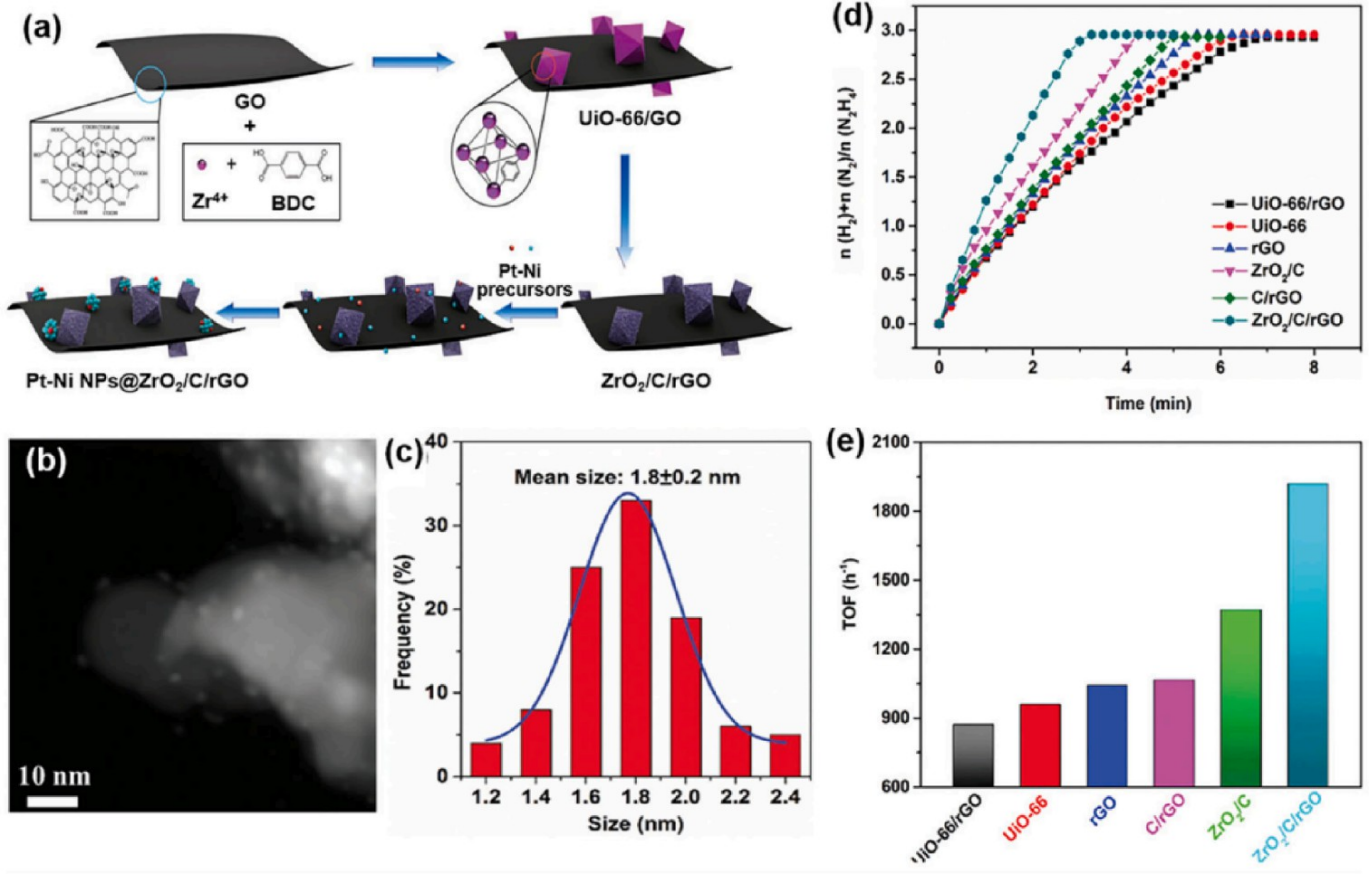
(a) Schematic diagram for preparing PtNi/ZrO_2_/C/rGO. (b) HAADF-STEM image of Pt0.6Ni0.4/ZrO2/C/rGO. (c) Particle size distribution histogram of Pt_0.6_Ni0.4 NPs. (d) Gas production curves and (e) the TOF values for N_2_H_4_·H_2_O dehydrogenation catalyzed by Pt_0.6_Ni_0.4_ NPs on the different supports. Reprinted with permission from ref. 297. Copyright 2020, Wiley.

#### 4.1.3 Releasing Hydrogen from Hydrazine Borane (N_2_H_4_BH_3_, HB)

Hydrazine borane (N_2_H_4_BH_3_, HB) is a promising hydrogen storage material due to its high hydrogen content of 15.4 wt % and notable stability. It can be synthesized by combining hydrazine hemisulfate (H_2_NNH_2_.1/2 H_2_SO_4_) and sodium borohydride (NaBH_4_) in dioxane at room temperature. When appropriate catalysts are utilized, HB can achieve 100% hydrogen utilization efficiency during the hydrolysis of the borane group (eq 59) and the decomposition of the hydrazine moiety (eq 60) in an aqueous solution. This reaction yields a theoretical gravimetric hydrogen storage capacity (GHSC) of 10.0 wt % for the HB-3H_2_O system, significantly surpassing the capacities of the NaBH_4_-4H_2_O (7.3 wt %) and NH_3_BH_3_-4H_2_O (5.9 wt %) systems. Nonetheless, it is important to note that the hydrazine moiety may decompose incompletely, potentially resulting in the production of NH_3_ and N_2_ instead (eq 61).^304,305^

59 $$N_{2}H_{4}{\mathrm{BH}}_{3}{+3H}_{2}O\to N_{2}H_{4}{+H}_{3}{\mathrm{BO}}_{3}{+3H}_{2}$$

60 $$N_{2}H_{4}\to N_{2}{+2H}_{2}$$

61 $${3N}_{2}H_{4}\to {\mathrm{4NH}}_{3}{+N}_{2}$$

To enhance the effectiveness of hydrazine borane as a hydrogen storage material, it is crucial to avoid undesired reaction pathways. As a result, the primary challenge for its practical application lies in developing a highly selective and efficient catalyst for catalytic dehydrogenation under moderate conditions. Table 14 presents a summary of recent findings in the literature.

**Table 14:** Performance of Different Catalysts for HB Dehydrogenation

					Reusability		
Catalysts	Temp. /(°C)	NaOH/(mol L^–1^)	TOF/(h^–1^)	E_a1_ (-BH_3_); E_a2_ (-N_2_H_4_) /(kJ mol^–1^)	Run (No)	Activity/(%)	Ref
NiFe-La(OH)_3_	70	1.5	215.4	27.5; 58.2	5	100	^278^
Ni_0.6_Pt_0.4_/La_2_O_2_CO_3_	25	0.3	1200	34.5; 51.4	5	-	^286^
Ni–La(OH)_3_/D-MIL-125	70	3.0	39.7	20.8; 36.8	20	100	^295^
Ni_0.9_Pt_0.1_/MIL-101/rGO	50	1.0	1578.9	17.6; 56.4	8	48	^298^
Ni-CeO_2_@SiO_2_	70	2.0	442.5	22.65; 58.03	7	70	^300^
Ni_0.6_Pd_0.4_-MoO_*x*_	50	0.75	405.0	49.7; 72.6	5	99	^303^
Co_0.4_Pt_0.6_/CNTs	60	5	6923.6	10.35; 37.80	5	100	^304^
Ni_0.9_Pt_0.1_-MoO_*x*_ /NH_2_–N-rGO	50	1.5	4412	36.1; 41.2	6	100	^305^
Ni_60_Pt_40_/MNC-800	25	2.0	1111	32.2; 50.9	6	100	^306^
Ni_0.22_@Ir_0.78_/OMS-2	50	5.0	2590	40.9; 63.4	5	78	^307^
Ni_0.9_Pt_0.1_-CeO_*x*_/MIL-101	50	1.0	2951.1	10.5; 43.9	6	100	^308^
Rh_0.7_Ru_0.3_-MoO_*x*_/CNTS	30	3.0	4412	6.9; 45.3	15	100	^309^
PtNiNPs/ZIF-8	70	3.0	572.4	19.94; 30.89	5	90	^310^
Co_0.6_Ir_0.4_/TiO_2_	50	4	5625	23.21; 70.85	5	100	^311^
Ni_0.6_Pd_0.4_-MoO_*x*_	50	2.0	405	49.7; 72.6	5	∼100	^312^

Noble-metal-containing nickel-based catalysts, particularly Pt–Ni, are widely acknowledged as the most effective for the complete dehydrogenation of hydrazine borane, largely due to their remarkable alloy synergy effect.^286,298^ However, these catalysts often experience significant aggregation of metal NPs, decreasing the number of active catalytic sites and hampering both activity and stability. To address this issue and enhance the stability of metal NPs, appropriate support materials can be employed to disperse and immobilize these nanoparticles effectively. Moreover, support materials can aid in dispersing the metal NPs and improving their metallic properties through geometric and electronic effects.^308,309^

Huang et al.^300^ successfully developed ultrafine nickel nanoparticles (Ni NPs) that self-assemble onto ceria nanowires (CeO_2_ NWs) and are subsequently encapsulated within a microporous silica shell, designated as Ni-CeO_2_@SiO_2_. This synthesis was accomplished using a straightforward one-pot strategy (Figure 32). The resulting Ni-CeO_2_@SiO_2_ exhibits exceptional performance, achieving 100% hydrogen selectivity for hydrogen production from both hydrazine (N_2_H_4_) and hydrazine borane (N_2_H_4_BH_3_) in an aqueous solution. The remarkable catalytic efficiency of the composite was attributed to the synergistic electronic effects and strong interactions between the Ni NPs and the CeO_2_ NWs, which are rich in oxygen vacancies, as well as its unique structural characteristics.

**Figure 32:**
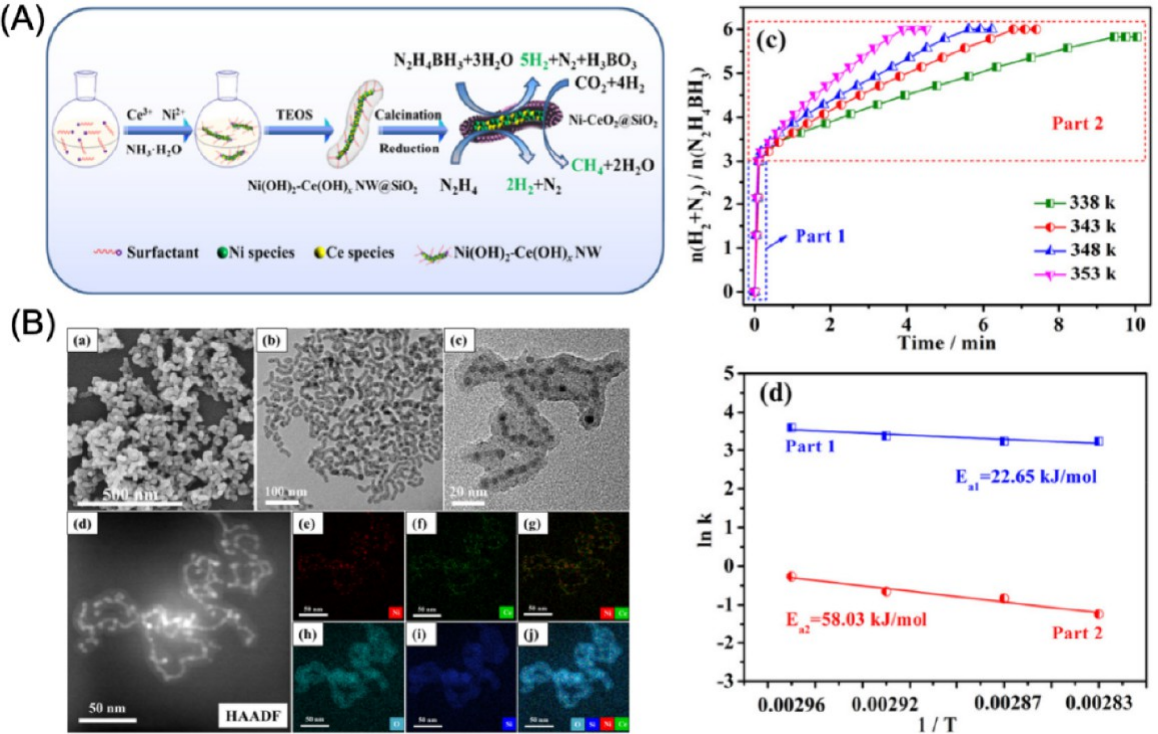
(A) Synthetic scheme for the preparation of Ni-CeO_2_@SiO_2_ catalysts; (B) SEM image, (b, c) TEM images, (d) HAADF-STEM image, and (e–j) EDX mapping of Ni-CeO2@SiO2 catalyst;(c) Time-course plot for gas generation from hydrazine borane aqueous solution (200 mM, 5 mL), and (d) plot of ln k versus 1/T for hydrogen generation from hydrolysis of the BH_3_ group (Part 1) and decomposition of the N2H4 moiety of N2H4BH3 (Part 2) over Ni-CeO2@SiO2 catalyst with (nNi/nHH = 0.1) at different temperatures. Reprinted with permission from ref. 300. Copyright 2020, American Chemical Society.

Bai et al. successfully synthesized highly dispersed ultrafine NiPt-CeO_*x*_ nanoparticles (3.35 nm) supported on MIL-101 through a straightforward wet impregnation reduction method conducted at room temperature.^308^ The resulting Ni_0.9_Pt_0.1_-CeO_*x*_/MIL-101 catalyst demonstrated an impressive conversion rate of 100% and a hydrogen selectivity of 100% at 323 K without surfactants. This catalyst attained a turnover frequency (TOF) value of up to 2951.1 h^–1^ under alkaline conditions. Such exceptional catalytic performance was ascribed to the uniform dispersion and small size of the NiPt-CeO_*x*_ nanoparticles, which are further enhanced by the steric restriction effect of MIL-101, the synergistic interaction between Ni and Pt, and the distinct amorphous/low-crystallinity structure.

Wang et al.^310^ successfully developed a series of monodispersed, ultrasmall, and highly efficient Pt_*x*_Ni_*y*_ alloyed nanoparticles utilizing ZIF-8 as the metal–organic framework (MOF) template (Figure 33). A detailed analysis of the optimal ratios of Pt, Ni, and the support revealed a remarkable synergy characterized by volcano-type interactions between these metals and ZIF-8 support. Experimental results along with DFT simulations suggested that the alloying of Pt with Ni in the PtNi@ZIF-8 structure, which features an optimized surface d-band center, enhances both the absorption and activation of the HB molecule. This optimization effectively reduces the energy barrier for the hydrolysis of the BH_3_ group in HB. Furthermore, a comprehensive comparison of the hydrolytic decomposition processes of HB over the nanocatalysts PtNi@ZIF-8 and RhNi@ZIF-8 indicates that the desorption of the borate is the rate-determining step. In the subsequent decomposition of the N_2_H_4_ moiety, PtNi@ZIF-8 consistently exhibits a smaller Gibbs free energy gap compared to RhNi@ZIF-8, which rationalizes the faster kinetics observed for complete hydrogen evolution.

**Figure 33:**
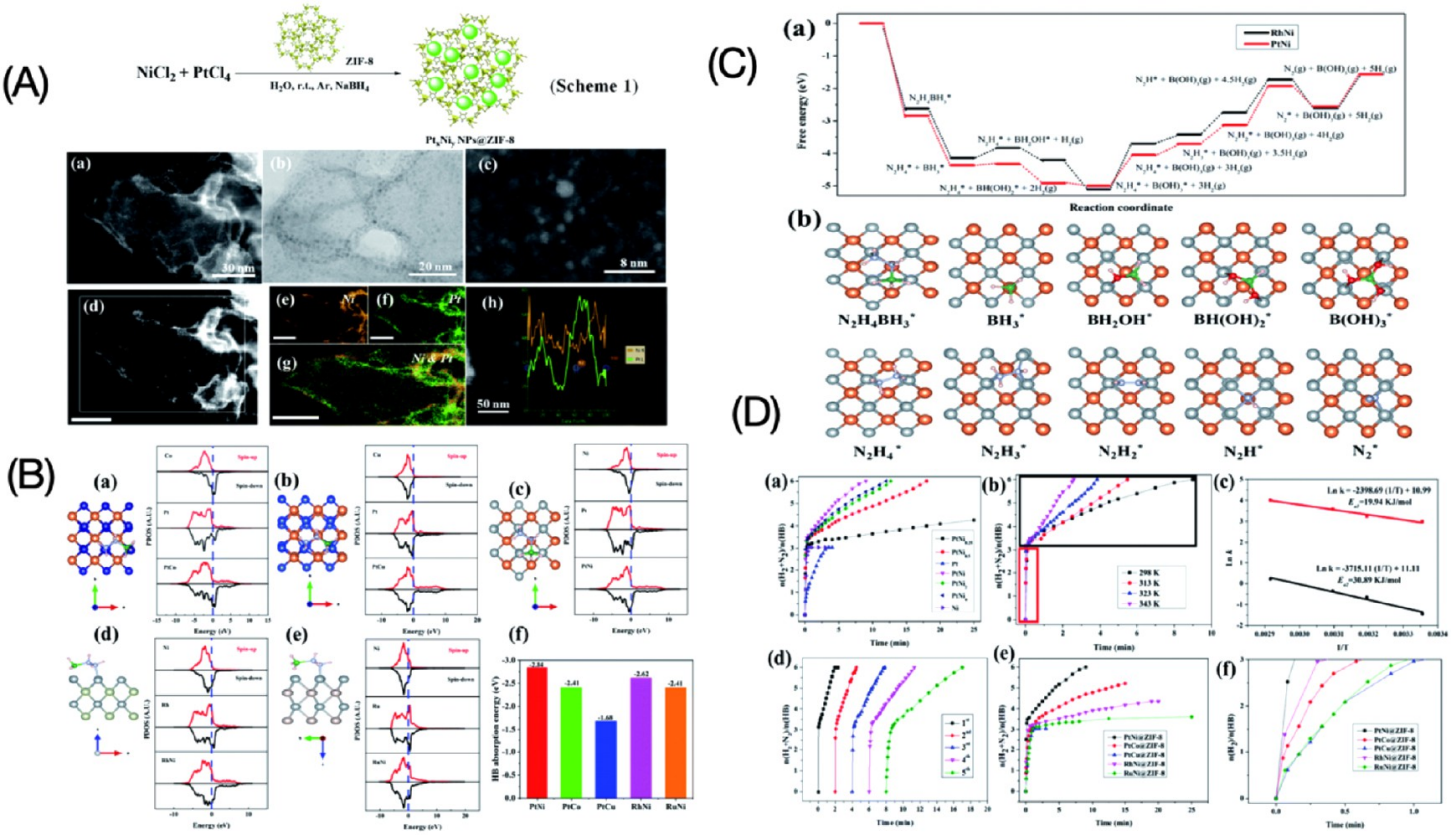
(A) General description of the synthesis of the nanocatalyst and characterization of the PtNi@ZIF-8 nanocatalyst; (a) HAADF-STEM image. (b) TEM image. (c) SE-STEM image. (d-g) HAADF-STEM image and element mapping images (scale bar 50 nm). (h) Line scan shows the PtNi nanoalloys;(B) (a-e) The top view and projected d-orbital DOS of PtCo(001), PtCu(001), PtNi(001), RhNi(001) and RuNi(001), respectively, and the EF is set at 0 eV. (f) DFT-calculated adsorption energies for HB molecules on the (001) of the nanocatalysts. The brown, dark blue, blue, gray, light yellow, golden yellow, green, light red, and light-yellow balls represent Pt, Co, Cu, Ni, Rh, Ru, B, H and N, respectively (C) (a) Calculated Gibbs free energy for H2 generation from the decomposition of HB over the nanocatalysts PtNi@ZIF-8 and RhNi@ZIF-8. (b) Top view of the key intermediates generated from the HB decomposition over PtNi(001). The brown, gray, green, red, light red, and light-yellow balls represent Pt, Ni, B, O, H and N, respectively;(D) (a) Time-course plots for the molar ratio of nH2+N2/nN2H4BH3 from decomposition of HB catalyzed by Pt*_x_* Ni*_y_* @ZIF-8 nanocatalysts. (b) Time-course plots for the decomposition of HB catalyzed by PtNi@ZIF-8 at different temperatures, and (c) its related Arrhenius plots. (d) Recycle test of the PtNi@ZIF-8 nanocatalyst toward the decomposition of HB at 343 K. (e) Time-course plots for the decomposition of HB, and (f) the hydrolysis of BH3 group over PtNi@ZIF-8, PtCo@ZIF-8, PtCu@ZIF-8, RhNi@ZIF-8 and RuNi@ZIF-8. Reprinted with permission from ref. 310. Copyright Creative Commons Attribution 3.0 Unported License 2022, Royal Society of Chemistry.

## 5 Computational Methods for Catalyst Design

At its core, catalyst design can be viewed as an optimization problem.^313^ By employing modern techniques such as Density Functional Theory (DFT) in conjunction with Artificial Intelligence (AI) and Machine Learning (ML), research groups can enhance the efficiency and selectivity of catalysts, while also accelerating results and minimizing capital loss.^314^ Through the application of modern computational methods, researchers have shown, for example, that the bond strength between a substrate and a catalyst can be accurately assessed using the d-band center of the metallic components of the catalyst. Moreover, they have identified that the linear relationship between activation energy and the enthalpy change of an elementary step—referred to as the Brønsted–Evans–Polanyi (BEP) relation—exemplifies how an effective catalyst must balance interactions with the adsorbate, avoiding extremes of either too weak or too strong bonds, giving rise to the well-known volcano curve.^313^

### 5.1 Density Functional Theory (DFT): A Powerful Tool for Understanding Catalytic Activity

Since its proposal, grounded in the Hohenberg–Kohn theorems, Density Functional Theory (DFT) has provided a robust framework for developing computational strategies that have revolutionized the field of quantum chemistry. It offers valuable insights into the energetics, structure, and properties of atoms and molecules at a significantly reduced cost compared to traditional ab initio wave function methods.^315,316^

In many electron systems, the interactions among electrons give rise to a many-body problem. The foundation of density functional theory (DFT) consists of using electronic density as a surrogate to describe the physics governing electron interactions. DFT has been successfully used to predict the optical, optoelectronic, catalytic, and magnetic properties of different materials, with results aligning closely with experimentally determined values.^317,318^ Helping to identify the properties responsible for the activity and selectivity of a specific catalyst, enables the exploration of new catalyst leads.^319^

#### 5.1.1 Understanding Catalyst Activity through Electronic Structure Calculations

Li et al.^320^ used DFT to investigate the influence of nonmetals (NM) (C, N, O, P, S, Se) on the stability, electronic properties and electrocatalytic activity of the composites formed by precious metals (M) (Ru, Rh, Pd, Ag, Os, Ir, Pt, Au) adsorbed on silicene (a silicon allotrope), giving a total of 48 composites with the general structure Si-NM_2_-M. The electronic calculations, particularly the determination of the binding energies, indicated that doping has a beneficial effect on stabilizing the composites. O_2_-doped systems were found to be the most stable among the composites. Analyzing the electronic properties of the 15 most stable composites revealed that varying the elements significantly influences the electronic configuration of the composites. In Si–O_2_–Ru, Si–O_2_–Rh, and Si–O2–Ir-doped systems, for example, the metal atoms combine covalent and ionic bonds with Si atoms. In contrast, the bonds in the other composites are purely ionic. The conductivity properties of the materials were also significantly impacted: Si–O_2_–Ru, Si–N_2_–Os, Si–P_2_–Os, and Si–O_2_–Pt exhibited semiconductor properties with narrow energy gaps, while the other composites exhibited metallic properties. The magnetic properties varied widely: Si–O_2_–Rh, Si–S_2_–Ir, and Si–Se_2_–Ir are magnetic, whereas the other composites have no magnetic properties.

The different combinations of precious metal and nonmetal dopants made it possible to predict different activities in optimizing the hydrogen evolution reaction (HER) and the oxygen evolution reaction (OER). Studying both reactions is important since the OER in the anode is slow, interfering with the whole water electrolysis process. The catalytic activity was predicted considering the adsorption free energies for the intermediates in the hydrogen evolution reaction (HER) and the oxygen evolution reaction (OER) (Figure 34). In particular, the composite doped with S, Si–S_2_-Ir, showed a strong performance in the hydrogen evolution reaction (HER) at 300 K, while the system doped with N, Si–N_2_-Pt, showed excellent activity in the oxygen evolution reaction (OER). The results suggest that doping with nonmetals effectively improves the stability and electrocatalytic water splitting by the precious metal/silicene composite.

**Figure 34:**
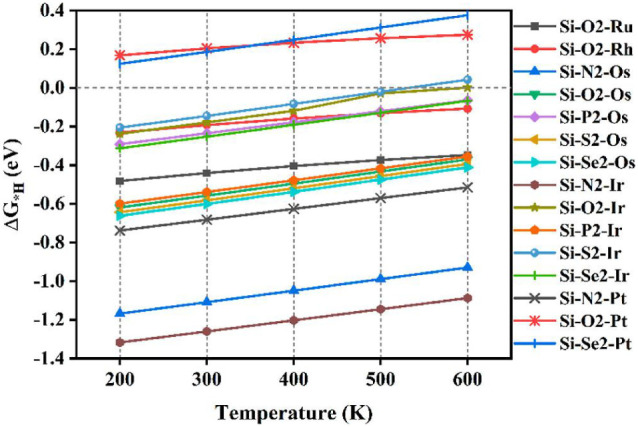
Free-energy changes of HER intermediates (*H) at temperatures between 200 and 600 K. Reprinted with permission from ref. 320. Copyright Creative Commons Attribution-NonCommercial-NoDerivatives 4.0 International (CC-BY-NC-ND 4.0) 2022, American Chemical Society.

Wu et al.^321^ studied the characteristics of Co_n_MoP clusters through DFT calculations using a B3LYP hybrid functional basis. Co–Mo–P systems have shown potential as electrocatalysts in the hydrogen evolution reaction. One possible reason could lie in the interconnection between their magnetic properties, particularly due to d orbitals electrons, and hydrogen adsorption, a key step in the hydrogen evolution mechanism. The study aimed to establish a theoretical basis for synthesizing potential alternatives to the expensive traditional Pt catalysts by examining the magnetism and electronic features of Co_n_MoP under different numbers of metal atoms and different geometries. The ConMoP clusters (n = 1 ∼ 5) underwent optimization calculations and vibrational analysis at the def2-tzvp quantization level, accounting for relativistic effects in elements beyond the fifth period, identifying 16 stable configurations. The authors studied bond lengths, bond angles, singleton counts, spin Bouygues number, magnetic moments, density of states, electron spin-density difference maps, localized orbital analysis (LOL), orbital delocalization, and the infrared spectra for the stable configurations. Their findings indicated Co d orbitals are the main factors influencing the magnetic properties of the Co_n_MoP, while Mo and P atoms negatively affect the magnetism. Notably, the Co_3_MoP cluster displayed the highest magnetic properties.

#### 5.1.2 Mapping Reaction Pathways: DFT Analysis of Surface Interactions

Computational catalyst design has proven essential in addressing the specific kinetic challenges associated with hydrogen evolution reaction (HER) electrocatalysts, particularly in optimizing the balance between water dissociation and hydrogen desorption.^322^ Li et al.^322^ employed DFT ab initio calculations to tackle the sluggish water dissociation kinetics that limit ruthenium (Ru), a cost-effective alternative to platinum. Although Ru demonstrates an optimal hydrogen binding energy (HBE) comparable to that of Pt, its catalytic performance in alkaline media is restricted due to weak water adsorption, which arises from unfavorable orbital interactions. Through theoretical modeling of a Ru/MoO_2_ composite, it was found that engineering specific Ru–O–Mo interfacial sites facilitates a significant charge transfer (1.81 e^–^) from Ru to the oxide support, thereby notably modulating the electronic structure. This charge redistribution enhances water adsorption on Mo sites (2.06 eV) compared to pure Ru (0.35 eV), while still maintaining favorable hydrogen adsorption on Ru sites. As a result, this approach effectively reduces the energy barrier for the rate-determining Volmer step (water dissociation) (Figure 35).^322^

**Figure 35:**
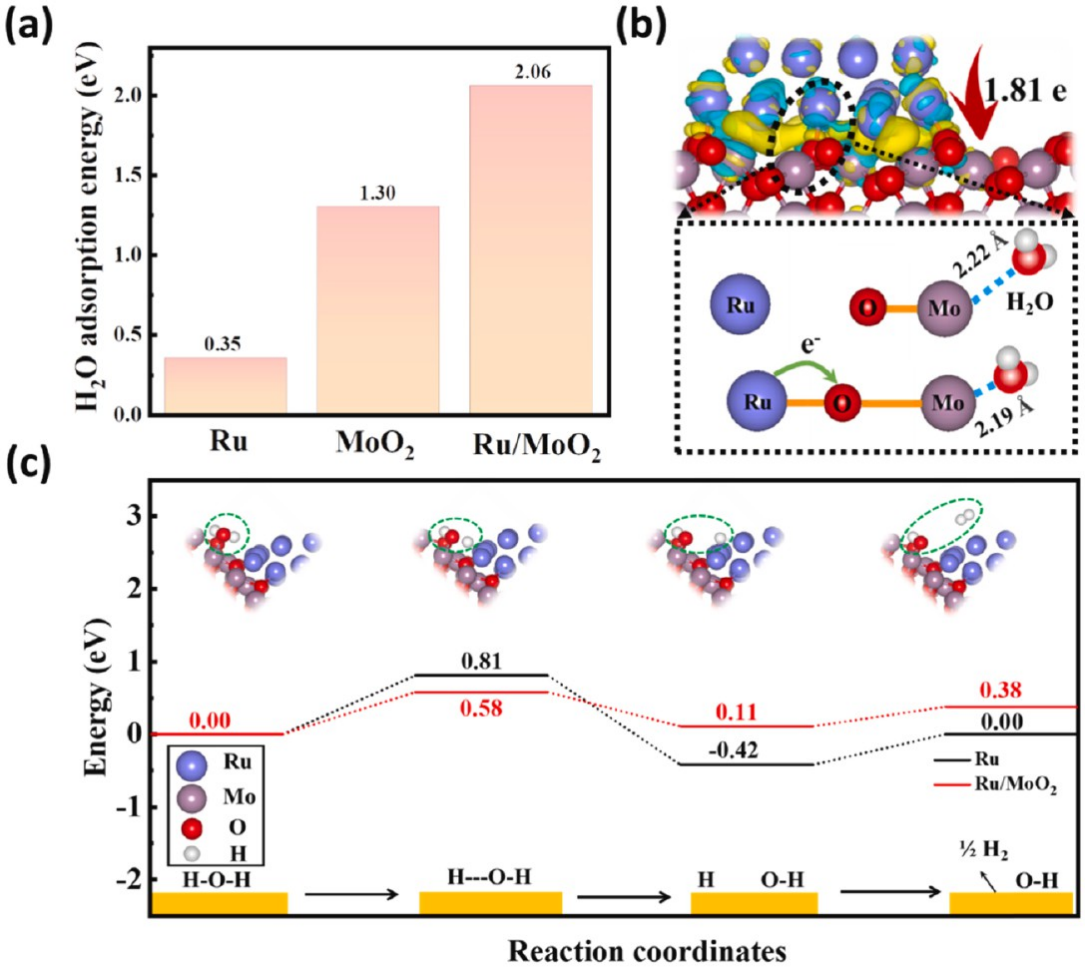
Design principles and DFT calculations of catalysts. (a) Calculated H_2_O adsorption energy for Ru, MoO_2_, and Ru/MoO_2_. (b) Charge density difference, Bader charge transfer at the interface of Ru/MoO_2_, and the schematic diagram of charge transfer affecting H_2_O adsorption on the Ru–O–Mo interface sites (yellow and blue regions represent electron accumulation and depletion, respectively). (c) Calculated free-energy diagrams of H_2_O reduction to H_2_ on the surface Ru and Ru/MoO_2_ catalysts. Reprinted with permission from ref. 322. Copyright 2021, Elsevier.

Validating these theoretical predictions, the experimental synthesis of the Ru/MoO_2_ catalyst confirmed that altering the local coordination environment directly enhances kinetic performance and stability. The synthesized catalyst, comprising 21.26 wt % Ru, achieved an impressively low overpotential of 16 mV at 10 mA cm^–2^, surpassing both pure Ru, which exhibited an overpotential of 59 mV, and commercial Pt at 31 mV. Notably, Tafel slope analysis indicated a shift in the reaction mechanism: the low slope of 32 mV dec^–1^ for the composite, in contrast to 174 mV dec^–1^ for pure Ru, suggests that the rate-determining step transitioned from the challenging water dissociation (Volmer step) to the more rapid hydrogen desorption (Heyrovsky/Tafel steps). Furthermore, the robust interaction between the metal and the support contributed to exceptional durability, with the catalyst maintaining stability for over 40 h without the degradation commonly seen in non-noble or hybrid systems, thereby confirming the reproducibility of the charge transfer effects predicted by XANES and XPS analysis.^322^

In addition to optimizing specific composites, computational screening is increasingly utilized to identify novel, earth-abundant single-atom catalysts (SACs) that align with the Sabatier principle for ideal thermodynamic conditions. Sarfaraz, Yar, and Ayub conducted high-throughput density functional theory (DFT) screening on metallofullerenes (M@C_60_), focusing on materials that achieve a near-zero Gibbs free energy of hydrogen adsorption (|ΔG_H_|≈0). This systematic evaluation of first-row transition metals highlighted Fe@C_60_ as a standout candidate, exhibiting a ΔG_H_ of 0.08 eV, suggesting an optimal thermodynamic balance between adsorption and desorption rates. The resulting volcano plot positions Fe@C_60_ at a thermoneutral apex, comparable to platinum, illustrating that encapsulating earth-abundant metals within carbon cages can effectively adjust the d-band center to mimic the activity of noble metals (Figure 36). This discovery presents a promising pathway toward scalable and environmentally sustainable hydrogen production.^323^

**Figure 36:**
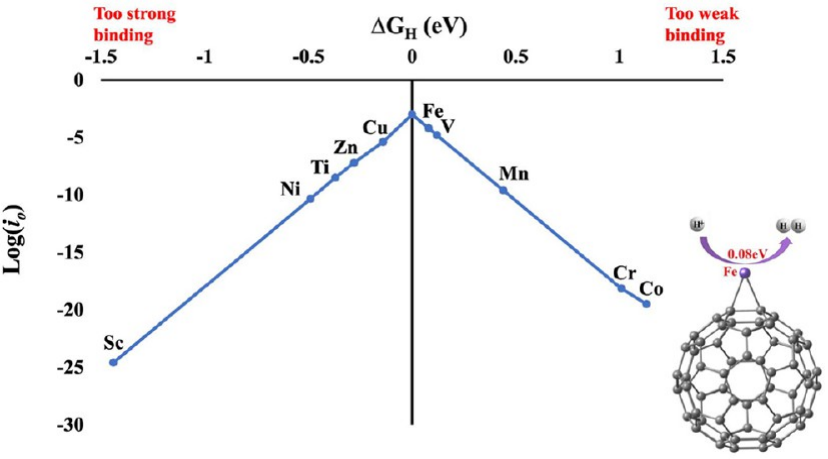
2-D volcano plot for forming HM@C60 complexes in the HER (simple kinetic model). Reprinted with permission from ref. 323. Copyright 2024, Elsevier.

#### 5.1.3 Determining Reaction Mechanisms: DFT Calculations of Energy Barriers

Computational methodologies, particularly Density Functional Theory (DFT), have become indispensable for bridging the gap between the intrinsic reactivity of finite noble metal clusters and the stability requirements of scalable intermetallic surfaces. For example, Camacho-Mendoza and Cruz-Borbolla employed DFT (B3LYP/6–31G**) to evaluate the energetic feasibility of formic acid dehydrogenation on finite Pd_4_ clusters, providing a proof-of-concept for highly reactive subnanometer geometries. Their simulations demonstrated that these symmetrical clusters promote hydrogen production through favorable thermodynamic conditions (ΔG = −11.45 kcal mol-1) and low energy barriers (under 30 kcal mol^–1^) (Figure 37).^324^ However, despite the high intrinsic activity of such noble metal systems, supported by weak noncovalent interactions, their industrial applications, as already mentioned, are often hindered by the high cost of palladium and potential stability issues under operating conditions.

**Figure 37:**
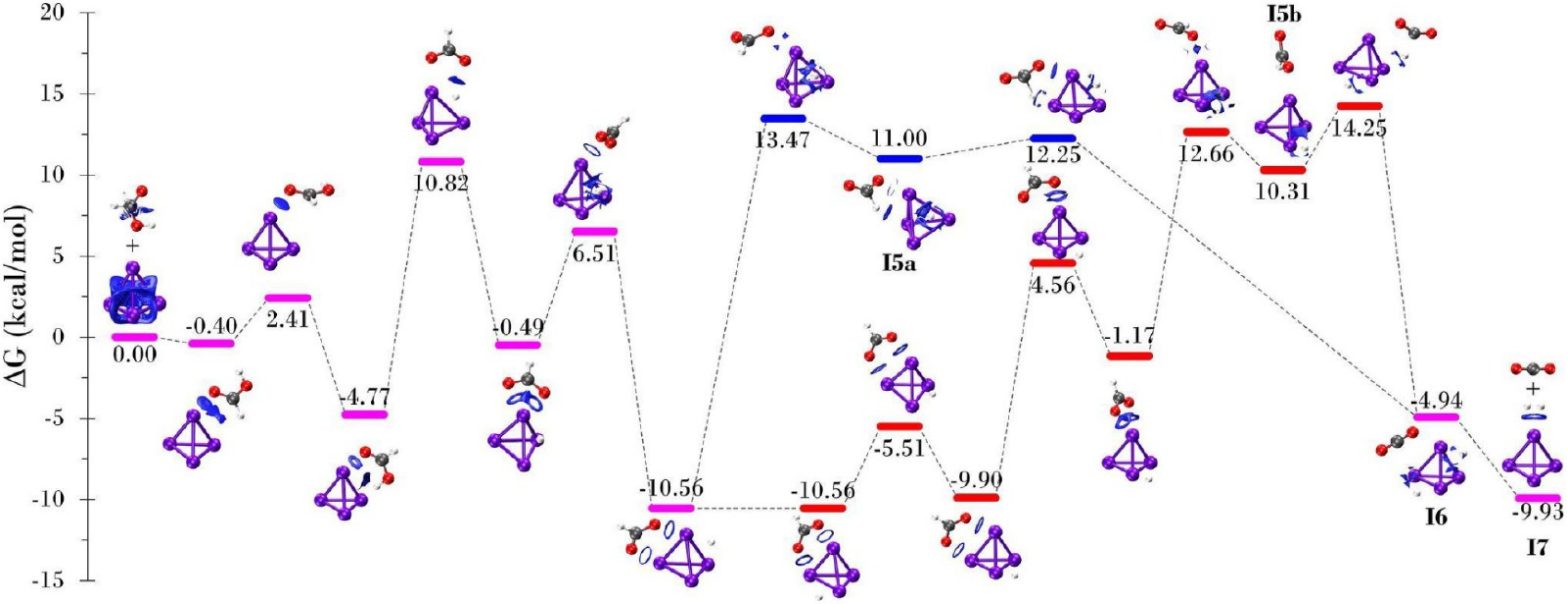
Total reaction profile of hydrogen production from Pd_4_ cluster. Calculations were made by Gaussian 9 software using DFT theory. ΔG and RDG isosurface graph at 0.5 eV, with the color blue indicating the contacts present. Reprinted with permission from ref. 324. Copyright 2020, Elsevier.

To address the pressing need for cost-effective and durable alternatives, Mauri et al. explored Ni_3_Sn_4_ intermetallic compounds for methanol decomposition, integrating DFT with operando X-ray spectroscopy to compare theoretical predictions with experimental surface dynamics. In contrast to the static active sites modeled in pure metal clusters, this study revealed a dynamic protection mechanism wherein surface tin-oxide phases inhibit the deactivation of nickel atoms, a common failure mode in non-noble catalysts. This surface reconstruction not only enhances durability but also increases selectivity for H_2_ over secondary products such as CO_2_.^325^ The comparison of these systems highlights a unifying theme in rational catalyst design: transitioning from optimizing intrinsic kinetics in costly noble metal clusters to engineering robust, self-protecting active sites in earth-abundant intermetallics (Figure 38).

**Figure 38:**
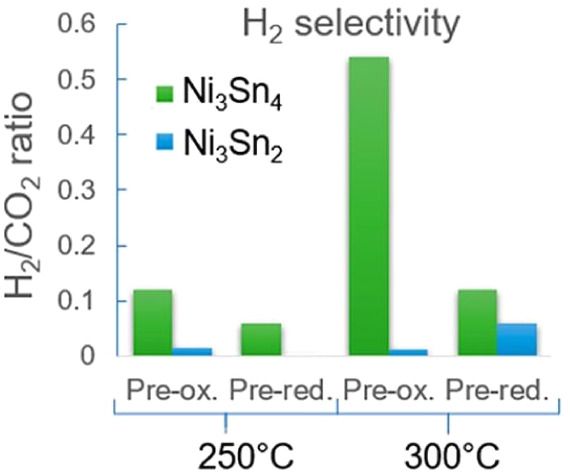
Online micro gas chromatography results obtained by analyzing the gas products from the decomposition of CH_3_OH catalyzed by Ni_3_Sn_4_ and Ni_3_Sn_2_, following sample preoxidation and prereduction at temperatures of 250 and 300 °C. Reprinted with permission from ref. 325. Copyright 2023, American Chemical Society.

### 5.2 AI and Machine Learning: Revolutionizing Catalyst Discovery for Hydrogen Production

Artificial Intelligence (AI) is broadly acknowledged for its goal of creating intelligent machines and programs that demonstrate a higher level of automation, simulating human intelligence to carry out specific tasks. The algorithms and models utilized in AI encompass diverse techniques, including artificial neural networks, machine learning (ML), support vector regression, and fuzzy logic models.^326^ For example, machine learning has gained widespread application in research on developing new catalysts. Advances in materials research have indeed yielded significant volumes of high-quality data. Incorporating machine learning (ML) in materials design and discovery is a logical response to identifying specific materials from a vast set of options.^327^

Machine learning refers to the capability of computers to recognize and analyze patterns autonomously, without explicit supervision from humans. The primary objective of machine learning is to devise algorithms that can learn from data independently and identify trends to predict potential outcomes.^314^ To date, ML has facilitated the development of precise data-driven models that helped establish critical relationships between the characteristics of materials and their targeted catalytic performance, including activity, selectivity, and stability.^328^

There are two primary techniques in machine learning: supervised learning and unsupervised learning. Supervised learning focuses on making future predictions based on known data inputs—such as surface topology, elastic modulus, particle size or morphology, Pauling electronegativity, atomic coordination environment, atomic volume, and formation energies—and their corresponding target outputs, which may include catalyst selectivity, conversion rates, and catalyst stability. The resulting function is then applied to predict output for a given input, and these predictions are subsequently compared to experimental outcomes. In contrast, unsupervised learning analyzes unlabeled data to uncover unknown patterns, such as clustering materials into distinct groups or identifying outliers within a data set. This technique visualizes the existing chemical space and investigates the underlying distributions of data sets without necessitating target values.^314,327,328^

#### 5.2.1 Predictive Modeling and Data-Driven Discovery

In their 2022 study, Kim, Won, and Kim^329^ developed a machine learning model particularly useful in chemical engineering, aimed at predicting catalyst performance for the water–gas shift reaction (WGSR) in hydrogen production without requiring detailed kinetic data or rigorous process modeling. The schematics presented in Figure 39 shows how their approach would simplify the process of finding the best catalyst.

**Figure 39:**
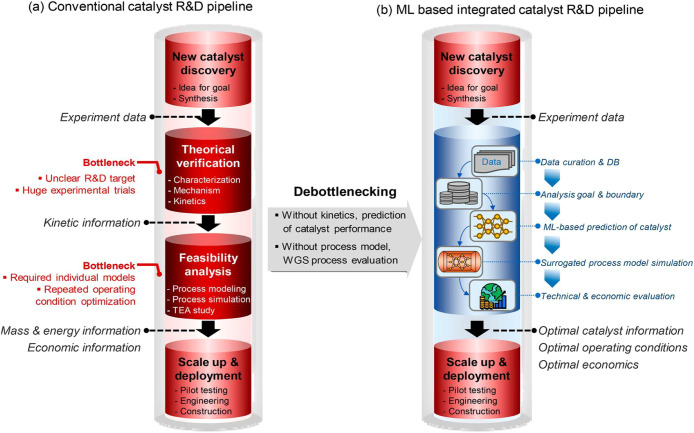
Schematics of (a) conventional catalyst R&D and (b) proposed ML-based catalyst R&D framework. Reprinted with permission from ref. 329. Copyright 2022, American Chemical Society.

The authors based their predictions on data collected from the literature, analyzing a total of 419 data sets focused on Pt catalysts, featuring combinations of (i) promoters such as Ce, Co, Fe, Mn, Ni, Y, La, Gd, Ti, Cr, Ho, Nd, Tm, Sm, or Er, and (ii) supports including Al_2_O_3_, CeO_2_, TiO_2_, Y_2_O_3_, La_2_O_3_, Co_3_O_4_, ThO_2_, and Fe_2_O_3_. The input variables (descriptors) were treated as continuous variables in the artificial neural networks (ANNs), except for the support, which was categorized as a binary integer variable. They selected resilient back-propagation and the logistic sigmoid function as learning algorithms. The work was funneled on the one-pass CO conversion of 30 types of platinum (Pt) catalysts utilizing a standard gas composition of 3 vol % CO and 10 vol % H_2_O. Ultimately, the study evaluated the hydrogen production process using a range of technical and economic metrics, such as the volume of hydrogen generated, energy consumption, and unit energy cost. The findings indicated that the processes involving Pt/Co (10 wt %)/Al_2_O_3_, Pt/Co (20 wt %)/Al_2_O_3_, and Pt/Ce­(5 wt %)/TiO_2_ demonstrated the highest performance in producing high-purity hydrogen.^329^

Yang et al.^330^ employed machine learning to investigate combinations of donor–acceptor–acceptor (DAA) and donor–donor–acceptor (DDA) organic ternary heterojunction photocatalysts (TOHP) for hydrogen production via photocatalysis. Given the vast diversity of organic molecules suitable for combination, the corresponding resource and experimental requirements could become prohibitive. To pinpoint TOHPs with enhanced performance, the researchers encoded 864 ternary combinations derived from 12 donors and 8 acceptors. They then utilized machine learning techniques to traverse this chemical space. The chemical descriptors incorporated into their model included the ionization potential of the components (associated with the HOMO), energy affinity (linked to the LUMO), and both hole and electron reorganization energies. The experimental data used to train the model encompassed 104 TOHPs, which exhibited hydrogen evolution rates (HERs) ranging from zero to 737.4 mmol g^–1^ h^–1^. The study covered 17% of the total combinatorial space, evaluating just 736 out of 4320 potential experiments. The ten most active TOHPs identified through this approach showcased impressive sacrificial hydrogen production rates (using ascorbic acid as the sacrificial agent), exceeding 500 mmol g^–1^ h^–1^, with the most effective ternary material achieving a remarkable rate of 749.8 mmol g^–1^ h^–1^ under 1 kW m^–2^ (1 sun illumination), among the highest reported for organic photocatalysts in the literature.

#### 5.2.2 Fine-Tuning Catalysts: AI-Driven Optimization Strategies

Not only the type of catalyst employed but also its distribution within the reactor are essential factors in hydrogen production. Pajak et al.^331^ applied a genetic algorithm for multiobjective optimization, targeting the maximization of methane conversion rates in the steam reforming reaction while minimizing the temperature differential between the highest and lowest points in a small-scale methane/steam reforming reactor. To enhance computational efficiency, the study utilized the concept of macro-patterning, which involves segmenting the reformer’s reactor into distinct sections, filled either with noncatalytic metallic foam or Ni/YSZ (Nickel/Yttria-stabilized zirconia). The optimization process utilized a genetic algorithm to adjust the composition and porosity of these segments, aiming to improve CH_4_ conversion rates and minimize temperature gradients. Each reactor’ section could contain either a catalytic material or noncatalytic metallic foam, with defined porosity and pore size. The impact of porosity and pore size on the active reaction surface and permeability was modeled using graph theory and three-dimensional digital material representation.

After processing through 50 generations, a solution demonstrating enhanced thermal conditions was achieved. The algorithm determined that only 33% of the reference amount of the catalytic material was necessary. Despite this significant reduction in Ni/YSZ quantity, the CH_4_ conversion rate decreased by only 22%. This outcome confirmed that improvements in the reactor’s thermal conditions could elevate the reforming process’s effectiveness. However, the authors acknowledged that further algorithm development is needed to identify the global optimum.^331^

Esfandiary, Karimi, and Saedodin^332^ utilized machine learning (ML) to optimize the catalyst coating pattern in a microreactor aimed at maximizing the total hydrogen (H2) production rate during the Steam Methane Reforming (SMR) process. They implemented a multiobjective optimization approach using Nondominated Sorting Genetic Algorithm (NSGA-II) surrogate models, developed from extensive data sets derived from computational fluid dynamics (CFD) and machine learning. These surrogate-based optimization methods capitalize on data from high-fidelity models to approximate design parameters and optimization objectives through specific functions. The functions underwent thorough validation, achieving an impressive accuracy rate of 99.9%. The results from the optimal Pareto set (optimal solutions) provided by NSGA-II indicated that the use of an optimized coating could improve the H_2_ production rate per coated area by 65.8% compared to an otherwise fully coated channel.^332^

### 5.3 Synergy between DFT and AI/ML

DFT and AI/ML can be combined to enhance the search for better catalysts. While DFT provides detailed atomic-level insights, AI/ML contributes with rapid data analysis and predictive capabilities. DFT can generate data that serves as input for machine learning models, and conversely, machine learning can help identify promising candidates for further investigation through DFT (Figure 40). This synergistic approach accelerates the discovery cycle, ultimately reducing the time and cost associated with catalyst development.^333^

**Figure 40:**
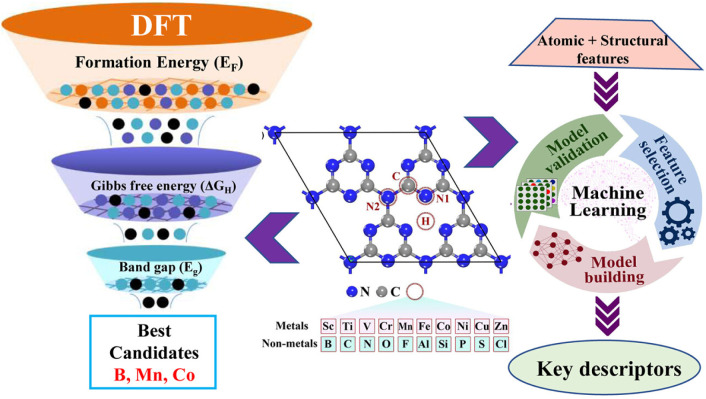
Schematics of the integration of DFT and ML in the discovery of new catalysts. Reprinted with permission from ref. 334. Copyright 2023, American Chemical Society.

Jyothirmai et al.^334^ screened various metals (Al, Sc, Ti, V, Cr, Mn, Fe, Ni, Cu, Zn) and nonmetals (B, C, N, O, F, Si, P, S, Cl) as single-atom catalysts (SACs) embedded in different active sites of graphitic carbon nitride (SACs@g-C_3_N_4_). Their analysis utilized first-principles DFT calculations and six machine-learning algorithms to identify the most effective catalyst for hydrogen production via electrolysis. Among the 80 configurations studied, 18 candidates (including Sc, Ti, Mn, B, Al, and Si at the C-site; Sc, Mn, Fe, Co, Ni, B, O, Al, and Si at the N1-site; B and P at the N2-site; and B at the H-site) exhibited negative formation energy, indicating their structural stability and potential for successful synthesis. The support vector regression (SVR) model, one of the six algorithms applied, identified the formation energy, bond length, boiling point, melting point, and valence electron count as the key descriptors related to the hydrogen evolution reaction. The ΔGH values highlighted the superior HER performance of B@N1, Mn@N1, and Co@N1 configurations, attributed to their moderate hydrogen adsorption, suggesting they may outperform Pt (111) in catalytic efficiency.^334^

Liu et al.^335^ also conducted a study on the hydrogen evolution reaction (HER) utilizing single-atom catalysts (SACs), this time doping into two-dimensional GaPS_4_. They assessed the thermodynamic stability of single-atom doping at various defect sites within the GaPS_4_ lattice through DFT calculations. The HER performance was evaluated using an implicit solvation model, with feature importance determined via a gradient-boosted regression approach using ML. Their ML regression analysis revealed a strong correlation between electron affinity and first ionization energy as key descriptors influencing adsorption characteristics, underscoring their significant impact on HER performance. Notably, the incorporation of transition metals significantly improved hydrogen atom adsorption and reduced the overpotential for SAC@VS1-GaPS_4_ (SACs = Fe, Ni, Ru, Pt) and SAC@VS2-GaPS_4_ (SACs = Os, Ni, Au, V), where VS1 and VS2 denote distinct defect sites within the GaPS_4_ structure.

## 6 Overview

The period spanning from 2020 to 2025 has witnessed significant shifts in the focus of catalytic research for hydrogen production, propelled by the global push for sustainability and cost-effective solutions. While traditional methods such as steam methane reforming (SMR) continue to dominate, there is a clear trend toward developing catalysts that mitigate the environmental and economic drawbacks associated with these methods, including issues like coking, sulfur poisoning, and high energy consumption.

A prominent theme across various production techniques is the emphasis on cost-effective and durable materials. Researchers are increasingly investigating noble-metal-free or low-noble-metal catalysts to reduce costs without sacrificing performance. For instance, in both electrocatalytic and chemical hydride systems, non-noble metals such as nickel, cobalt, and iron, often utilized in bimetallic or trimetallic alloys, are under extensive examination and refinement.

Another noteworthy trend is the emergence of bioinspired and hybrid systems that integrate biological and synthetic components. Instead of relying solely on microorganisms, recent advancements have incorporated biocomponents, such as algae or bacteria, alongside conductive nanomaterials or catalysts. This approach seeks to address the inherent limitations of biological systems, such as slow reaction kinetics and oxygen sensitivity, by developing robust, high-performing hybrid systems.

Lastly, computational methods have evolved from merely auxiliary tools to become an essential element of the discovery pipeline. Techniques such as Density Functional Theory (DFT) and Machine Learning (ML) are now employed to screen thousands of potential catalyst candidates, predict their properties, and identify optimal compositions and structures. This synergy between theoretical modeling and experimental efforts is accelerating the development of innovative catalysts with enhanced activity and stability, marking a fundamental shift in the design and optimization of new materials.

Table 15 provides an overview of the catalysts that were addressed throughout this review (sections 3 to 5).

**Table 15:** Overview of Catalysts Type by Method

H_2_Production Route	Catalyst commonly utilized	Typical Performance Metrics	Key Recent Advances	Main Challenges
Steam Methane Reforming (SMR)	Ni-based alloys, noble metals	Methane conversion %, TOF	CaO-based sorbent-enhanced SMR, bimetallic Ni–Co alloys	Coking, sulfur poisoning, sintering
Water Electrolysis	Pt-group metals, Mo, Ni-based	Overpotential, Tafel slope	MoO_2_@CoMo heterostructures, single-atom catalysts (SACs)	High overpotential, stability
Photocatalysis	TiO2, CdS, organic semiconductors	H_2_ evolution rate, AQE	MOF hybrids, g-C3N4, metal-free COFs	Carrier recombination, visible-light absorption
Biological/Hybrid	Bionano systems, hydrogenases	Hydrogen production rate	Algae-nanoparticle hybrids, DIET enhancement	Oxygen sensitivity, kinetics
Chemical Hydrides	Pt, Ru-based NPs, Ni alloys	TOF, activation energy	RuPt tandem catalysts, NiPt@ZIF-8	Selectivity, catalyst aggregation

### 6.1 Comparative Techno-Economics of Hydrogen Pathways

To contextualize the significant catalytic advancements, it is essential to compare the economic viability of these routes using cost estimates. Table 16 compiles the relevant information available in the literature. This comparison underscores that although green hydrogen represents the ultimate goal, blue hydrogen (SMR+CCS) serves as an indispensable transitional technology, highlighting the ongoing requirement for advanced SMR catalysts.^336^

**Table 16:** Economic Viability of Different Hydrogen Production Routes

Pathway	Est. Cost ($/kg H2)	Primary Cost Driver	2025 Trend	References
**SMR (Gray)**	$1.50 – $2.50	Natural Gas Price	Cost rising with carbon taxes.	^336^
**SMR + CCS (Blue)**	$2.00 – $3.50	Capture Efficiency	Viable bridge; dependent on storage infrastructure.	^336,337^
**Electrolysis (Green)**	$3.50 – $6.00	Electricity Price	Costs falling; subsidies (e.g., US IRA) crucial for parity.	^336^
**Bio-Ethanol (ESR)**	$3.50 – $5.00	Feedstock Cost	Niche viability; requires cheap waste biomass.	^338,339^

## 7 Outlook

In the past few years, the scientific community has been experiencing an ever-growing publication on catalysts for hydrogen production. If the diversity of materials tested expands the knowledge of the nature of the various pathways, the amount of disruptive discoveries and technology grows at a different pace. The majority of research deals only with small-scale hydrogen production, and many catalysts are fancy enough not to be practical. Despite the application claims, most of the literature’s experiments emphasize the structure of the catalyst and its application at very narrow experimental conditions without a translational perspective, despite the hype around the topic. This imbalance in the literature approach might sluggish real-world applications. Transport phenomena, including mass and heat transfer considerations, for instance, are not well represented in the consulted literature, as well as the economic aspects.

It was also observed a lack of consistency in some reports regarding hydrogen production. Unfortunately, many comparisons are made between catalysts without taking into account important factors such as the system size, reaction conditions (such as pH, ionic strength, and temperature), catalyst quantity, and the time frame for calculations. All of these elements significantly affect hydrogen production rates and yield reports. Also, it is common, for example, comparing the efficacy of catalytic systems by highlighting a high turnover frequency (TOF) for a heterogeneous catalyst that relies on a significant amount of an exceptionally active support, but calculating the TOF using only the small amount of what is considered the “actual catalyst,” which could be misleading when comparing to a system that utilizes a larger amount of a “pure catalyst,” but uses no support, or uses a proven nonactive one.

Since there is no universal method for setting up hydrogen production systems, it is likely that among the less-performing catalysts selected by researchers to showcase their work, there may be a few, if not many, that would perform better under the same conditions.

Considering the above-mentioned aspects, and the literature presented, some points that would be a good idea to keep in mind when working with the development of catalysts in the field of hydrogen production/release:

1- As previously noted in the literature,^340^ the optimal catalyst for a particular process may not be the most practical choice. Costly catalysts, such as those relying on rare metals, or those difficult to synthesize on a large scale and with limited recyclability, could be outperformed by less efficient but more industrial-adapted catalysts. That approach does not mean disregarding fundamental research but should prevent the urge for scientists to try to sell their ideas as commercial products when they are still far from real-world applications. Sticking to the facts should always be the best choice: there is no need to researchers pretend to be saving the world on every single paper;

2- The environmental impact of the catalyst synthesis process should be considered, as noted by Zimmerman et al.^341^ The depletion of natural resources, energy consumption, and toxicity of the components all play a significant role in the efforts for a sustainable future. Hence, it is essential to focus not only on the result, but also on the methods employed in achieving it;

3- The various parameters that can significantly affect hydrogen production when comparing catalytic systems must be taken into account. Specifically, when evaluating catalysts utilized in the same pathway, it is essential to consider identical conditions such as the mass of the catalyst, the volume of the system, the stirring, the method for recording the results, and more. Even the reactor’s material, for instance, can impact the heat transfer in small systems and short reaction times, giving different results for the kinetics of hydrogen production even using the same catalyst.^342^ Neglecting these factors may result in erroneous conclusions regarding the superiority of one catalyst over another;

4- When considering the catalyst in heterogeneous systems, it is important to note that the support is rarely completely inert. Typically, the support has a positive effect (and sometimes an outstanding one) that must be considered when reporting the amount of catalyst used. In larger systems, the volume and mass of the catalyst can be critical factors that impact real-world applications due to concerns around transportation, handling, and equipment layout, among others.^343–348^
